# Genomic insights into the recent evolution and biodiversity of Italian sheep breeds

**DOI:** 10.1007/s00335-025-10170-8

**Published:** 2025-11-22

**Authors:** Arianna Bionda, Alessio Negro, Viviana Floridia, Francesca Maria Sarti, Silverio Grande, Paola Crepaldi

**Affiliations:** 1 Dipartimento di Scienze Agrarie e Ambientali – Produzione, Territorio, Agroenergia, Università Degli Studi Di Milano, Milan, Italy; 2 Ufficio Studi, Associazione Nazionale Della Pastorizia (Asso.Na.Pa.), Rome, Italy; 3 Dipartimento di Scienze Veterinarie, Università Degli Studi Di Messina, Messina, Italy; 4 Dipartimento di Scienze Agrarie, Alimentari ed Ambientali, Università Degli Studi Di Perugia, Perugia, Italy

## Abstract

Italy hosts a remarkable ovine biodiversity shaped by centuries of history, regional traditions, and environmental heterogeneity. This diversity sustains agricultural production as well as ecosystem services and cultural heritage. Yet, many local breeds are undergoing severe demographic decline. To explore these dynamics, we analyzed census data from all registered Italian sheep, which revealed highly variable situations across breeds but confirmed that most are currently at risk of extinction. To complement this picture, we genotyped 34 Italian sheep populations using the Ovine50K BeadChip and compared them with foreign breeds with recognized herd books in Italy. Genomic analyses of diversity (including inbreeding and effective population size), population structure, and genomic background provided insights into the state of genetic variation and relationships among breeds, including patterns of introgression. By comparing these results with data from populations sampled twenty years ago, we assessed temporal changes in diversity, genomic background, and selection signatures. Fst analyses highlighted genomic regions that have undergone the most marked shifts, allowing us to explore associated genes and QTLs. Correlations between Fst and environmental changes across 20 variables further emphasized the role of local adaptation in shaping genomic landscapes. In addition, local ancestry inference in two breeds (Gentile di Puglia and Nera di Arbus) with evidence of recent admixture identified genomic regions influenced by gene flow. Overall, our study illustrates the complex evolutionary dynamics of Italian sheep breeds and underscores the importance of integrating demographic analyses with genomic tools to guide their conservation and sustainable management.

**Supplementary Information:**

The online version contains supplementary material available at 10.1007/s00335-025-10170-8.

## Introduction

Livestock genetic variability plays a pivotal role in ecosystem conservation, land management, and climate change mitigation. In recent decades, breed conservation has gained importance as the biodiversity embedded in domesticated animals is increasingly recognized as essential for maintaining overall biodiversity (United Nations 1992; Sponenberg et al. 2018; FAO 2019).

FAO’s guidelines for the conservation of animal genetic resources emphasize improving knowledge of breeds worldwide, considering their economic and geographic context, genetic and economic value, and extinction risk, with growing reliance on molecular technologies (FAO 2007a, 2015a, b; Ajmone-Marsan et al. 2023). Globally, over 7000 local breeds are reported, but the risk status of about 60% remains unknown; of the breeds with known status, nearly 70% are considered at risk of extinction (DAD-IS, www.fao.org/dad-is, accessed 22/08/2025).

Several factors drive livestock diversity: (i) evolutionary forces, such as mutation, genetic drift, gene flow, and selection; (ii) geographic distribution and adaptation to specific environments and farming systems; (iii) economic and socio-cultural context; and (iv) breeding plans and technologies, including the use of artificial insemination, crossbreeding, and the introduction of exotic breeds to improve performance (FAO 2015b; Leroy et al. 2016). Government policies and strategies also play a major role, shaped by economic and cultural factors, and can strongly affect livestock diversity and sustainability (Cao et al. 2021; Martyniuk 2021). Thus, breeds are dynamic entities, continuously evolving under diverse forces that drive genetic change (FAO 2007b). Strengthening the management of animal genetic resources requires long-term investment and strategies that ensure both genetic and socio-economic sustainability of local breeds (FAO 2007a; Lauvie et al. 2015).

Italy, thanks to its history, traditions, and environmental variability, harbors exceptional biodiversity in all livestock species (Cortellari et al. 2021; Jones et al. 2022). The Italian Sheep and Goat Breeders Association (Asso.Na.Pa.), recognized by the Ministry of Agriculture, manages herd books for 73 sheep breeds, 63 of which are considered local (51 under conservation programs). The remaining breeds are of foreign origin but are also included in conservation programs (www.assonapa.it). Italian sheep breeds are particularly important as reservoirs of genetic variability contributing to food security, environmental preservation, and the rural economy, especially in mountainous and hilly areas (Bionda et al. 2024, 2025). However, these breeds face challenges including competition with cosmopolitan breeds, inbreeding due to small population sizes, outcrossing to improve productivity, and climate change (Bionda et al. 2024, 2025). 

Most Italian sheep breeds are used for milk and meat production. Milk is primarily destined for cheese-making, including nine PDO Pecorino cheeses and other traditional products. Regarding meat, two PGI labels—*Agnello del Centro Italia* and *Agnello di Sardegna*—enhance the value of sheep production (https://www.qualigeo.eu/). Conversely, wool production remains underexploited due to structural market issues, although regional projects aim to revive its role in the textile sector. Beyond production, sheep—particularly local populations—provide non-material benefits such as ecosystem services and socio-cultural and historical value (Ripoll-Bosch et al. 2013; Battaglini et al. 2014; FAO 2016).

A central activity of Asso.Na.Pa. is monitoring sheep biodiversity across the Italian peninsula and islands. Since its establishment, the association has tracked breed distribution and census trends using pedigree data. Each year, census data on males and females are reported by the Ministry to the National Focal Point of FAO, which updates extinction risk assessments in the Domestic Animal Diversity Information System (DAD-IS; https://www.fao.org/dad-is/en/). Since 2017, this work has been strengthened by two national projects—CHEESR (2017–2021) and SHEEP&GOAT (2021–2025)—funded under the National Rural Development Plan (PSRN, submeasure 10.2) and coordinated by Asso.Na.Pa. These projects have introduced genomic tools for both biodiversity conservation and selection, generating extensive genomic characterization of 34 Italian sheep breeds.

This study investigates the genomic landscape of Italian sheep by examining the relationships among national breeds and their connections with foreign populations, providing an overview of current diversity and population structure. We also explored temporal changes by comparing the same populations sampled twenty years ago, assessing the recent evolution of genetic diversity, genomic background, and selection signatures in the context of each population’s history. In addition, we evaluated the role of environmental adaptation in shaping these patterns and analyzed introgression in two breeds to identify genomic regions affected by crossbreeding, offering insights into the dynamic forces driving the biodiversity and evolution of Italian sheep.

## Materials and methods

### Demographic data and analysis

Census data were provided by Asso.Na.Pa. and consisted of the number of registered farms and live animals calculated on December 31st of each year of the period considered (2010–2024).

The percentage change in animal and farm was calculated for each breed as the difference between the values recorded in 2024 and those recorded in 2010, divided by the value recorded in 2010. For each breed and year, a growth rate was calculated as anti-log[logN2 − logN1)/t], where N1 and N2 are the number of animals at two consecutive censuses, and t is the time interval (in years) between the two censuses (FAO 2013). Years with 0 registrations were excluded. For each breed, ∆F for year 2024 was calculated as 1/(2*Ne) where Ne is the effective population size, calculated using classic Wright’s formula: (4*Nmales*Nfemales)/(Nmales + Nfemales) (Wright 1931; FAO 2013).

**Fig. 1:**
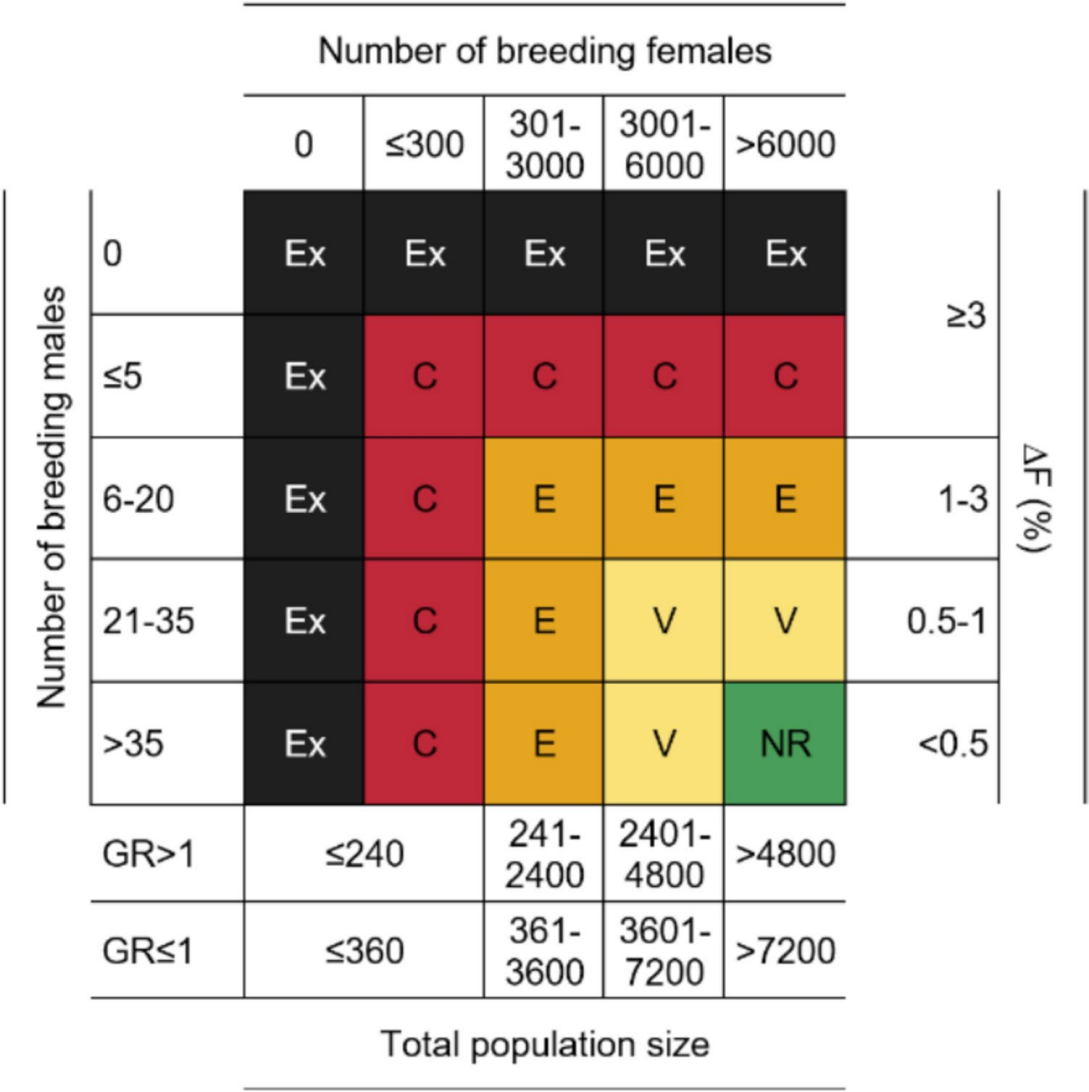
Breed risk categories according to FAO. For each breed, the risk category was assigned according to the least favorable parameter. *NR* not at risk; *V* Vulnerable; *E* Endangered; *C* Critical; *Ex* Extinct; *GR* Growth rate; *∆F* Inbreeding rate

Breed risk categories were assigned according to the criteria proposed by FAO (2013), as shown in Fig. 1, based on the number of breeding females and males, the overall population size, growth rate, and ∆F.

### Sampling

Within the CHEESR and SHEEP&GOAT projects, 7086 animals from 43 sheep breeds (selected among those with at least three registered farms at the time of sampling) were genotyped using the Illumina OvineSNP50 BeadChip (versions V1, V2, and V3). For each breed, samples were chosen among animals registered to the herd book, generally selecting one male and two unrelated females per farm, with sample size ranging from 12 to 60 animals across 4 to 20 farms. For Massese (MAS) and Comisana (COM), all samples came from the closed nucleus maintained at the Asso.Na.Pa. genetic center (https://www.assonapa.it/centro-genetico), while Sarda (SAR) animals were taken from the AGRIS Sardegna genomic flock and related farms; these data were aimed at genomic evaluations. Additional samples were collected from some breed to genomic analysis of parentage and/or wool quality. For other breeds targeted for biodiversity or wool-quality studies, such as Gentile di Puglia, Delle Langhe, Istriana, Sopravissana, and Merinizzata Italiana, sampling involved five animals per farm from 10 farms, and in some case-studies one male and two unrelated females were collected from 10 farms together with an additional group of related animals from a single farm. Datasets were obtained from pre-existing data based on routine animal recording procedures; moreover, DNA sampling for all individuals was conducted using nasal swabs and no invasive procedures were applied. Thus, in accordance with the 2010/63/EU guide and the adoption of the Law D.L. 04/03/2014, n.26 by the Italian Government, an ethical approval was not required for our study.

Given the differences in the sampling (which also included related animals) and the big sample size, a pre-selection of animals to include in the dataset was performed for COM, MAS, and SAR breeds according to the following criteria: animals were retained only if enrolled in the main section of the herd book and males with positive parentage verification. After this filtering, the resulting dataset comprised 2193 sheep from 43 different populations.

Using Plink 1.9 software (Purcell et al. 2007), we performed a quality control on this dataset, excluding individuals with a call rate below 95% and those directly related (as identified by an in-house script based on Mendelian errors), as well as SNPs located on sex chromosomes and with a call rate below 95%. For population structure analysis, we applied a minor allele frequency (MAF) filter of 0.1%, and pruned for linkage disequilibrium (LD) using Plink—*indep-pairwise* (50, 10, 0.5) function. To balance breed sample sizes, we excluded populations with fewer than 10 individuals and reduced the number of subjects to a maximum of 30 (30 being the median size per breed) using the *bite.kmeans.sampling* function of the BITEV2 v. 2.1.2 R package's (Milanesi et al. 2017). The resulting dataset included 816 individuals from 34 populations (Table 1 and Supplementary Table S1).

**Table 1:** Composition of the dataset used for genomic analyses and results for each breed

Breed code	Breed name	Raw N	N. (N. after breed size balancing)	F_ROH_ (mean ± sd)	He	Ho	Ne (SNeP)	Ne (GONE)
ALT	Altamurana	13	13 (13)	0.067 ± 0.048	0.347	0.358	14	122
APN	Appenninica	71	67 (30)	0.057 ± 0.067	0.365	0.362	35	254
BGN	Bagnolese	44	42 (30)	0.042 ± 0.050	0.376	0.368	38	871
BGS	Bergamasca	20	19 (19)	0.039 ± 0.051	0.353	0.363	20	112
BLS	Biellese	21	21 (21)	0.045 ± 0.048	0.358	0.357	23	206
BRB	Barbaresca	14	14 (14)	0.054 ± 0.083	0.354	0.359	15	143
BRI	Brianzola	24	24 (24)	0.087 ± 0.086	0.351	0.343	24	103
BRO	Brogne	37	36 (30)	0.057 ± 0.054	0.361	0.358	35	290
CIU	Pecora Ciuta	30	30 (30)	0.068 ± 0.098	0.373	0.361	30	110
COM	Comisana	34 (1438)^a^	34 (30)	0.032 ± 0.013	0.356	0.363	34	177
CRG	Corniglio	76	73 (30)	0.054 ± 0.038	0.351	0.353	32	122
DBN	Di Benevento (Quadrella)	12	12 (12)	0.146 ± 0.065	0.288	0.325	9	31
FAB	Fabrianese	130	122 (30)	0.102 ± 0.092	0.364	0.343	32	155
FRB	Frabosana	24	24 (24)	0.044 ± 0.031	0.355	0.356	29	273
GDP	Gentile di Puglia	167	158 (30)	0.034 ± 0.043	0.376	0.370	38	371
IST	Istriana (Carsolina)	81	78 (30)	0.121 ± 0.083	0.341	0.332	28	88
LMN	Lamon	23	22 (22)	0.056 ± 0.062	0.350	0.357	22	101
LPG	Alpagota	32	32 (30)	0.069 ± 0.068	0.358	0.351	37	579
LTD	Laticauda	11	11 (11)	0.085 ± 0.101	0.350	0.347	11	89
MAS	Massese	163 (1086)^a^	161 (30)	0.057 ± 0.021	0.346	0.351	35	142
MER	Merinizzata Italiana	134	129 (30)	0.037 ± 0.032	0.371	0.370	38	250
NTC	Noticiana	17	14 (14)	0.082 ± 0.041	0.323	0.340	14	72
ODL	Delle Langhe	163	157 (30)	0.094 ± 0.053	0.337	0.338	35	192
PAM	Dell'Amiata	16	16 (16)	0.069 ± 0.071	0.367	0.358	18	165
PCC	Pecora di Corteno	19	17 (17)	0.080 ± 0.061	0.347	0.344	17	63
PMR	Pomarancina	14	14 (14)	0.044 ± 0.028	0.351	0.366	15	122
PNA	Nera di Arbus	65	63 (30)	0.078 ± 0.072	0.362	0.344	38	459
SAR	Sarda	515 (3047)^a^	495 (30)	0.066 ± 0.044	0.348	0.346	38	351
SMN	Sambucana	30	30 (30)	0.039 ± 0.023	0.359	0.366	37	273
SPV	Sopravissana	34	33 (30)	0.052 ± 0.061	0.370	0.366	34	153
SVR	Savoiarda	15	13 (13)	0.105 ± 0.062	0.329	0.340	13	101
TCL	Tacola	51	50 (30)	0.023 ± 0.026	0.366	0.366	40	1699
VCN	Vicentina (Foza)	17	16 (16)	0.077 ± 0.058	0.333	0.345	16	71
VLC	Valle del Belice	28	26 (26)	0.057 ± 0.048	0.360	0.356	31	375

Nine breeds were excluded from analysis because including fewer than ten individuals after quality control and exclusion of relatives: Cornella bianca, Garfagnina bianca, Lacaune, Moscia Leccese, Schwarznasenschaf, Suffolk, Sciara-Moscia calabrese, Villnoesser schaf-Fiemmese, and Zerasca

F_ROH_: inbreeding based on runs of homozygosity; He: expected heterozygosity; Ho: observed heterozygosity; Ne: effective population size

^a^The number in parenthesis refers to the initial sample size, before filtering procedures reported in Materials and methods section

### Population structure and phylogenomic relationships

To investigate population structure, we performed a multidimensional scaling analysis (MDS) with Plink v1.9 using*—mds-plot eigvals –cluster*, setting a number of dimensions equal to the number of individuals. To better visualize both local and global structures and relationships, we further reduced dimensionality using a Potential of Heat-diffusion for Affinity-based Transition Embedding (PHATE) algorithm, as implemented in phateR v1.0.7 library (Moon et al. 2019), using the first 20 principal components (PCs) from the MDS and applying the following parameters: knn = 34 (equal to the number of analyzed breeds), decay = 100, and gamma = 0.

Phylogenetic trees were constructed using population-level bootstrapped Reynolds distances, calculated using an in-house script, and individual-level bootstrapped identity-by-state (IBS) distances, calculated with PHYLIP v3.697 (Felsenstein 1989). The trees were visualized using ggtree v3.10.1 R package (Xu et al. 2022). Moreover, we used Treemix v1.13 (Pickrell and Pritchard 2012) to investigate historical gene flow, testing models with 0 to 20 migration events. Migrations were also assessed using *f3* statistics.

After phasing data with Beagle v4.1 (Browning and Browning 2007), we analyzed haplotype sharing based on identity-by-descent (IBD) with RefinedIBD v3.1 (Browning and Browning 2013), applying a 40 Mb long sliding-window with 0.15 Mb trimming. We retained segments that were at least 1.5 Mb long and with a minimum LOD score of 3.0. Segments shared between individuals of different breeds were analyzed, and pairwise medians calculated. A value of 0 was assigned to pairs of populations sharing no segments. The top 5% haplotype sharing among breeds was visualized using circlize v0.4.16 R package (Gu et al. 2014).

The individual genetic background was analyzed with ADMIXTURE v1.3 (Alexander and Lange 2011), testing a number of clusters (K) from 2 to 35. The best-fitting model was selected based on the lowest five-fold cross-validation error (c-v). For each individual, ancestry fractions (Q-values) for each cluster were calculated.

### Genetic diversity and inbreeding

Genomic diversity was assessed for each population by calculating observed and expected heterozygosity (Ho and He, respectively) using Plink v1.9. Plink was also used to detect runs of homozygosity (ROHs) with a sliding window approach. As suggested by Meyermans et al. (2020), no MAF or LD pruning was applied for this analysis; however, we excluded direct relatives and duplicated animals but did not balanced breed sizes, resulting in a dataset of 2066 individuals and 45,740 SNPs. The following parameters were used: homozyg-density 73, homozyg-gap 500, homozyg-kb 1000, homozyg-snp 49, homozyg-window-het 0, homozyg-window-missing 2, homozyg-window-snp 49. Specifically, the minimum number of SNPs defining both a ROH and the window size was calculated using L parameter, whereas the density parameter was identified as the minimal value that maximized genome coverage, which, with this setting, reached 98.4% (Meyermans et al. 2020). A ROH-based inbreeding coefficient (F_ROH_) was calculated for each individual according to McQuillan’s formula (McQuillan et al. 2008), both for all detected ROH segments and by ROH length class (1–2 Mb, 2–4 Mb, 4–8 Mb, 8–16 Mb, and >16 Mb), allowing for an estimation of the timing of inbreeding events (Curik et al. 2014).

The effective population size (N_e_) of all populations was calculated using two LD-based software tools: SNeP v1.1 (Barbato et al. 2015), which analyzed SNPs spaced between 280 Kb and 20 Mb and applied the Sved and Feldman recombination rate correction (Sved and Feldman 1973); and GONE v1.0 (Santiago et al. 2020), using default parameters. In both cases, only generations 1 to 100 were plotted.

### Exploring recent evolution in Italian sheep breed genome

To explore the genomic changes that occurred in Italian sheep populations over recent decades, we compared our data with those from the Biovita project (Ciani et al. 2014), which includes 492 sheep from 20 Italian breeds sampled from 2002 to 2009. For consistency, we combined it with the same individuals included in our balanced dataset, applying to this merged dataset the same quality filtering, including the exclusion of relatives and the sample size balancing of the Altamurana (ALT) breed, which was sampled in two different locations (see Ciani et al. 2014), thus resulting in more than 30 individuals (that was set as the maximum breed size). The final dataset consisted of 1281 individuals and 39,418 SNPs, 94% of which were also present in the first analysed dataset (Supplementary Table S1). Among the included breeds, 18 were present in both the datasets, two (Pinzirita—PNZbv and Leccese—LCCbv) only in the Biovita dataset, and 16 only in ours.

On this dataset, we performed MDS, Admixture, F_ROH_, and heterozygosity analyses as previously described. Additionally, we used PLINK 1.9 to conduct Fst analysis on the breeds common to both datasets, in order to identify genomic regions that have changed the most over time. For the SNPs that, for each comparison, were associated with the top 1% absolute Fst values, we annotated and analyzed genes located within a ±20 kb window around the associated loci, corresponding, approximately, to the distance at which LD halves in sheep species (Kijas et al. 2014). A gene ontology (GO) enrichment analysis was performed for the set of genes associated with each breed. Using GeneCodis v.4 platform (Garcia-Moreno et al. 2022), these genes were compared against a background set of genes that were intercepted by the windows around all the possible SNPs of the chip after quality control. GO terms were identified based on the annotation of both *Homo sapiens* and *Bos taurus*, it being the closest available species to sheep, and a significant threshold of p-values adjusted with Benjamini–Hochberg correction of 0.05 was applied. Significant GO terms were grouped according to semantic similarity with GO-Figure! v1.0.2 (Reijnders and Waterhouse 2021). Additionally, a list of sheep quantitative trait loci (QTLs) was retrieved from the Sheep QTLdb (Release 56, including 5417 QTL data) (Hu et al. 2022). Those QTLs that fell within the window around the identified SNPs were analyzed. To account for the different number of annotated QTLs per type, we assessed whether the identified SNPs for each breed were significantly enriched in known ovine QTLs. To do this, we determined the total number of SNPs on the genotyping array (N) or among the identified SNPs for each breed (n) that overlapped any QTL annotated in sheep as well as the total number of SNPs on the array (K) or among the identified SNPs for each breed (k) overlapping each specific QTL type. Enrichment p-values were calculated using the hypergeometric test through *phyper(k-1, K, N-K, n, lower.tail* = *FALSE)* function in R, which tests the probability of observing at least k overlapping SNPs in the breed-specific set. Multiple testing correction was applied using the Benjamini–Hochberg false discovery rate (FDR) method with *p.adjust* R function.

To investigate the possible influence of climate change on genomic variation, we also examined the correlation between Fst values of all SNPs and changes in 20 climatic variables (Supplementary Table S2) across the breeding ranges of the analyzed populations. Specifically, we retrieved the annual means of 19 bioclimatic variables corresponding to those of the WorldClim dataset and an aridity index from the “Global bioclimatic indicators from 1979 to 2018 derived from reanalysis” via the Copernicus platform (Copernicus Climate Chnage Service 2021; Wouters 2021). These data covered the period 1979–2018 at a resolution of 0.5° × 0.5°.

For each variable, we calculated the median value in the first half of the time span (1979–1998), which we associated with the historical Biovita samples, and in the second half (1999–2018), associated with the more recent samples from our study. We then computed the difference (Δ) between these two periods for each pixel across the study area. Using farm geolocation data described in Bionda et al. (2024), we extracted the average values for each period and their differences at each farm location, considering a 10 km-diameter buffer around them, and then calculated the breed-level means.

This approach yielded, for each breed, a Δ (change) value for each of the 20 climatic variables, representing the environmental shift between the sampling periods. We then tested the correlation between Fst values at each SNP and the corresponding Δ values of the climatic variables using Pearson correlation. Resulting p-values were adjusted for multiple testing using Benjamini–Hochberg procedure as implemented in *p.adjust* function from stats R package.

We further investigated SNPs that showed a statistically significant association with at least one climatic variable and that also fell within the top 1% of Fst values for at least one breed. Genes and QTLs within a ±20 kb window around the SNPs associated with each climatic variable were analyzed as described above.

### Comparison of Italian local breeds and foreign breeds with Italian herd books

To assess the possible influence on the genomic background of Italian local breeds, a comparison was also done with publicly available data of breeds of foreign origin recognized in Italian herd books (Kijas et al. 2012; Ciani et al. 2015; Rochus et al. 2017), namely: Berrichon du Cher (BRC_FR), Charollais (CHA_FR), Île-de-France (IDF_FR), Lacaune (LAC_FR and LAM_FR for dairy and meat varieties, respectively), Mouton d’Ouessant (OUE_FR), and Romanov (ROM_FR) from France; Suffolk, originated in Great Britain (however, only data sampled in France were available, and therefore called it SUF_FR); Texel, originated in the Netherlands (GTX_NL and STX_NL for German and Scottish Texel, and TEX_FR for Texel sampled in France); and East-Friesian from Germany (EFW_DE and EFB_DE for white and brown variety, respectively). A maximum of 30 unrelated animals per population was included.

We merged these data both with the individuals included in the present Italian sheep dataset (obtaining a final dataset of 1114 sheep and 37,597 SNPs after the same quality control procedure described above, with 97% SNPs in common with the Italian dataset), and with those included in the Biovita dataset (final dataset of 753 individuals and 32,997 SNPs) to investigate possible differences between the relationship of foreign breeds with present and past Italian population (Supplementary Table S1). Specifically, we performed MDS, IBD-based haplotype sharing, and admixture analyses.

### Local ancestry inference analysis

To investigate introgression in Nera di Arbus (PNA) and Gentile di Puglia (GDP) breeds at the chromosome-level, local ancestry inference (LAI) was analysed using ELAI v1.01, which uses a two-layer hidden Markov model (Guan 2014). Haplotypes in the reference population are used to define features of each small genomic region in the target populations. In the LAI analysis, GDP and PNA breeds were considered as targets as they appeared significantly more introgressed by the Merinizzata Italiana (MER) and Sarda (SAR) than in the past GDPbv and PNAbv samples, which therefore were considered as reference populations. In particular, to verify that the introgressed population in GDP was the MER, and not the IDF_FR, we also performed MDS, MDS, IBD-based haplotype sharing, admixture, and supervised admixture analyses on a subset of breeds, that were selected among those appearing closer to the GDP in the initial admixture.

LAI analyses were performed using the following parameters: 20 expectation maximization steps (-s), 2 upper clusters (-C), 10 lower clusters (-c), and 50 previous generations before the admixture event (-mg). Those SNPs that either were not identified in a given population (–exclude-miss1) or for which the position was not recorded in the SNP position file (–exclude-nopos) were excluded from the analysis. The 99th percentile of local introgression proportion for each reference, such as MER and GDPbv in the LAI analysis of GDP and SAR and PNAbv in the LAI analysis of PNA, were considered highly introgressed. An ideogram illustrating the distribution of highly introgressed regions (99th percentile) in both target populations (GDP and PNA) was created using the Rideogram v0.2.2 R package (Hao et al. 2020). We further investigated the regions spanning 20 kb upstream and downstream of each highly introgressed SNPs to identify the associated genes and QTLs, as previously described.

## Results

### Trend in population and farm size

**Fig. 2:**
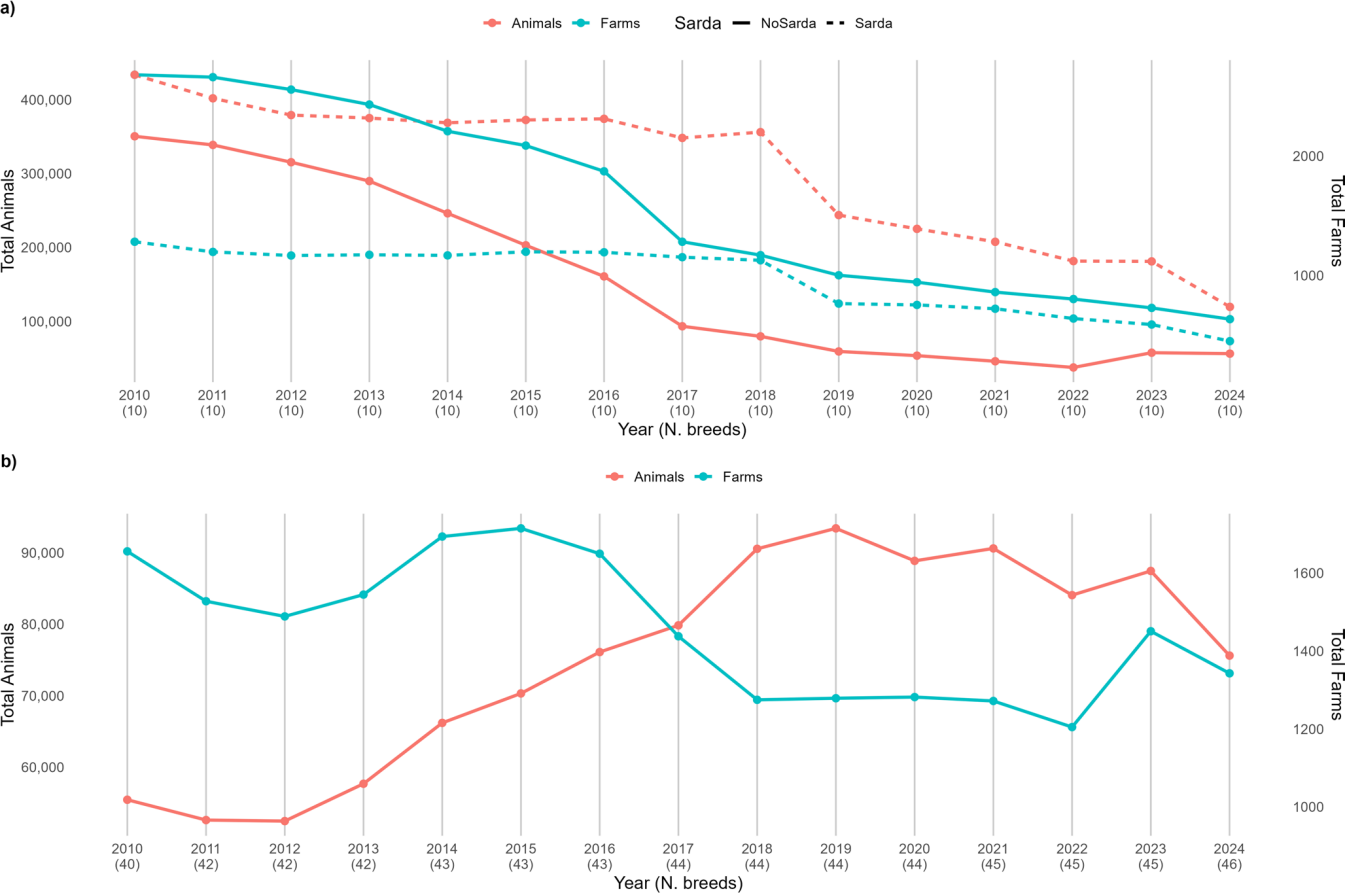
Trend in the number of registered animals and farms for breeds under selection programs, differentiating between Sarda and other breeds (**a**), and under conservation programs (**b**)

Overall, the registered sheep population in Italy has shown a consistently negative trend, both in terms of farms and, even more markedly, in animal numbers. This decline is mainly attributable to the reduction in the number of animals belonging to breeds under selection programs (Fig. 2a). In contrast, the overall trend for breeds under conservation programs showed a substantial increase in population size from 2012 to 2019, followed by a slow decline. As for farms, the trend has been variable, with some periods showing growth in the number of registered farms (2012–2015 and 2023), while others displayed negative (2014–2017) or stable patterns (Fig. 2b). Consequently, the percentage of the national sheep population represented by breeds under conservation increased from 7 to 30%, while the proportion of farms rose from 30 to 55%. Trends in animal and farm registrations for each breed are shown in Supplementary Fig. S1.

More than half of the breeds (29) experienced a contraction of over 10% in the number of farms, with seven losing at least half of their holdings and three becoming formally extinct. Conversely, about one-third of the breeds (20) showed an increase of at least 10% in farm numbers. Regarding animal numbers, population size increased in approximately half of the breeds (26), whereas more than one-third (22) declined by at least 10%. Among these, 18 breeds experienced a reduction of more than half of their initial population size. The average growth rate was negative for 11 breeds, positive for 24, and ranged between 0.95 and 1.05 for the remainder.

According to Ne estimates, one-quarter of populations (14) are at short-term risk of extinction (Ne < 50), while more than half (29) are at long-term risk (Ne between 50 and 500). Only five populations (Sarda, Tacola, Bagnolese, Merinizzata Italiana, and Nera di Arbus) showed Ne values exceeding 1000. For the seven populations with Ne = 0, ΔF could not be estimated. Among the remaining populations, the average ΔF was 1.07 ± 2.5%, with 37 populations showing values below 0.5% and five exceeding 3% (Table 2).

**Table 2:** Demographic data of Italian sheep breeds

Breed	N. farms 2024	∆Farms	N. animals 2024	∆Animals	Growth rate	Ne	∆F	Risk
*North-western Italy*								
Bergamasca	70	−15 (−17.6%)	17,063	1083 (6.8%)	1.09	579	0.09	NR
Biellese	16	−14 (−46.7%)	306	−1402 (−82.1%)	0.91	68	0.74	C
Brianzola	25	−12 (−32.4%)	1518	471 (45%)	1.04	353	0.14	E
Brigasca	8	−7 (−46.7%)	1179	37 (3.2%)	1.1	113	0.44	E
Delle Langhe^b^	27	−58 (−68.2%)	1840	−1190 (−39.3%)	0.97	225	0.22	E
Frabosana	43	−17 (−28.3%)	4267	1846 (76.2%)	1.05	560	0.09	V
Garessina	0	−3 (−100%)	0	−92 (−100%)	0.92	0		Ex
Pecora Ciuta	19	18 (1800%)^a^	545	544 (54,400%)^a^	2.42	264	0.19	E
Pecora di Corteno	12	−2 (−14.3%)	326	101 (44.9%)	1.04	106	0.47	C
Rosset	40	−7 (−14.9%)	214	124 (137.8%)	1.61	210	0.24	C
Saltasassi	0	−2 (−100%)	0	−24 (−100%)	1.05	0		Ex
Sambucana	56	3 (5.7%)	3445	1871 (118.9%)	1.06	523	0.1	V
Savoiarda	8	2 (33.3%)	263	182 (224.7%)	1.1	67	0.75	C
Tacola	107	58 (118.4%)	13,720	10,765 (364.3%)	1.13	2182	0.02	NR
*North-eastern Italy*								
Alpagota	42	−9 (−17.6%)	1459	209 (16.7%)	1.02	345	0.15	E
Brogne	32	7 (28%)	1220	204 (20.1%)	1.03	426	0.12	E
Cornella Bianca	8	3 (60%)	299	192 (179.4%)	1.1	95	0.53	C
Corniglio	13	−3 (−18.8%)	1297	590 (83.5%)	1.05	413	0.12	E
Istriana-Carsolina	6	0 (0%)	423	−8 (−1.9%)	1.01	69	0.73	E
Juraschaf-Giurassica	26	−16 (−38.1%)	5	−267 (−98.2%)	0.85	0		Ex
Lamon	19	10 (111.1%)	257	143 (125.4%)	1.11	127	0.39	C
Plezzana	3	−1 (−25%)	336	242 (257.4%)	1.11	135	0.37	C
Schnalserschaf	27	−9 (−25%)	35	−315 (−90%)	0.89	0		Ex
Schwarz Braunes Bergschaf	51	−129 (−71.7%)	36	−1725 (−98%)	1.13	11	4.55	C
Schwarznasenschaf	31	27 (675%)	282	269 (2069.2%)	1.4	206	0.24	C
Tiroler Bergschaf	83	−324 (−79.6%)	33	−4813 (−99.3%)	0.71	8	6.65	C
Tiroler Steinschaf	3	2 (200%)^a^	0	−6 (−100%)^a^	1.34	0		Ex
Vicentina-Foza	14	11 (366.7%)	186	88 (89.8%)	1.05	128	0.39	C
Villnoesser Schaf-Fiemmese	67	−45 (−40.2%)	579	−639 (−52.5%)	0.97	142	0.35	E
*Central Italy*								
Appenninica^b^	113	−27 (−19.3%)	5147	−4390 (−46%)	0.96	969	0.05	V
Dell’Amiata	46	42 (1050%)	3061	2983 (3824.4%)	1.72	476	0.11	E
Fabrianese^b^	29	−25 (−46.3%)	1525	−1754 (−53.5%)	0.95	317	0.16	E
Garfagnina Bianca	29	16 (123.1%)	1391	1168 (523.8%)	1.18	182	0.28	E
Massese^b^	65	−9 (−12.2%)	6121	−3108 (−33.7%)	0.98	778	0.06	V
Merinizzata Italiana^b^	113	−79 (−41.1%)	7821	−19,187 (−71%)	0.92	1424	0.04	NR
Nostrana	0	−1 (−100%)^a^	0	−1 (−100%)^a^	1	0		Ex
Pomarancina	31	4 (14.8%)	1334	654 (96.2%)	1.06	236	0.21	E
Quadricorna	2	0 (0%)^a^	52	0 (0%)^a^		50	1	C
Sopravissana	60	10 (20%)	5449	−245 (−4.3%)	1	797	0.06	V
*Southern Italy*								
Altamurana	6	1 (20%)	317	−74 (−18.9%)	1.01	149	0.34	C
Bagnolese	144	30 (26.3%)	12,983	1404 (12.1%)	1.02	2059	0.02	NR
Di Benevento-Quadrella	1	0 (0%)^a^	10	0 (0%)^a^	1.03	4	13.89	C
Gentile di Puglia	40	11 (37.9%)	3999	244 (6.5%)	1.02	983	0.05	V
Laticauda	68	9 (15.3%)	2955	−58 (−1.9%)	1	562	0.09	E
Moscia Leccese	21	5 (31.2%)	690	−751 (−52.1%)	0.96	115	0.44	E
Trimeticcia di Segezia	1	0 (0%)^a^	18	18 (Inf%)^a^	0.94	10	5	C
Sciara-Moscia Calabrese	1	0 (0%)	10	9 (900%)	1.3	6	7.81	C
Turchessa	10	6 (150%)	2222	2156 (3266.7%)	1.34	275	0.18	E
Zerasca	17	−11 (−39.3%)	851	181 (27%)	1.21	156	0.32	E
*Isles*								
Barbaresca^b^	18	−12 (−40%)	594	−1270 (−68.1%)	0.94	280	0.18	E
Comisana^b^	31	−550 (−94.7%)	1755	−50,174 (−96.6%)	0.79	345	0.14	E
Nera di Arbus	88	29 (49.2%)	7141	4764 (200.4%)	1.09	1013	0.05	NR
Noticiana	1	0 (0%)	312	310 (15,500%)	1.99	35	1.43	E
Pinzirita^b^	8	−253 (−96.9%)	0	−24,280 (−100%)	0.6	0		Ex
Sarda^b^	452	−831 (−64.8%)	119,614	−314,165 (−72.4%)	0.92	25,739	0	NR
Valle del Belice^b^	180	−1027 (−85.1%)	15,036	−191,182 (−92.7%)	1.09	20	2.5	C

FAO extinction risk categories: *NR* not at risk; *V* vulnerable; *E* endangered; *C* critical; *Ex* extinct

^a^Breeds that were recognized after 2010. For these breeds, ∆Farms and ∆Animals refers to the difference between the recognition year and 2024

^b^Breeds under selection programs; all other breeds are under conservation programs

According to the FAO classification, only 11% of breeds (6) are not at risk of extinction, whereas six are considered vulnerable, 20 endangered, and 17—about 30%—critical. Furthermore, seven populations should be regarded as extinct based on 2024 registrations (Table 2).

### Population structure and phylogenomic relationships

**Fig. 3:**
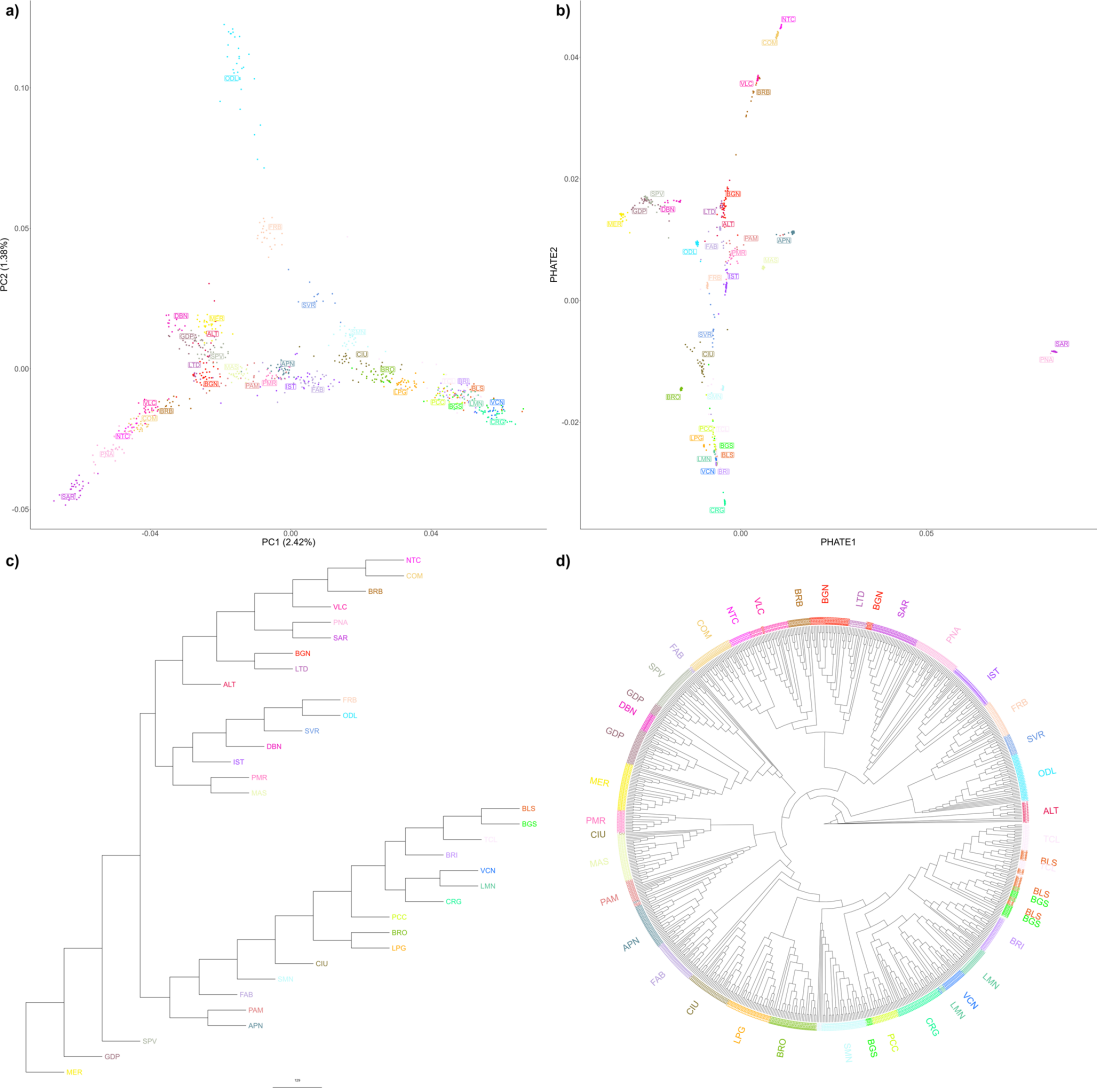
Italian sheep demographic structure. First two principal components (PCs) of multidimensional scaling analysis (**a**) and PHATE (**b**). Each point represents a subject, each color a breed. Dendrograms based on bootstrapped population-level Reynolds distances (**c**), bootstrapped individual-level identity-by-state distances (**d**)

The population structure emerging from the first three PCs of the MDS analysis and PHATE (Fig. 3a, b, respectively) strongly reflects the geographical origin and/or distribution of sheep breeds across the Italian peninsula (Bionda et al. 2024, 2025): a clear south-to-north gradient is evident along PC1 and PC2, respectively, and insular Sicilian and Sardinian breeds appear well isolated, particularly in the PHATE plot. Notably, the PHATE plot closely resembles PC1 and PC3 of the MDS, whereas PC2 mainly separates the Delle Langhe (ODL) sheep, and to a lesser extent two other Piedmontese breeds—Frabosana (FRB) and Savoiarda (SVR)—from the rest of the individuals. Notably, these breeds— especially the ODL—are slightly shifted towards southern populations in the PHATE plot.

Reynolds distances among populations, represented in Fig. 3c, similarly divide the populations into two main branches: one grouping the insular and southern Italian breeds (top), and the other grouping the northern ones (bottom). However, ODL, FRB, and SVR, as well as Istriana-Carsolina (IST), are located near the central-southern populations, reflecting the pattern seen in the MDS.

When analysing individual identity-by-state (IBS) distances (Fig. 3d), these same populations appear at the base of the tree, while all the others follow a south-to-north gradient. Individuals from the same breed are generally placed contiguously and without substantial intermixing with other populations, with a few exceptions: Biellese (BLS), Tacola (TCL), and Bergamasca (BGS) subjects are intermixed to each other, with part of the latter population also located between the Pecora di Corteno (PCC) and Sambucana (SMN) breeds.

**Fig. 4:**
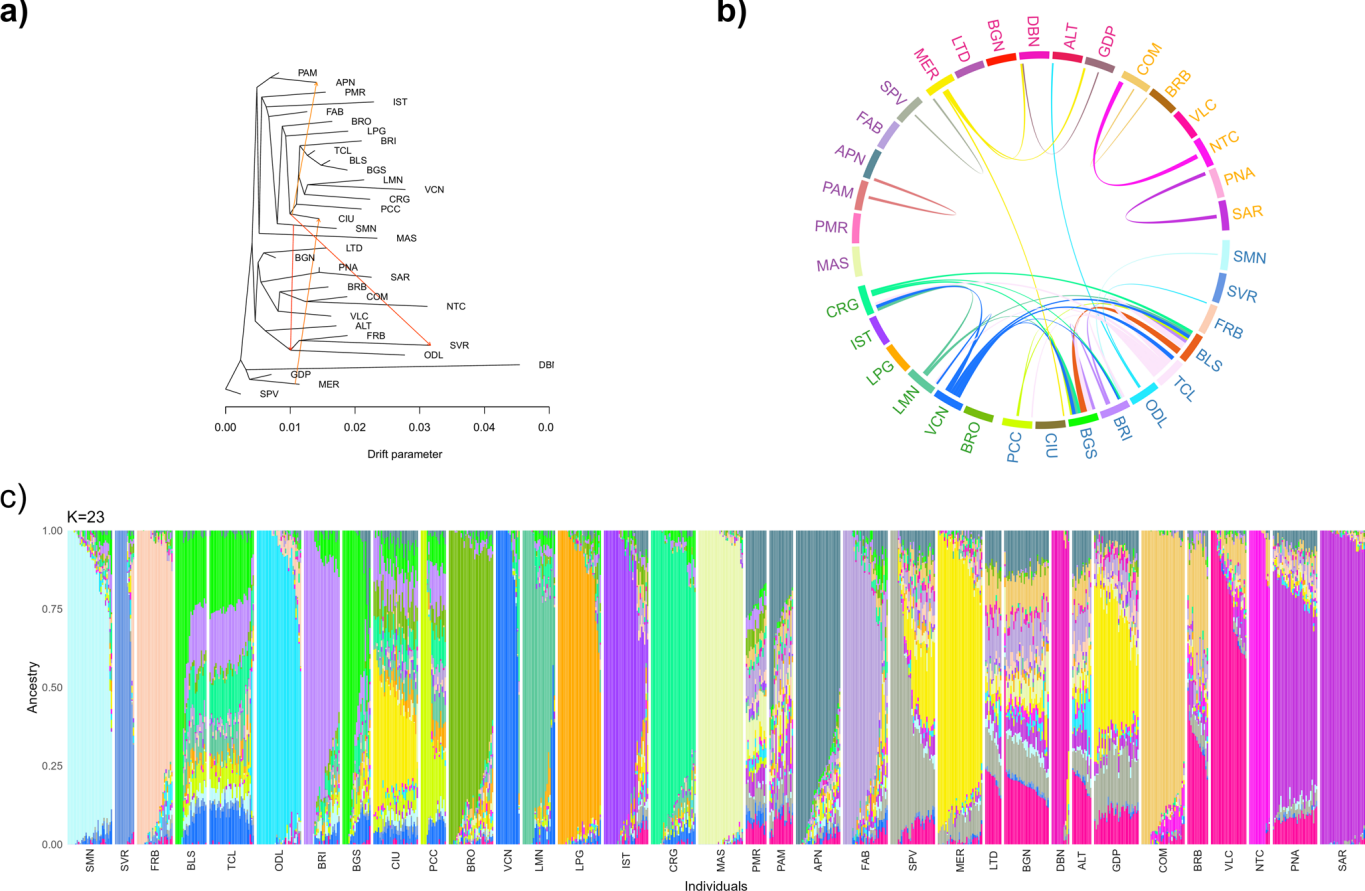
Gene flow and genomic background of Italian sheep breeds. **a** TreeMix analysis with four migration events, represented by arrows colored according to the ancestry percentage received from the donor. **b** Top 5% longest identity-by-descent-based haplotype sharing among breeds. Breed names were ordered and colored by geographic location. **c** Admixture best-fitting model, with 23 clusters (K). Breeds have been ordered by geographic location; each bar represents an individual, and each color represents a cluster

Gene flow among populations was investigated using TreeMix. A number of four migration events was chosen as the optimal configuration, and the results are shown in Fig. 4a. The strongest migration signals were observed from SMN to the node grouping the Piedmontese breeds (ODL, FRB, SVR) and from the node ancestral to Pecora Ciuta (CIU) and other northern breeds to SVR. Additional gene flows were detected from Merinizzata Italiana (MER) to CIU and from the base of the CIU branch to Appenninica (APN). However, the *f*3 test did not identify any statistically significant admixture events.

As shown in Fig. 4b, haplotype sharing is most common between geographically close breeds, especially among northern populations. In particular, TCL, BLS, BGS, Corniglio (CRG), and Vicentina-Foza (VCN) present significant sharing with several other northern breeds. Long shared segments were also found between the two Sicilian breeds Noticiana (NTC) and Comisana (COM), and between Sarda (SAR) and Nera di Arbus (PNA) from Sardinia. Interestingly, MER-CIU and ODL-Altamurana (ALT) pairs also show extensive haplotype sharing, despite having different geographic origins, as already highlighted in the TreeMix results.

At low K values (Supplementary Fig. S2), Admixture analysis clustered populations according to their geographical distribution, with the ODL cluster emerging early (K = 3). The lowest c-v (0.6094) was obtained at K = 23 (Fig. 4c), where most of the populations are characterized by distinct clusters, although many exhibit varying degrees of admixture. Consistent with previous analyses, BLS and BGS share similar ancestry, as do SAR and PNA sheep, both originating from Sardinia. Some populations appear highly admixed, with proportions similar to those of other breeds within the same geographic range. This is the case, for example, of Pomarancina (PMR) and Dell’Amiata (PAM) or Bagnolese (BGN), Laticauda (LTC) and ALT. Notably, ALT differs from the latter group because it also shows a background component similar to ODL. When a specific ALT cluster emerges (K = 25), it becomes predominant not only in ALT but also in BGN and LTD (together with COM), as well as in Barbaresca (BRB) (with COM and Valle del Belice-VLC) and Gentile di Puglia (GDP) (with MER and, to a lesser extent, Sopravissana-SPV).

### Genetic diversity and inbreeding

The average F_ROH_ in Italian sheep populations is 6.56 ± 6.1%, ranging from a value of 2.31% in TCL to 14.55% in Di Benevento (DBN) breed (Table 1). Several populations display high levels of recent inbreeding (related to ROH longer than 16 Mb), sometimes despite relatively low levels of total inbreeding, as observed in BGS and SPV (Supplementary Fig. S3a).

Estimates of Ne varied widely between methods: GONE returned values between 31 and 1699, while SNeP gave estimates from 9 to 40. These extreme estimates corresponded to the most (DBN) and least (TCL) inbred populations, respectively (Table 1 and Supplementary Fig. S3b).

### Exploring recent evolution in Italian sheep breed genome

**Fig. 5:**
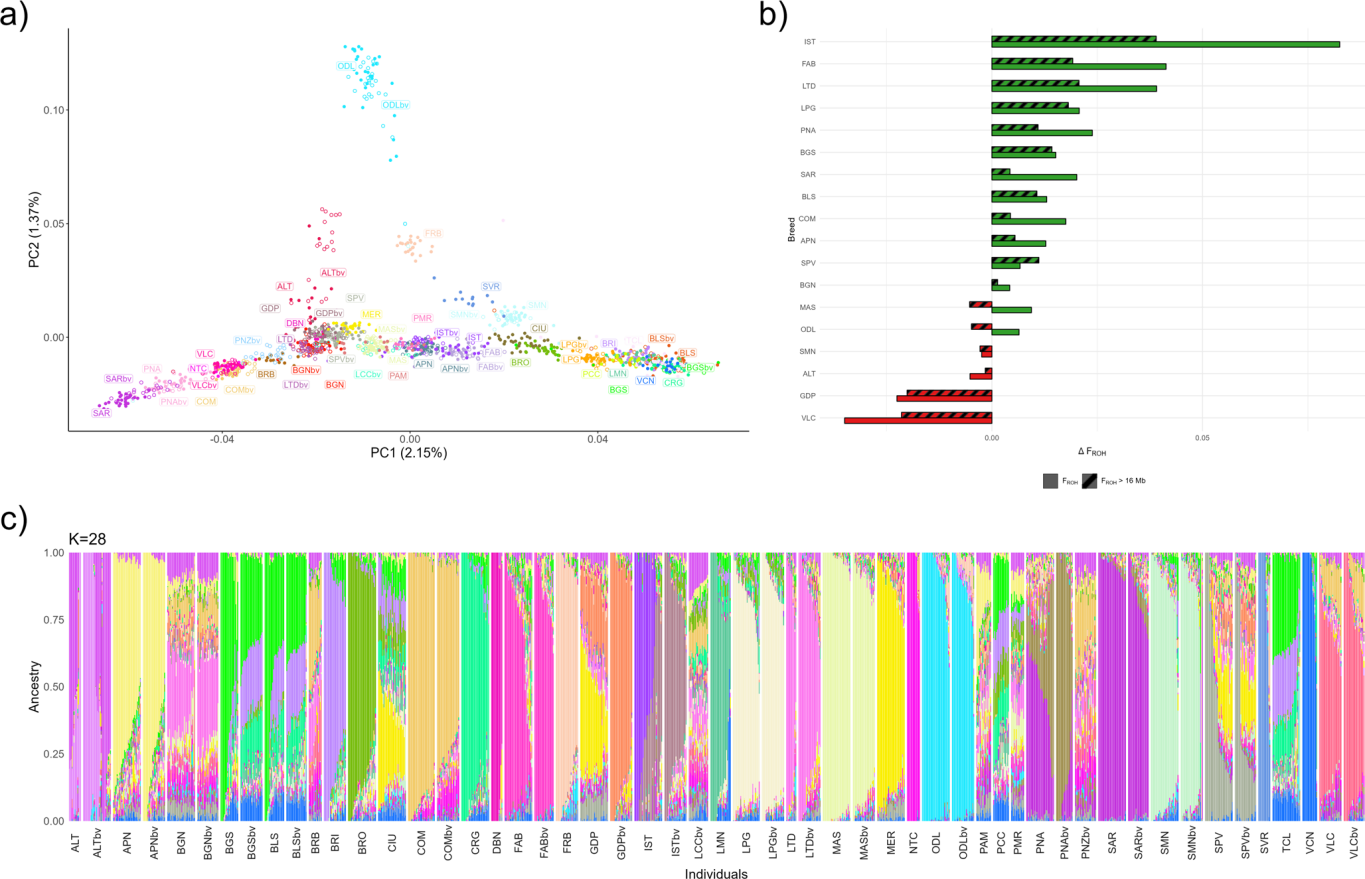
Comparison of Italian sheep breeds sampled 20 years apart. **a** Multidimensional-scaling analysis; empty and full points represent samples from Biovita and current dataset, respectively. **b** Difference in F_ROH_ between subjects of the same breed from the Biovita and current dataset. Both overall F_ROH_ and that associated with recent inbreeding events (ROH longer than 16 Mb) were included. **c** Best-fitting model of Admixture analysis. In all plots, codes including ‘bv’ refer to samples from the Biovita project, collected approximately 20 years ago. Abbreviations of breeds only present in the Biovita dataset: *LCCbv* Moscia Leccese; *PNZbv* Pinzirita

The comparison between the current dataset and the one generated within the Biovita project, which includes animals sampled about 20 years ago, through MDS analysis shows that most of the populations shared between the two datasets cluster closely, with no observable separation (Fig. 5a). The only exception concerns the ALTbv individuals, which partially overlap with the current ALT and partially diverge along PC1. However, it should be noted that the Biovita dataset included two ALT subpopulations, one from Foggia and one from Bari, whereas the vast majority of recent samples came from Bari province and none from Foggia.

In the Admixture analysis, which identified the lowest c-v, equal to 0.6018, at K = 28 (Fig. 5c, Supplementary Fig. S4), several breeds present very similar backgrounds across datasets, such as APN, BGN, COM, Alpagota (LPG), LTD, Massese (MAS), ODL, SAR, SPV, and VLC. In the case of BGS and BLS, the overall ancestral composition appears similar, with a shared prevailing cluster; however, intra-dataset individuals show stronger similarity to each other than to their counterparts in the other dataset. Fabrianese (FAB) sheep appear more admixed than FABbv. The same applies to GDP, although in this case the difference is mainly due to a marked introgression from MER in our samples, which is absent in GDPbv. Lastly, some populations show internal substructures. Consistent with the MDS results, ALTbv is divided into two subpopulations, both of which are significantly present in ALT. IST background is composed of two main ancestry clusters, one unique to IST and the other predominant in ISTbv and present in a minority of current IST individuals. PNAbv features a distinctive cluster that is still evident, though not dominant, in PNA, which appears genetically closer to SAR/SARbv population.

These observations are further supported by the comparison of F_ROH_ between the same breeds in the two datasets (Fig. 5b). In general, F_ROH_ values are higher in the current dataset than in the Biovita older data for the same breed. The greatest difference is observed in IST, now among the most inbred breeds, whereas ISTbv had one of the lowest inbreeding levels. Conversely, VLC and GDP in the current dataset are less inbred and exhibit higher heterozygosity than their Biovita counterparts, greatly depending on the increase in recent inbreeding.

### Comparison of Italian local breeds and foreign breeds with Italian herd books

**Fig. 6:**
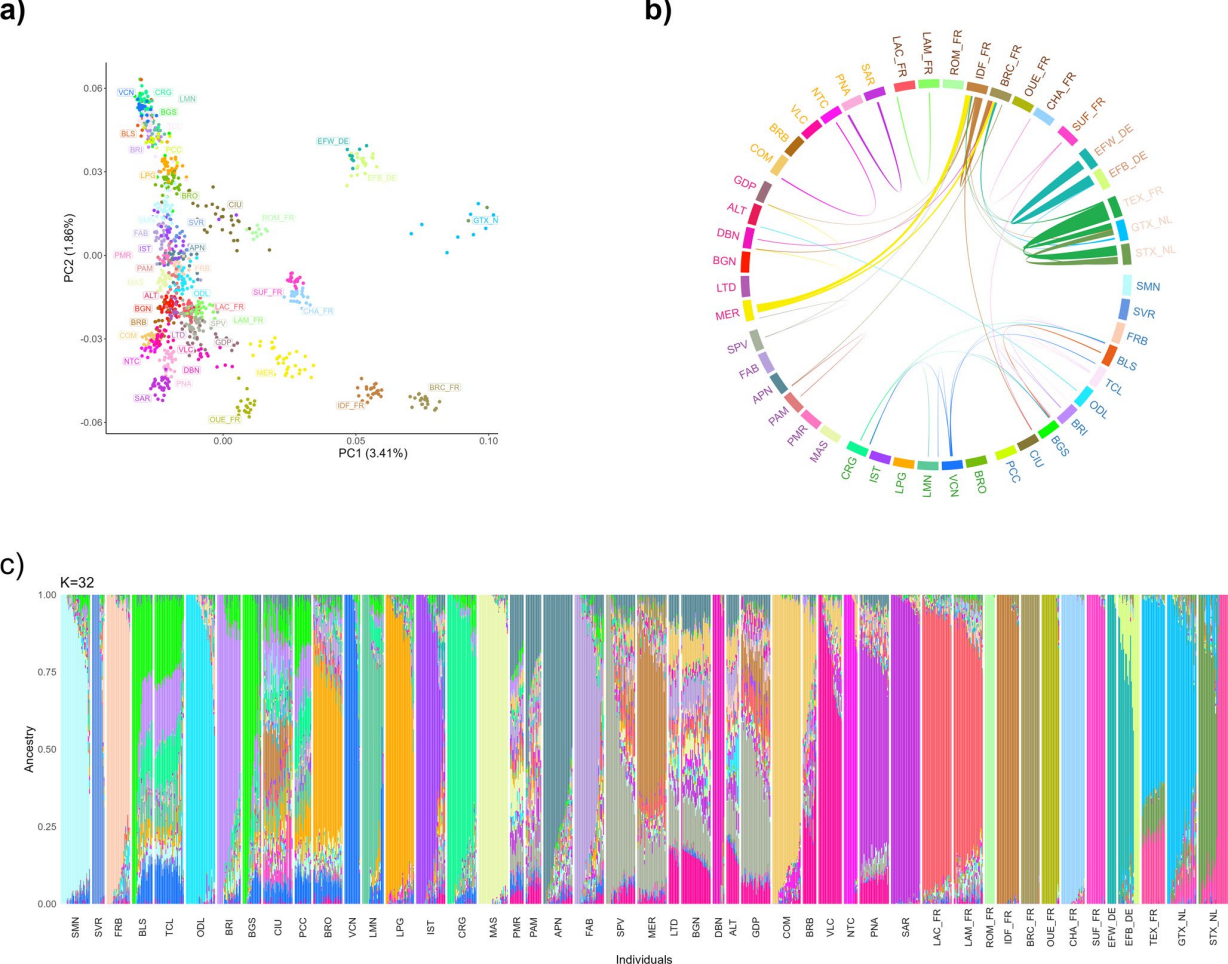
Populations structure and genomic background: a comparison between Italian and foreign breeds. **a** Multidimensional-scaling analysis. **b** 5% longest identity-by-descent-based haplotype sharing. Breeds are ordered by geographic location. **c** Best-fitting model of Admixture analysis. Breed names are ordered and colored by geographic location

The MDS plot including Italian sheep from the present-day dataset and the breeds of foreign origin recognized in Italian herd books (hereafter referred to as “foreign breeds”) (Fig. 6a) shows a clear separation of the two groups along PC1, while PC2 distributes the breeds along a geographic gradient. Texel and Friesian breeds appear clearly isolated from the Italian sheep, whereas the French breeds are positioned more closely. In particular, the two Lacaune populations (LAC_FR and LAM_FR) cluster with central Italian breeds, and the CIU and the Romanov (ROM_FR) show partial overlap. Additionally, the MER is clearly shifted toward the group of the French breeds: Île-de-France, Berrichon du Cher, and Mouton d’Ouessant (IDF_FR, BRC_FR, OUE_FR, respectively). However, PC3 clearly isolates OUE_FR from all other populations, indicating a markedly distinct genomic composition.

The results of haplotype sharing (Fig. 6b) and Admixture analyses (Fig. 6c, visualizing the results for K = 32, identified as the model with the lowest c-v = 0.6022) partially reflect the MDS findings. In general, we observed minimal introgression from most foreign breeds, with only a few exceptions. The breed showing the highest level of haplotype sharing with non-Italian breeds—particularly with IDF_FR and, to a lesser extent, BRC_FR—is the MER. This breed no longer forms its own distinct cluster but instead exhibits a genomic background composed of approximately half of the cluster maximized in IDF_FR. This component is also present in SPV and GDP, likely as a result of MER introgression. As also seen in the MDS, the CIU breed shows a foreign component, primarily derived from the Suffolk (SUF_FR) and IDF_FR breeds; however, the latter could result from MER introgression, as observed in the case of GDP.

In contrast, the same analyses conducted on the Biovita dataset reveal markedly less introgression and haplotype sharing between Italian and foreign breeds. Significant sharing is limited to the presence of Friesian populations (EFB_DE and EFW_DE) in ISTbv, and of IDF_FR in SPVbv, while it is almost entirely absent in GDPbv. However, it is important to note that several breeds showing high levels of introgression from foreign breeds in the present-day dataset, such as MER and CIU, are not included in the Biovita dataset.

### Identifying highly differentiated genomic regions via Fst

An Fst comparison between current and older Biovita populations was performed for the 18 breeds common to both datasets. For each breed pair, the SNPs within the top 1% of absolute Fst values were identified, and the corresponding genes and QTLs were investigated. The results are reported in Table 3 and Supplementary Table S3.

**Table 3:** Results of Fst comparison among individuals of the same breeds sampled 20 years apart and correlation with difference in climatic variables

Breed	Top 1% Fst	N. significant SNPs	N. genes	N. QTL	N. SNPs associated with environmental variables	N. Genes associated with environmental variables
ALT	0.16–0.37	388	152	24		
APN	0.10–0.23	393	179	20	BIO09: 2 BIO16: 3	BIO09: 1 BIO16: 1
BGN	0.14–0.40	393	178	30		
BGS	0.21–0.47	391	172	23	BIO09: 25	BIO09: 22
BLS	0.18–0.40	390	157	28	BIO09: 5 BIO16: 4	BIO09: 8 BIO16: 2
COM	0.13–0.31	390	179	14		
FAB	0.16–0.38	391	174	27		
GDP	0.28–0.58	392	162	20		
IST	0.26–0.53	391	173	32	Aridity annual mean: 2 BIO16: 117	Aridity annual mean: 3 BIO16: 112
LPG	0.12–0.33	389	169	17	BIO11: 12 BIO16: 1	BIO11: 10
LTD	0.22–0.51	391	170	22		
MAS	0.17–0.41	384	166	8	Aridity annual mean: 7	Aridity annual mean: 8
ODL	0.15–0.38	386	153	23	BIO16: 1	BIO16: 1
PNA	0.23–0.54	389	169	34		
SAR	0.12–0.51	387	165	24		
SMN	0.12–0.30	391	190	20	BIO16: 7	BIO16: 6
SPV	0.14–0.36	393	165	29	BIO16: 6	BIO16: 4
VLC	0.17–0.35	390	185	16		

**Fig. 7:**
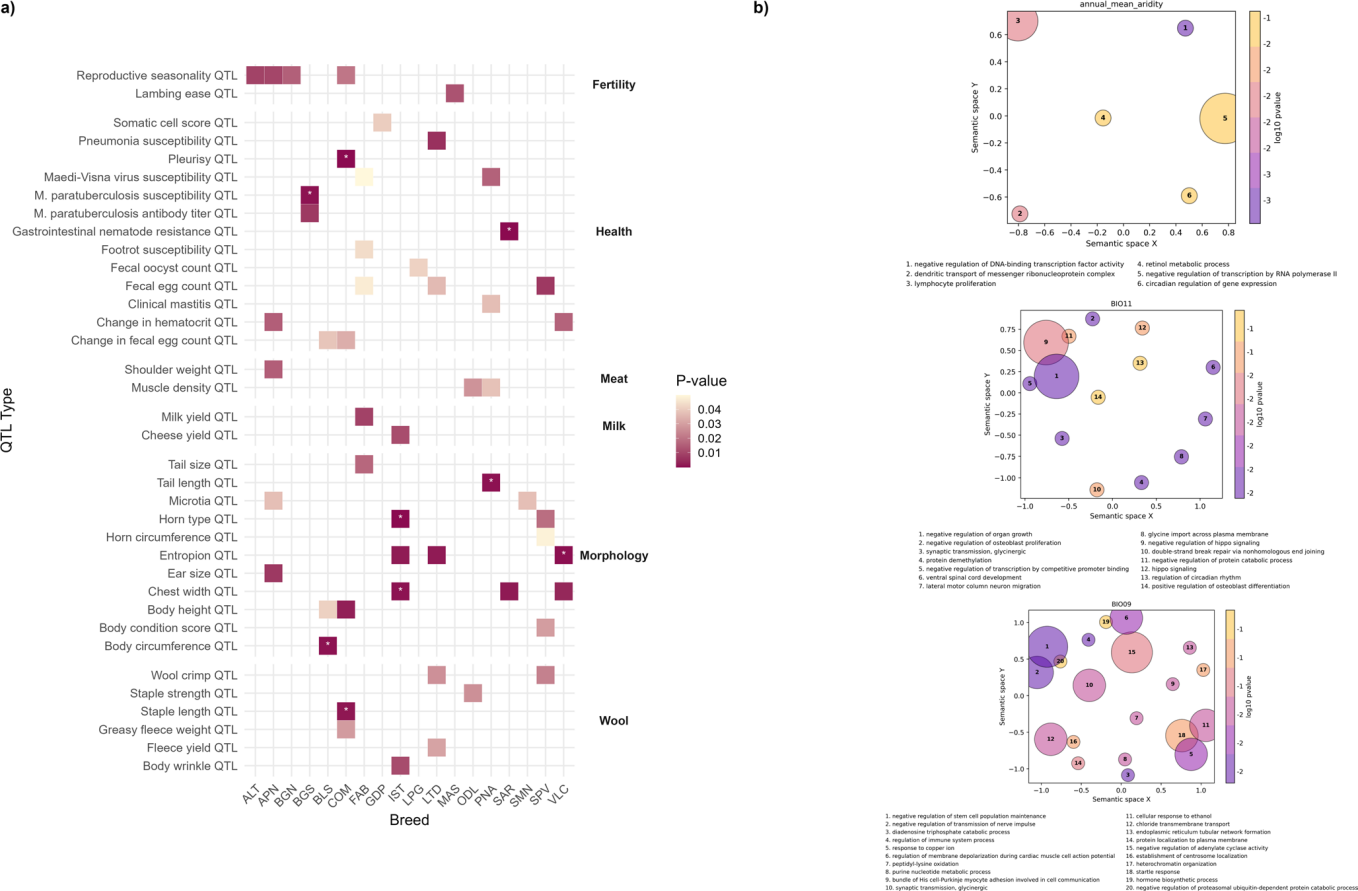
Results from the temporal comparison of subjects from the same breed sampled 20 years apart. **a** Significantly enriched QTLs by breed; QTLs with significant adjusted *p*-values are indicated with *. **b** Semantic clustering of significant GO-terms by environmental variable; results are referred only to GO-terms identified using *Bos taurus* annotation

A total of 507 distinct QTLs overlapped with the identified regions, of which 499 exhibited complete (100%) overlap. Across almost all breeds, we detected QTLs associated with weight, milk yield, milk protein and fat yield, horn type, *M. paratuberculosis* susceptibility, teat number, and faecal egg count. To account for potential biases due to the unequal number of annotated QTLs across categories, we performed a QTL enrichment analysis (Fig. 7a) for each breed. The results revealed significant associations between SNPs and fertility-related QTLs in ALT, APN, BGN, COM, and MAS. QTLs linked to udder health were detected in GDP and PNA; to parasite infestation in SAR, FAB, LTD, SPV, LPG, BLS, and COM; and to respiratory diseases in LTD, COM, and BGS. Wool-related QTLs were found in COM, IST, LTD, ODL, and SPV, while FAB and IST showed enrichment for QTLs associated with milk and cheese production. Several breeds also displayed QTLs related to morphological traits, including body size in BLS, COM, IST, SAR, and VLC.

However, after applying Benjamini–Hochberg (BH) correction for multiple testing, the only significant signals were: *M. paratuberculosis* susceptibility in BGS; body circumference in BLS; pleurisy and staple length in COM; gastrointestinal nematode resistance in SAR; tail length in PNA; horn type and chest width in IST; and entropion in VLC.

A total of 5638 genes were found in the region surrounding the identified SNPs (Supplementary Table S4). However, using these genes, only a few significant GO terms were found only for some breeds (Supplementary Table S5), mainly related to neurological development and functionality (APN, BGS, COM, FAB, LTD, LPG, and ODL); metabolic processes such as nitric oxide biosynthesis (SAR), arginine catabolism (SAR), UPD-glucose transport (ODL), peptide biosynthesis (ODL); immunity (ODL and LTD); cell adhesion (LTD and LPG); regulation of dopamine receptor signalling (ODL); and tissue and organ development (LPG and ODL).

A total of 182 SNPs resulted significantly correlated with differences in at least one environmental variable and included in the top 1% Fst distribution of at least one breed comparison (Supplementary Table S6). The SNPs thus identified were correlated with aridity annual mean (IST and MAS breeds), BIO09-mean temperature during driest quarter (APN, BGS, and BLS), BIO11-mean temperature during the driest quarter (LPG), and, with the highest number, BIO16-Precipitation during wettest quarter (APN, BLS, IST, LPG, ODL, SMN and SPV). Among the 178 genes located near these SNPs (Supplementary Table S7), the majority (112) were related to BIO16 and differentiating IST from ISTbv. Among these latter genes, some were previously reported as involved in environmental adaptation.

For example, *CD109* was identified as differentiating Iranian sheep living in cold versus hot desert areas (Saadatabadi et al. 2023), and *ST3GAL* was associated with adaptation to heat stress in Egyptian sheep (Aboul-Naga et al. 2022). In addition, we identified three aquaporin genes—*AQP2*, *AQP5*, and *AQP6*—which play key roles in renal transport and urine concentration (Agarwal and Gupta 2008; Chedid et al. 2014; Kaushik et al. 2023), as well as *EPAS1*, a hypoxia-related gene identified as a selection signature in high-altitude-adapted animals of several species (Song et al. 2016; Guo et al. 2018b).

*RACGAP1* was found to be associated with isothermality in Italian goats and to differentiate breeds living in hot versus cold climates (Cortellari et al. 2021); *ADAMTS20* was reported to distinguish goats with different coat colors (Bertolini et al. 2018); and *NPAS3* was associated with local adaptation in goats (Peng et al. 2024). In the same study, *FTO*—which we also identified among the top 1% Fst values for the SMN breed—was found to be significant. Several other studies have investigated *FTO* methylation under heat stress (Chen et al. 2023, 2025a), and linked the gene to growth, meat production and quality, and feed efficiency (Chung 2014; Jevsinek Skok et al. 2016; Dossybayev et al. 2024; Rabee et al. 2025), as well as fat metabolism, including adipose accumulation in the sheep tail (Wang et al. 2021; Chen et al. 2024a).

Additionally, among the genes correlated with BIO09 and showing high Fst values for the BGS breed, we found *RTN1*, which encodes reticulum-associated organelles known as reticulons. This gene was previously linked to sheep adaptation to heat stress (Aboul-Naga et al. 2022).

GO enrichment analysis identified 10 significant GO terms associated with genes correlated with the aridity annual mean variable when using *Bos taurus* annotation, and 20 when using human annotation (Supplementary Table S8). Semantic clustering (Fig. 7b) revealed pathways related, among others, to immune system function (e.g., lymphocyte proliferation), retinol metabolism, circadian regulation of gene expression, regulation of triglyceride metabolism and androgen receptor signaling, and blood vessel development.

For genes correlated with BIO16, only one GO term was significant when using *Homo sapiens* annotation. However, this term is of particular interest, as it relates to renal water transport and involves two aquaporin genes, *AQP2* and *AQP6*.

Among the GO term clusters (Fig. 7b) associated with genes correlated with BIO11 (16 for *Bos taurus*, 56 for *Homo sapiens* annotation), several are related to organ growth and osteoblast proliferation (including negative regulation of organ growth, positive and negative regulation of osteoblast proliferation and differentiation, and Hippo signaling), protein demethylation and catabolism, regulation of circadian rhythm, DNA repair, vocalization and walking behavior, glycine transport and glycinergic synaptic transmission, as well as numerous pathways associated with neurological development and function.

Finally, 31 significant GO terms were identified for BIO09, exclusively using *Bos taurus* annotation (Supplementary Table S8 and Fig. 7b). These include terms related to immune system regulation, response to copper and zinc ions, purine metabolism, peptidyl-lysine oxidation, regulation of stem cell population maintenance, cardiac function (e.g., membrane depolarization and His-Purkinje fiber adhesion), regulation of adenylate cyclase activity, glycinergic synaptic transmission, negative regulation of nerve impulse transmission, startle response, and hormone biosynthesis.

### Exploring introgression in Gentile di Puglia and Nera di Arbus breeds

As described above, the current PNA population appears to be more strongly introgressed by SAR compared to the same population in the Biovita dataset. Similarly, the present-day GDP shows signs of introgression from MER, with only a small portion of its genomic background shared with GDPbv. However, interpreting the situation in GDP is complicated by the evident introgression of IDF_FR into the MER breed.

Therefore, to determine whether the GDP was introgressed by MER or directly by IDF_FR, we created a subset of populations showing signs of introgression in GDP and MER, including IDF_FR and BRC_FR, along with Rambouillet (RMB_FR)—selected based on anecdotal evidence of possible crossbreeding and the occasional use of rams from French breeds by some GDP breeders.

In the MDS, GDP clusters with other Italian populations, while MER lies between the Italian sheep and IDF_FR. RMB_FR, instead, appears completely isolated along PC1. The unsupervised admixture analysis at K = 11 (the value with the lowest c-v) mirrors the results obtained from the full dataset: MER and GDP do not form specific clusters but are clearly admixed populations. The MER background is predominantly composed of the IDF_FR component, followed by SPV, whereas the GDP background resembles MER's but with stronger contributions from GDPbv and SPV. Haplotype sharing analysis shows that GDP shares relatively short segments with both IDF_FR and MER, with the latter being more prominent. Finally, in the supervised admixture analysis, where specific components were assigned to all breeds except GDP, results confirmed that the main sources of introgression into GDP were MER and SPV, rather than direct input from IDF_FR. Based on this, LAI was performed using MER as the reference population (Supplementary Fig. S5).

**Fig. 8:**
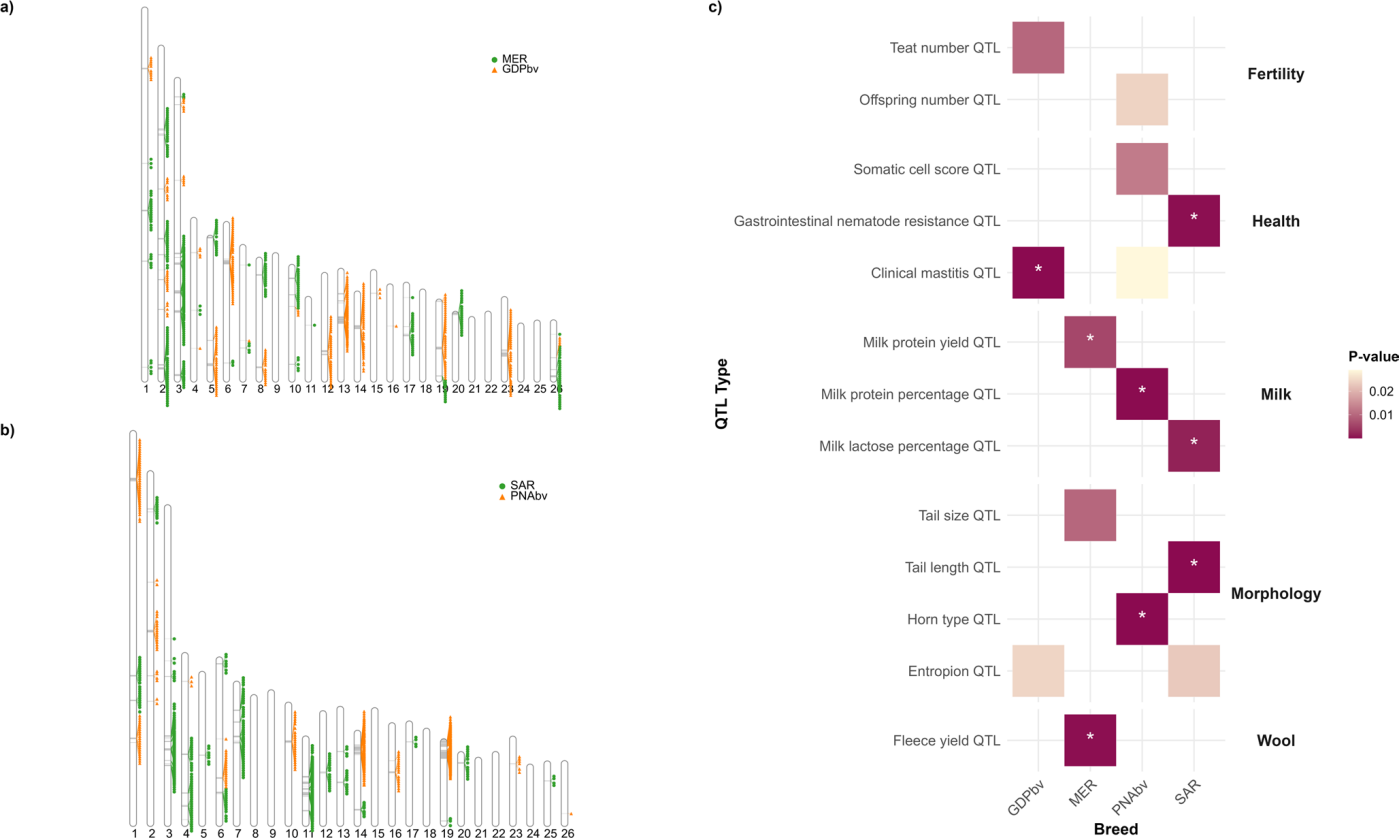
Results of local ancestry inference (LAI) on two introgressed breeds. **a** Ideogram of highly introgressed regions (99th percentile) of Gentile di Puglia (GDP), comparing older GDP samples (GDPbv, orange triangles) and Merinizzata Italiana (MER, green circles). **b** Ideogram of highly introgressed regions (99th percentile) of Nera di Arbus (PNA), comparing older PNA samples (PNAbv, orange triangles) and Sarda (SAR, green circles). **c** Enriched QTLs included in GDP genomic regions associated with GDPbv or introgressed by MER and enriched QTLs included in PNA genomic regions associated with PNAbv or introgressed by SAR. QTLs with significant adjusted *p*-values are indicated with *

The overall mean of local ancestry proportions of GDP are 0.64 ± 0.08 for MER (compared to 0.31 ± 0.06 IDF_FR, data not shown) and 0.36 ± 0.08 for GDPbv. Among the SNPs classified as highly introgressed (99th percentile), 399 and 395 were found using MER and GDPbv as reference (Fig. 8a and Supplementary Table S9), intercepting 177 and 199 genes, respectively (Supplementary Table S10).

Several genes involved in hair follicle development and wool type differentiation were found in GDP non-introgressed regions, including genes (e.g., *APCDD1*) previously reported in relation to high wool quality (Li et al. 2020b; Zhou et al. 2022). In contrast, MER-introgressed regions contain the *VDR* gene, also involved in hair follicle regulation (Zhao et al. 2021).

Most of the genes found in non-introgressed regions are related to fertility. These include *BMPR1B* and *UNC5C*, known to regulate ovulation rate and litter size in sheep (Liu et al. 2025c; Han et al. 2025; Abdurahman et al. 2025), *TCP1*, associated with male fertility in cattle (Sweett et al. 2020), and *TDRD5*, involved in germ cell development (Gangwar et al. 2024). Additionally, *DLG1*, which influences litter size (Hernández-Montiel et al. 2020), and *VDR* (Stenhouse et al. 2021) were found in MER-introgressed regions.

GDP non-introgressed regions were enriched in genes related to fat metabolism, including *LPIN1*, involved in triglyceride and fatty acid synthesis in the mammary gland and in fat tail deposition in sheep (Jin et al. 2022; Hosseini et al. 2023), and *SLC4A7*, associated with fat deposition in cattle (Martins et al. 2021). Genes associated with meat production traits were identified in both introgressed and non-introgressed regions. In MER-introgressed regions, genes included *ALDH1A1* [meat tenderness and juiciness (Gagaoua et al. 2018)], *DLG1* [feed intake (Seabury et al. 2017)], and *SLC16A7* [body growth (Abousoliman et al. 2021)]. In GDP non-introgressed regions, *APCDD1*, associated with beef meat quality (Edea et al. 2020), was found.

Finally, some genes are involved in environmental adaptation. *SLC16A7*, related to drought adaptation in cattle (Lyu et al. 2024), and *KHDRBS2* (Peng et al. 2024) were located in introgressed regions. In contrast, *SOD2*, fundamental for oxidative stress protection and lipopolysaccharide response (Hadfield et al. 2018; Shi et al. 2020; Austin et al. 2024; Wang et al. 2024b), was found in GDP non-introgressed regions.

Additionally, GDP regions introgressed from MER are enriched in QTLs related to production traits (e.g., fleece yield, milk protein yield) and tail size, whereas regions where GDPbv predominates are associated with udder health (e.g., clinical mastitis) and teat number (Fig. 8c).

Regarding the proportion of local ancestry of PNA, the overall means are 0.65 ± 0.09 and 0.35 ± 0.09 for SAR and PNAbv, respectively. Among the highly introgressed SNPs, 395 and 396 have been identified using SAR and PNAbv as references, respectively (Fig. 8b, Supplementary Fig. S6, and Supplementary Table S9), intercepting 374 and 194 genes (Supplementary Table S10).

Several genes in regions not introgressed from SAR and still associated with the older genomic background of PNA are related to fiber development and follicle biology. Among them, *EGFR* is essential for hair development and follicle integrity (Moore et al. 1991; Du Cros et al. 1992; Wynn et al. 1995; Tripurani et al. 2018; Tian et al. 2025b) and has been associated with fleece type in sheep (Zhang et al. 2025b). *EGFR* acts in part via the β-catenin pathway and, accordingly, *CTNNB1*, another gene retained in PNA, is linked to fiber diameter in Merino sheep (ZinAlabidin et al. 2025). On the other hand, several fiber-related genes were found in regions that are highly introgressed from SAR. These include a large number of keratin genes (*KRT20*, *KRT24*, *KRT32*, *KRT34*, *KRT35*, *KRT36*) (Yu et al. 2011; Sulayman et al. 2018; Duan et al. 2022; Chen et al. 2024b; Hong et al. 2024), as well as *RERE* (Arzik et al. 2023) and *STAT3* (Zhao et al. 2021), all strongly associated with wool quantity and quality.

Regarding milk production, among the genes located in introgressed regions is *STAT5A*, widely known for its role in mammary gland development and its influence on milk performance and composition, also through binding with caseins (Colitti and Farinacci 2009; Hughes and Watson 2012; Abousoliman et al. 2020; Song et al. 2022; Wang et al. 2024a). Conversely, *ACSF3*, found in a PNAbv-associated region, has been linked to goaty milk flavor (Zhang et al. 2022), and *TGFBR2* is involved in mammary morphogenesis and development (Marete et al. 2018).

Additionally, some PNAbv regions harbor genes related to mastitis, such as *C9*, *FYB* (Banos et al. 2017), and *MYD88* (El-Sayed et al. 2025). The latter is also implicated in both inflammatory responses and resistance to small ruminant lentiviruses (Sarafidou et al. 2013; Arcangeli et al. 2021). Other immune-related genes found in PNAbv-associated regions include *FOCF1*, a candidate for pneumonia resistance (Huang et al. 2025). In contrast, introgressed regions contain *STAT5A* and *CSF3*, both associated with mastitis resistance in cattle (Usman et al. 2014; Stella et al. 2018), the latter being also linked to gastrointestinal nematode resistance (Estrada-Reyes et al. 2022).

Finally, both PNAbv and introgressed regions contain genes related to fitness traits, such as fertility and environmental adaptation. Indeed, in PNAbv regions, *CTNNB1* is associated with litter size in goats and sheep (Xu et al. 2018; Zhang et al. 2018), whereas *CBFA2T3* and *DNAJA2* have been associated with heat tolerance in cattle (Li et al. 2020a; Ben-Jemaa et al. 2020; Habimana et al. 2023), and *EGFR* is linked to environmental and altitude adaptation in sheep and Tibetan goats (Rochus et al. 2018; Liu et al. 2025a). From the introgressed background, genes such as *IGFBP4* and *CHST11*, associated with fertility and prolificacy (Monget et al. 2002; Tao et al. 2021; Liu et al. 2024), were identified, as well as *TOP2A*, involved in feed efficiency in dairy sheep (Suárez-Vega et al. 2023), and genes like *NR1D1*, *KRT24*, *BRCA1*, *ADAM22*, and *ACLY*, associated with environmental adaptation in both sheep and cattle (Cheruiyot et al. 2022; Worku et al. 2023; Earnhardt-San et al. 2023; Sukhija et al. 2024; Zhu et al. 2025).

The QTL enrichment analysis (Fig. 8c) of introgressed genomic regions revealed that regions introduced into PNA from SAR are primarily associated with traits related to production (e.g., milk lactose percentage), morphology (e.g., tail length), and gastrointestinal nematode resistance. In contrast, regions derived from PNAbv include loci related to milk quality (e.g., milk protein percentage), horn morphology, and—although not significant after multiple testing correction—udder health (e.g., somatic cell score, clinical mastitis) and fertility (e.g., number of offspring).

Significant Gene Ontology (GO) terms were found only for PNA’s SAR-introgressed regions, associated with “intermediate filament organization”, and PNAbv-specific regions, associated with cardiac function (“bundle of His cell action potential” and “AV node cell action potential”, both linked to the *SCN5A* and *SCN10A* genes).

## Discussion

The Italian ovine sector is characterized by remarkable diversity, shaped over centuries by regional traditions, varied farming systems, and environmental heterogeneity. This long history has produced a complex mosaic of local breeds, each adapted to specific landscapes and serving not only productive purposes but also cultural and ecosystem functions. Understanding this complexity requires integrating demographic, genealogical, environmental, and genomic information, which allows us to capture both the current structure of the sector and the evolutionary forces—natural and human-driven—that have shaped it over recent times.

However, this rich diversity appears to be under pressure: according to official census data from Asso.Na.Pa., both the number of registered sheep and sheep-holding farms in Italy have declined over the past 15 years, reflecting trends reported by ISMEA and ISTAT (ISTAT 2015; Ismea 2024), which are based on the *Anagrafe Nazionale Zootecnica* (the official national database, for which registration is mandatory). By contrast, registration in the herd book is not mandatory (currently, sheep recorded in the herd book represent only about 5% of the national sheep population, including 4% Sarda sheep and 9% of other breeds under selection programs, but 45% of those under conservation programs), although such registration is legally required for animals to be considered purebred. For these reasons—and because the national database often contains inconsistent or missing breed information, occasionally fails to distinguish individual farms from aggregated sheep and goat holdings, and even includes farms with no animals recorded—the herd book database is the most reliable source of data for evaluating demographic trends and assessing breed risk status.

When considering only breeds officially classified as under selection, a persistent decline is evident. In contrast, data across all registered breeds under conservation show a period of growth in sheep numbers from 2012 to 2019, followed by a mild decline, while the number of farms remained quite variable. Therefore, overall, sheep belonging to local populations increased by approximately 36% between 2010 and 2024, despite the number of their farms decreasing roughly 19%. Despite still representing only a small portion of the total Italian sheep stock (30%), half of the total registered sheep farms hold individuals from local populations, confirming their fundamental role in the Italian ovine sector. Demographic trends, however, vary considerably by breed, with no clear association with either breeding purpose or geographic area. This suggests that the main factor shaping these dynamics is the variability in subsidy schemes and financial incentives granted to farmers for the conservation of local breeds.

Census-based effective population size (Ne) estimates indicate that a quarter of Italian breeds are at immediate extinction risk and over half at long-term risk according to the 50/500 rule (Franklin 1980). FAO’s more comprehensive risk assessment—considering Ne, number of breeding males and females, population growth rate, and ΔF—reinforces these concerns (FAO 2013). Only six breeds (Bagnolese, Bergamasca, Merinizzata Italiana, Nera di Arbus, Sarda, and Tacola) currently fall into a “not at risk” category. 30% of breeds are assessed as critical, and seven appear extinct, having no registered animals in 2024. However, as reported above, there are discrepancies between registered and actual animals; therefore, unregistered individuals that share morphology and genotype, despite not being recognized as purebred according to law (Reg, EU 2016/1012), may still represent important reservoirs of genetic diversity (Oldenbroek 2007). Proper registration, supported by both morphological and genomic verification, should therefore be encouraged. In fact, the 2025 herd book regulation applies a derogation to these breeds and to those classified as critically endangered, allowing the inclusion in the herd book of animals assessed as conforming to the breed standard, even if their parents are not themselves registered.

Additional factors that can exacerbate extinction risk include narrow breeding ranges (common among local Italian breeds), environmental threats, and political, social, or market pressures (Tisdell 2003; Alderson 2009; FAO 2013). Given this complex interplay of demographic, environmental, and management challenges, genomic tools have become pivotal for monitoring and managing breed diversity. Indeed, analyses of heterozygosity, ROH, and genomic Ne allow a more precise quantification of genetic health, providing insights that complement pedigree and census data (Howard et al. 2017). Our data show that F_ROH_ levels in Italian sheep vary dramatically by breed, both in terms of global value and the distribution of ROH length classes, which reflects the timing of inbreeding events (Curik et al. 2014). For instance, Pecora Ciuta and Bergamasca show moderate inbreeding, but with significant long ROH segments indicating recent inbreeding events. More alarming are breeds like Fabrianese, Istriana, and Di Benevento, which exhibit high recent and overall inbreeding, raising imminent risk of genetic erosion if breeding is not better managed. These inbreeding trends correspond well with trajectories in genomic Ne. Most breeds display a decline in Ne approximately 6 to 40 generations ago, as seen in other domestic animals and sheep breeds (Drzaic et al. 2022; Novo et al. 2023; Mészáros et al. 2025). Exceptions include Di Benevento, which had persistently low Ne but has risen recently, and Tacola, which remained stable before its recent increase. These results are supported by the demographic data of these breeds: the Di Benevento was recently recognized as a breed (2021), but with only a few animals and a single farm currently registered to Asso.Na.Pa.; conversely, the Tacola showed a constantly positive trend in the registrations of both heads and farms.

Genomic analyses also allow for the exploration of population structure and relationships among breeds, enhancing our understanding of their origin, evolution, and management. The results of MDS, PHATE, and Reynolds genetic distances reveal a clear geographic differentiation among Italian sheep breeds, consistent with previous studies (Kijas et al. 2012; Ciani et al. 2014, 2015; Ceccobelli et al. 2023). However, breed-specific patterns already emerge at this level. For instance, the Delle Langhe breed exhibits an early separation from the other populations and shows genetic affinities with central-southern Italian breeds, particularly the Altamurana. This pattern, previously reported by Ciani et al. (2014), may reflect historical crossbreeding practices aimed at improving the dairy aptitude of local breeds through the introduction of Delle Langhe genetics.

Data obtained from these analyses, as well as individual IBS trees, admixture, and haplotype sharing, reveal clear population differentiation among many Italian sheep breeds. Notably, most central-southern breeds appear to be genetically well-distinct from one another. In contrast, several northern breeds—excluding most of the populations from Piedmont and Veneto—share a more similar genetic background and show high levels of introgression from neighboring populations. Overall, the strongest genetic sharing is observed among breeds from the same geographic region.

Among the northern populations, the Biellese and Bergamasca breeds exhibit highly overlapping genetic backgrounds, and strong signals of introgression from these breeds are found in several others, such as the Tacola. These breeds are part of the group of Alpine meat breeds, which share several morphological traits, including the convex “*montonino”* fronto-nasal profile, long pendulous ears (except for Tacola), and a short, thin tail. The Bergamasca breed is thought to have originated in the Bergamo area of northern Italy as early as the fifth century. The modern breed descends from this ancestral population and underwent significant development during the long-range transhumance in the twelfth century, when previously isolated sheep flocks—often raised in monastic farms and valley communities—came into contact and interbred. By the thirteenth–fourteenth century, a population with the key traits of the modern Bergamasca was already well established. In the following centuries, this breed expanded to nearby provinces and eventually into central Italy, where it was raised both in pure form and as a cross with local populations. Indeed, Bergamasca is the largest-sized breed in Italy (rams weigh about 110 kg, and sheep about 80 kg) and consequently rams have been commonly used to improve meat production in other breeds, contributing to the formation of several meat-type breeds such as the Biellese, Varesina, Brianzola, Pecora di Corteno, Lamon, Alpagota, Bellunese, Tirolese, and Appenninica (Corti and Foppa 1999; Sarti 2003a, b). This historical diffusion is consistent with the significant genetic sharing observed in our analyses between Bergamasca and many other Italian meat breeds. It should be noted, however, that in this breed a marked discrepancy has emerged in recent years between the number of animals registered in Lombardy and those actually present. This decline in breeders’ interest in registration appears to be linked to the difficulties they face in providing the data required by the association, given that flocks are generally managed under nomadic systems. In 2022, however, registrations increased again following the introduction of financial incentives for the breed in the region, supporting the hypothesis that such incentives are the main driver of fluctuations in local registration trends. The Biellese takes its name from the province where it originated, an important center for the textile industry. Indeed, this breed was initially reared for both meat and wool production; however, its wool, being coarse, was primarily used for carpets and padding. Considered a subtype of the Bergamasca until the early twentieth century, it likely underwent intense crossbreeding with it, particularly following a sharp population decline in the 1960s that threatened its survival (Ciani et al. 2014; Bigi and Zanon 2020). At present, this population shows a low number of registered animals, which has decreased over the last 15 years. Although historical sources suggest that the Biellese was also crossed with other minor Piedmontese breeds such as the Savoiarda, Frabosana, and Sambucana, our results identify these as genetically well-defined and distinct populations, showing only limited signs of recent introgression. The Tacola sheep, by contrast, is thought to derive directly from the Biellese, a relationship supported by their nearly identical genomic background. However, the Tacola displays a distinctive morphological trait—markedly reduced auricular pinnae—that sets it apart from its presumed ancestor (Bigi and Zanon 2020). This characteristic favoured the recognition of the Tacola as a distinct breed on a morphological basis, as happened for other populations, allowing the farmers to access funding dedicated to livestock biodiversity support.

Among central Italian breeds, the Pomarancina and Dell’Amiata display admixed genomic backgrounds dominated by the Appenninica cluster, which is consistent with their derivation from the ancient Apennine population and following crossbreeding aimed at improving meat and wool production (Sorgentini et al. 2002; Bigi and Zanon 2020). The two breeds from the Campania region—Laticauda and Bagnolese—share a complex admixed background, similar to the Altamurana and presenting prominent contributions from breeds from central-southern Italy and Sicily. In particular, as soon as the Altamurana cluster appears, their admixture is dominated by it, with relevant portions also similar to Comisana (Sarti 2003b; Bigi and Zanon 2020).

To better understand how these populations have evolved over recent decades in response to market preferences, political and societal changes, and breeders’ decisions, we compared our data with the Biovita dataset, which includes individuals sampled about two decades ago. A concerning result is the general increase in genomic inbreeding observed across most populations. Notably, the Istriana breed, which previously showed among the lowest inbreeding levels, now exhibits high F_ROH_ values. Conversely, a decrease in F_ROH_ was observed in Gentile di Puglia and Valle del Belice, likely reflecting an increasingly admixed genomic background in these populations and/or different sampling strategies.

Although most breeds appear to have retained their genomic identity over time, a higher degree of admixture was observed in several, often not traceable to a single donor population. In some cases, we detected marked genetic differences or substructures that warrant further investigation. For example, a clear differentiation was observed in the Altamurana breed, consistent with findings reported by Ciani et al. (2014), where two distinct Altamurana subpopulations, raised in different locations, were sampled. These two subpopulations are partially distinguishable in the Admixture analysis and both contribute—at varying proportions—to the background of most recent Altamurana individuals analyzed in this study. One of these clusters is also significantly represented in other breeds, such as Bagnolese, Istriana (from the Biovita dataset), Dell’Amiata, Pomarancina, Pinzirita, and Sopravissana. However, it is important to note that at present no Altamurana sheep farms are present in Foggia, the city of origin of one of the Biovita subpopulations. Notable differences were also observed in the Bergamasca and Biellese breeds. In the Biovita dataset, individuals from both breeds shared an admixed background with similar proportions. In contrast, in the recent dataset some individuals showed a uniform genomic background consistent with the main Bergamasca–Biellese cluster, while others retained a more admixed ancestry, like the Biovita samples. Notably, all individuals with a uniform background were sampled in Ravenna, including several from the same farm, which rears both breeds, whereas the more admixed individuals originated both from Ravenna and other provinces, such as Ragusa, Forlì-Cesena, Torino, and Cuneo.

This analysis also allowed to better understand the background of the Sicilian Barbaresca: now raised in Sicily for triple-purpose production (milk, meat, and wool), it originates from the cross between the North African Barbaresca and the local Pinzirita, a process that likely began during the Saracen rule in the ninth century (Bigi and Zanon 2020). This is supported by the resemblance of Barbaresca and Pinzirita (which was only present in the Biovita dataset) admixture, despite the first presenting a higher presence of Valle del Belice and Laticauda. Notably, this population shows marked decrease in animal and farm registration in recent years, and thus should be monitored to avoid genetic erosion and excessive introgression.

The Istriana (also known as Carsolina) breed also displayed a complex structure, with one cluster found only in some of the recent individuals, while another cluster was shared by both Biovita and recent samples. This breed originated from crossbreeding between local populations inhabiting the karst regions of the northern Adriatic and sheep introduced by migrants from the Balkans, as documented since the seventeenth century. The history of this breed has been closely linked to the events of the two World Wars, with the population experiencing a dramatic decline in the post-war period due to the devastation of the territory and the advent of industrial agriculture (Paoletti and Aceto 2007). Today, a few hundreds of heads are raised in the karst areas of Italy (particularly in Friuli-Venezia Giulia), as well as in Slovenia and Croatia (Bigi and Zanon 2020).

In the Nera di Arbus sheep, all Biovita individuals shared the same predominant genomic cluster, with limited signs of Sarda introgression. In contrast, most recent samples showed a background largely dominated by Sarda ancestry. Both breeds originated in Sardinia and are used for milk and cheese production, but they differ markedly in morphology: while the Sarda is polled and white, the Nera di Arbus has black skin and fleece and retains some ancestral traits such as horns in both sexes, small ears, and smaller body size. The Sarda is the most widespread Italian sheep breed, whereas the Nera di Arbus, which is less productive, is mainly confined to the southwestern part of Sardinia, but also shows an overall positive trend in the number of registered animals in the last decade, likely thanks to the financial support to its breeding, often one of the major sources of income for these farmers. The Nera di Arbus is considered an ancestral lineage of Sardinian sheep that escaped the intense dairy selection applied to the Sarda. It survived due to its ability to exploit the marginal pastures of the hilly region and because of its value in the local handicraft traditions of Arbus, which utilize both horn and fleece (Piras et al. 2009). However, it came close to extinction, and only relatively recent conservation initiatives led to its recovery and official recognition in 2008.

Another case of worrying introgression is that of the Gentile di Puglia. While Biovita samples showed a homogeneous genomic background dominated by a single cluster, recent samples displayed a markedly admixed profile. The dominant ancestry in these modern individuals is derived from the Merinizzata Italiana, followed by residual contributions from the historical Gentile di Puglia and Sopravissana clusters. While it cannot be ruled out that these results may partly be due to sampling of individuals not fully adhering to the breed standard, the fact that all sampled animals were registered to the herd book and that the pattern is consistent across individuals suggests that recent unsupervised crossbreeding with Merinizzata Italiana is a plausible explanation. In contrast, the Sopravissana shows a more stable picture across time: some individuals exhibit a homogeneous background, while others show admixture, mainly with Merinizzata Italiana. The history of these two breeds helps explain these patterns. The Gentile di Puglia originated from an Apulian population renowned for its fine wool since Roman times. During the Spanish domination (fifteenth century), this local population was crossed with Merino rams imported from Spain by the Aragonese and Bourbon monarchies. The resulting breed was involved in horizontal transhumance along the “tratturi” routes between Puglia and the Apennines of Abruzzo and later spread into other central and southern regions, contributing to the development of several local breeds. The Sopravissana, on the other hand, descends from the local Vissana population, which underwent "merinization" between 1750 and 1943. This process involved crossbreeding with Merino rams, especially from the Bergerie Nationale in Rambouillet, during the first half of the twentieth century to improve wool quality, after which the exportation of Rambouillet rams largely ceased. Like the Gentile di Puglia, the Sopravissana followed seasonal transhumance routes from Lazio to Umbria and Marche along ancient Roman “consolari” roads. Although both breeds had a triple-purpose aptitude, they were mainly raised for wool. The wool market crisis in the late nineteenth century severely impacted these breeds. This crisis was caused by urbanization (leading to rural abandonment), the rise of synthetic fibers (reducing wool’s commercial value), and the increasing selection for specialized meat or dairy breeds. At the same time, restrictions on pastoral movement hindered transhumance (Panella et al. 2006). As a result, breeders were forced to repurpose these populations. Despite the good quality of their milk, low yield and poor milking ease, combined with the spread of more productive dairy breeds (e.g., Sarda and Massese), led to a shift toward meat production. This goal was pursued through intense and often indiscriminate crossbreeding, involving both Italian and foreign meat breeds—such as Württemberger (Germany), Rambouillet, Île-de-France, and Berrichon du Cher (France). Alongside population declines (further aggravated by regulations requiring breeders to cover wool disposal costs (Reg. EC 1069/2009), this led the Gentile di Puglia and Sopravissana to the brink of extinction. Indeed, by the 1980s, surveys revealed that over 80% of Gentile di Puglia and over half of Sopravissana flocks were crossbred to improve production. Consequently, a recovery project was initiated to (i) re-establish and conserve the original purebred populations, through stricter breed standards, classification into conservation programs, and molecular testing for breed verification; and (ii) improve selected crossbred lines to develop new meat breeds, namely, the Merinizzata Italiana and the Trimeticcia di Segezia (Panella et al. 2006; Sarti 2019). According to demographic data, both breeds experienced an increase in the number of registered farms and animals during the second decade of this century, but in recent years, while the number of farms has remained stable, the number of animals has declined, highlighting the need for ongoing monitoring of these populations.

Another important insight was gained by introducing the breeds of foreign origin recognized in Italian herd books into the dataset, which allowed us to better characterize their influence on Italian populations. Notably, on average, the introgression of foreign breeds into Italian sheep appeared less pronounced in the Biovita dataset compared to the modern dataset—with the exception of the Istriana breed. However, it is important to note that some of the breeds currently showing the highest levels of introgression were not included in the historical Biovita dataset. For instance, the Pecora Ciuta exhibits strong introgression from both the Île-de-France and Suffolk breeds. However, given that other analyses also indicate extensive introgression from the Merinizzata Italiana, this could contribute to the ancestral proportion derived from the Île-de-France, as better explained in the following section. The Pecora Ciuta, whose name refers to its small size, was traditionally reared in the harsh mountainous areas of Valtellina and Alto Lario. Due to pasture reduction, abandonment of mountain areas, and the introduction of larger breeds—such as Bergamasca and Merino—which were used to crossbreed the Ciuta, the population nearly disappeared. In fact, it was considered extinct as of 2001. However, the breed was later recovered starting from a small group of individuals whose morphology matched the historical descriptions of the Ciuta, and in the last 12 years the number of its registrations has progressively increased (Bigi and Zanon 2020).

The case of the Merinizzata Italiana also highlights the importance of the composition of the dataset used for analyses such as Admixture. Indeed, in the dataset including only Italian breeds, the Merinizzata Italiana formed a distinct cluster. However, when foreign breeds were included, strong introgression from the French Île-de-France and, to a lesser extent, Berrichon du Cher became evident, along with a significant contribution from the Sopravissana. This result is consistent with earlier studies on Merino and Merino-derived sheep worldwide. In Ciani et al. (2015), for example, where only the Rambouillet was included among the French breeds, the Merinizzata Italiana appeared distinct from both French and Italian Merinos. By contrast, in Ceccobelli et al. (2023), where Île-de-France and Berrichon du Cher were included, the Merinizzata Italiana clustered more closely with the French Merino-derived breeds, indicating the predominance of their genetic influence. As previously mentioned, the Merinizzata Italiana was developed in the late 1980s from crossbred individuals of Gentile di Puglia and Sopravissana origin. Particular care was taken to select animals crossed with German Merinos, in order to avoid excessive similarity to the Île-de-France, which, despite being widely used in southern Italy at the time, was poorly adapted to local production systems. These selected individuals were subjected to breeding programs aimed at standardizing morphology and improving meat production traits. As a result of these efforts, the Merinizzata Italiana was officially recognized in 1997 (Sarti 2019) and has since become one of the most widespread meat sheep breeds in Italy, despite the negative trend in registrations observed over the last 15 years. Despite clear indications to halt crossbreeding with foreign breeds and to prioritize founders with limited Île-de-France ancestry (Sarti 2019), our findings indicate that Île-de-France remains the predominant genomic ancestry in the modern Merinizzata Italiana. To preserve the breed's original genetic identity—and possibly its environmental adaptation—morphological monitoring by breed experts should be coupled with molecular analyses.

The absence of a Merinizzata Italiana–specific cluster in the admixture analysis complicated the interpretation of the introgression observed in modern Gentile di Puglia individuals, described above. To determine whether this introgression stemmed from the Merinizzata Italiana or directly from foreign breeds such as Île-de-France, we conducted a focused analysis using a subset of breeds that appeared significantly in the background of Gentile di Puglia. This subset included Île-de-France, Berrichon du Cher, and Rambouillet, as these breeds have been reported on farms currently raising Gentile di Puglia. The results clearly indicated that introgression originated primarily from the Merinizzata Italiana, rather than directly from French breeds. To explore this further, we applied local ancestry inference to identify which genomic regions of the current Gentile di Puglia carry signatures of Merinizzata Italiana introgression, and which retain ancestry from the original Gentile di Puglia. We also performed a similar analysis for the Nera di Arbus, comparing modern individuals to those in the Biovita dataset and to the Sarda, in order to assess the extent and distribution of introgression in this breed.

In addition, we compared individuals of the same breed sampled 20 years apart to identify regions that may have evolved over time under anthropogenic and/or natural selection. Our first focus was on regions related to production traits. Interestingly, we did not observe a clear separation of genes or QTLs linked to different production traits that could reflect the specific breeding purposes of the various sheep breeds. This may be due to the fact that most breeds were originally selected for triple purposes (milk, meat, and wool) and only more recently, after the wool market crisis, directed toward more specialized production. However, for most breeds, there is still no strong selection for a single production trait, as many that were originally maintained for small-scale, family-based farming have largely retained that traditional purpose.

Several genes with high Fst values—or located in regions showing peak Fst values—were associated with meat production in multiple breeds. These included genes related to body weight, growth, and conformation (*WWC1*, *ZNF385B*, *OSBPL3*, *CAMK2B*, *DDP6*) (Buzanskas et al. 2014; Santana et al. 2015; Zhao et al. 2022b; Zhan et al. 2022; Li et al. 2024; Sheet et al. 2024; Chen et al. 2025b; Liu et al. 2025b), meat quality (*HSPG2*, *RUNX1*, *GADD45A*) (Sadkowski et al. 2014; Deng et al. 2018; Jing et al. 2023; Zhang et al. 2025a), or both (*KLF15*, *CTNNA3*, *CACNA2D1*) (Yuan and Xu 2011; Qian et al. 2015; Guo et al. 2018a; Halli et al. 2022; Zhao et al. 2022a; Raza et al. 2022; Yu et al. 2023; Zhai et al. 2023; Li et al. 2024; Pinto et al. 2025). In addition, we identified several enriched QTLs associated with body measurements, weight, and muscle density—traits important not only for meat production but also for resilience during long transhumance—as well as many linked to morphological features such as ear, tail, and horn shape, which are likely under selection because they serve as distinctive identifiers of each breed. Moreover, LAI showed that regions specific to the Gentile di Puglia are also enriched for genes involved in fat metabolism, including *LPIN1* and *SLC4A7* (Martins et al. 2021; Jin et al. 2022; Hosseini et al. 2023). Meanwhile, introgressed regions include QTLs and genes related to meat quality, growth, and feed efficiency—such as *ALDH1A1*, *DLG1*, and *SLC16A7*—which is consistent with the current meat-oriented breeding objectives of both breeds (Seabury et al. 2017; Gagaoua et al. 2018; Abousoliman et al. 2021).

Unexpectedly, genes and enriched QTLs under selection over time that were also associated with milk production and quality were relatively few in all breeds, and included *ART3*, *CACNA2D1*, *CTNNA3*, *DPP6*, *PTK2*, *ZMIZ1*, *ZNF385D*, *SLC15A5*, and *ROBO2* (Wang et al. 2013; Gao et al. 2017; Jiang et al. 2019; Silva et al. 2020; Dong et al. 2021; Pedrosa et al. 2021; Mohammadi et al. 2022; Ristanic et al. 2024; Daldaban et al. 2024; Rahman et al. 2025; Li et al. 2025). Some considerations can be made for both the Nera di Arbus and the Gentile di Puglia, as introgression may play a role in shaping their milk-related traits. Indeed, the Sarda is known for its higher milk yield compared to Nera di Arbus, whose milk instead typically displays higher fat and protein content. Consistently, introgressed regions in the Nera di Arbus often contain QTLs associated with milk lactose percentage, while regions still reflecting the ancestral background include QTLs related to milk protein content. Although not primarily selected for dairy, the Gentile di Puglia also contributes to traditional cheese production—most notably the PDO *Canestrato Pugliese*, made from its milk—and some Merinizzata Italiana-introgressed regions carry QTLs associated with milk protein and fat content. Taken together, these patterns suggest that introgression may affect milk characteristics in both breeds: in the Nera di Arbus, Sarda introgression could contribute to increased yield but possibly at the expense of traditional quality traits, while in the Gentile di Puglia, Merinizzata Italiana introgression may alter the typical milk composition and, consequently, the characteristics of its dairy products.

In Fst temporal comparison, a number of genes were also related to fiber production, such as *PADI2*, *ROBO2*, *RPS6KC1*, *LAMC2*, *KIF16B*, and *ZNF385D* (Wang et al. 2014; Jin et al. 2020; Tian et al. 2021, 2025a; Lu et al. 2024; Zhu et al. 2024). QTLs linked to wool quality and quantity were enriched in some breeds, particularly in Laticauda, despite this breed not being selected for wool production. However, more considerations can be made for the LAI analyses. Indeed, as previously mentioned, the Nera di Arbus sheep differs from the Sarda not only in clearly observable morphological traits—such as its black fleece and the presence of horns—but also in its ancestral lineage and, more importantly, in its traditional aptitude for wool production, which has historically supported the creation of local handcrafted textiles. Several genes central to this function, such as *EGFR* and *CTNNB1* (Zhang et al. 2025b; ZinAlabidin et al. 2025), were detected in regions still associated with the older, more ancestral Nera di Arbus background, likely reflecting the retention of ancestral genomic components. Nonetheless, a significant portion of genes involved in fiber development—including several keratin genes, along with *RERE* and *STAT3*—were found in highly introgressed regions (Zhao et al. 2021; Arzik et al. 2023). This suggests that gene flow from Sarda, a non-wool breed, may have affected the original fiber-related genomic architecture of the Nera di Arbus. In the context of breed conservation, where the maintenance of unique ancestral traits is a central goal, such introgression could compromise local wool quality and thus hinder the valorization of traditional products and reduce the distinctiveness of the breed. Similarly, with regard to the Gentile di Puglia, we identified genomic regions associated with hair follicle development and wool type differentiation within ancestral regions, reflecting its original wool-oriented breeding purpose. While wool is no longer the main productive focus, introgressed regions from the Merinizzata Italiana that include QTLs linked to fleece yield and *VDR*, a gene involved in follicle development (Zhao et al. 2021), could potentially alter the typical fleece traits that characterize the Gentile di Puglia.

Another pivotal aspect of livestock breeding, beyond production itself, is reproduction—both to obtain lambs for meat production, to ensure lactating ewes, and to provide replacements for future generations. It is therefore not surprising that many regions under selection over time harbor genes involved in fertility and litter size (*CHRNA2*, *ENPP3*, *RABL3*, *CDH18*, *HSPG2*, *RUNX1*, *ZMIZ1*, *NCOA1*, *PTK2*) (Zachut et al. 2016; Liu et al. 2017; Sundaram et al. 2018; Gao et al. 2018; Yuan et al. 2019; Shabbir et al. 2021; Pacheco et al. 2022; Ahmad et al. 2023; Wang et al. 2024c; Ogunbawo et al. 2025; Xiao et al. 2025) and, in some dairy breeds, QTLs for reproductive seasonality or lambing ease. In both Nera di Arbus and Gentile di Puglia, fertility-related genes and QTLs were found both in introgressed and ancestral regions. This overlap reflects the complexity of the breed’s genomic landscape and may also point to heterosis-like effects, with introgression increasing genetic diversity at fertility loci and potentially influencing fertility-related traits.

Health-related factors represent a major determinant of livestock productivity, reproductive efficiency, and welfare. Consistently, immunity-related genes and QTLs were among the most frequently associated with SNPs showing high Fst values, and immunity-related GO terms were significantly enriched in the Delle Langhe and Laticauda breeds. These included genes and QTLs related to somatic cell count and mastitis (*CACNA2D1*, *SEMA5A*, *NEGR1*, *PTK2B*, *CTNNA3*) (Sugimoto et al. 2006; Deb et al. 2013; Yang et al. 2019; Mohammadi et al. 2022; Wagner et al. 2023), gastrointestinal parasites (*PTK2B*) (Thorne et al. 2023), and various viral or bacterial diseases. Among these, specific examples include: genes associated with respiratory diseases (*PADI2*) (Cao et al. 2020) and QTLs for paratuberculosis (in the Bergamasca) and pleurisy (in Comisana); *CTNNA3* gene, found in Altamurana and associated with brucellosis (Li et al. 2021b); a QTL in Fabrianese and *HSPG2* gene in Appenninica for footrot (Gaspar et al. 2024), which represents a significant cause of lameness in sheep flocks, leading to economic losses due to reduced wool production, poor fertility, and decreased growth rates; *GADD45A*, found in Laticauda, for *Peste des petits ruminants* virus (Ding et al. 2025); and both QTLs and *CXCL9* gene for Visna-Maedi (Shi et al. 2024). Notably, among the highly differentiated genes in Valle del Belice, we also identified *CXCL10*, which encodes chemokines involved in various infections and infestations (Gangur et al. 2002; Andronicos et al. 2010; Estrada-Reyes et al. 2022; Pooley et al. 2023; Luo et al. 2024; Shi et al. 2024). Moreover, when we analyzed introgression in the Nera di Arbus, we found that some of the PNAbv regions harbor QTLs and genes (e.g., *C9*, *FYB*, and *MYD88*) related to somatic cell count and mastitis resistance, traits essential for udder health (Banos et al. 2017; El-Sayed et al. 2025). Other genes involved in immune response are *MYD88*, a candidate gene for resistance to small ruminant lentiviruses, and *FOCF1*, linked to pneumonia resistance (Huang et al. 2025). In contrast, introgressed regions contained *STAT5A* and *CSF3*, both associated with mastitis resistance in cattle (Usman et al. 2014; Stella et al. 2018), and genes (*CX3CR1* and *CSF3*) and QTLs involved in immune response and parasite resistance (Estrada-Reyes et al. 2022).

Alongside selection for productive and fitness-related traits, livestock breeds, especially local populations raised in extensive systems, are also shaped by selective pressures that drive adaptation to their specific environments. This can explain why we found several non-introgressed regions associated to environmental adaptation, both in the Nera di Arbus—such as *CBFA2T3* and *DNAJA2*, associated with heat tolerance in cattle (Li et al. 2020a; Ben-Jemaa et al. 2020; Habimana et al. 2023), and *EGFR*, linked to environmental and altitude adaptation in sheep and Tibetan goats (Gonzalez-Bulnes et al. 2022; Liu et al. 2025a)—and Gentile di Puglia—in particular *SOD2*, involved in oxidative stress protection (Hadfield et al. 2018; Shi et al. 2020; Austin et al. 2024; Wang et al. 2024b). However, the presence of genes such as *SLC16A7* and *KHDRBS2*, linked to responses to environmental stressors (Peng et al. 2024; Liu et al. 2025a), within Merinizzata Italiana-introgressed segments raise concerns about the potential erosion of locally adapted genetic variants that may be crucial for the breed’s resilience in the challenging environments of southern Italy. A substantial number of these adaptive genes were also found by Fst within-breed temporal comparison—many detected in Valle del Belice. These included associations with solar radiation (*KLF15*) (Salehian-Dehkordi et al. 2023), drought (*CACNA2D1*) (Yang et al. 2016), and heat tolerance (*C4H4orf22*) (Niu et al. 2024). However, most were linked to altitude adaptation, including *AGBL4*, *PDK4* (also relevant to energy metabolism), *PTPRD*, *SLC22A17*, *LONP1*, *ASCC3*, and *CAMK2B* (Wei et al. 2016; Basang and Zhu 2019; Wen et al. 2021; Liang et al. 2024; Zhang et al. 2024; Ahmad et al. 2024; Ayalew et al. 2024).

Given these signals, we further explored how climate change and corresponding natural selection have shaped these breeds in recent decades. Specifically, we identified candidate SNPs for environmental adaptation by analyzing their correlation with shifting environmental variables and marked breed genetic differentiation over time. The strongest signals were tied to BIO16 (precipitation during the wettest quarter), followed by aridity annual mean, BIO09 (mean temperature of the driest quarter), and BIO11 (mean temperature of the coldest quarter). Precipitation-related candidates (BIO16) were particularly abundant when comparing the current and historic Istriana populations. Noteworthy among the associated genes many have been previously associated to livestock environmental adaptation, including *CD109* and *ST3GAL*, both implicated in sheep adaptation to arid and hot climates (Aboul-Naga et al. 2022; Saadatabadi et al. 2023), and *EPAS1*, a key regulator of oxygen homeostasis under hypoxia and a recognized marker of altitude adaptation in several species (Song et al. 2016; Guo et al. 2018b). We also detected aquaporin genes and enriched GO terms related to renal water transport. Aquaporins are crucial for renal water reabsorption and urine concentration, highlighting the importance of osmoregulation in environments with scarce or unpredictable rainfall and, consequently, the adaptive significance of maintaining water balance in livestock exposed to arid or variable climates (Agarwal and Gupta 2008; Chedid et al. 2014; Kaushik et al. 2023).

The results of BIO11 correlation revealed genes linked to organ growth, osteoblast activity, and Hippo signaling. These processes are integral to body-size modulation, a classic adaptive trait shaped by climate. Indeed, according to Bergmann’s rule, animals living in hotter climates tend to be smaller, as a reduced body size favors heat dissipation (Bergmann 1847). Alternatively, populations evolved in hot but arid environments can also present large bodies, which reduces metabolic rate and endogenous heat production, but long and thin appendages, which increase surface area and facilitate heat loss (Berihulay et al. 2019). This morphological adaptation aligns with our observation of enriched pathways related to chondrocyte proliferation for BIO09, suggesting an underlying genetic basis for limb elongation in these populations (Serrat et al. 2008). Moreover, the Hippo pathway, in particular, orchestrates the so-called “pace of life”, influencing not only growth, but also sexual maturation timing, lifespan, and metabolism, thus representing a molecular bridge between environmental stimuli and developmental responses (Ahi et al. 2025).

Several ontologies significant for BIO11 are involved neurological development—including glycinergic synapse regulation—and behavioral modulation. Behavioral plasticity enables rapid responses to environmental challenges, allowing animals to modify feeding, foraging, or social behaviors quickly (Sejian et al. 2018). Glycinergic synapses, serving as key inhibitory transmitters in spinal and brainstem circuits, play a critical role in shaping motor and sensory pathways (e.g. respiration, vision, audition, pain processing), thereby also influencing behavioral output (Avila et al. 2013).

For BIO09, we also observed enrichment in the regulation of diadenosine triphosphate (Ap₃A) catabolism. Ap₃A belongs to the “alarmones”, stress-responsive signaling molecules whose levels rise swiftly with external stress to regulate gene expression, modulate ATP-binding enzyme activity, and stimulate repair and antioxidant pathways (Götz et al. 2019).

Aridity-related ontologies also revealed negative regulation of DNA transcription, suggesting a strategy of resource conservation under environmental strain: stress-driven epigenetic remodeling in immune cells is one documented mechanism resulting in broad transcriptional suppression and a shift to post-transcriptional control (Caldwell and Li 2024).

Other genes and pathways shared across environmental variables involved immune response, metabolic and energy regulation, angiogenesis and cardiac function, circadian rhythm, and oxidative protection. Circadian regulation aligns metabolic, reproductive, and behavioral processes with daily and seasonal environmental cycles, a critical adaptation mechanism (Yerushalmi and Green 2009; Li et al. 2021a; Casey and Plaut 2022). Oxidative stress management is equally vital under thermal extremes, with copper and zinc—linked here to BIO09—being essential trace elements for antioxidant enzymes and immune function (Kleczkowski et al. 2003; Matuszczak et al. 2024). Purine metabolism, responding dynamically to oxidative and thermal stress, plays a central role in energy management (Tian et al. 2022).

Selection under aridity also affected retinol metabolism pathways. Vitamin A supports vision, immune function, epithelial integrity, and resistance to oxidative stress—particularly important where UV exposure, heat, and pathogen pressure are high (Shastak et al. 2023). Immune regulation itself is a core adaptive mechanism: environmental stressors like heat and drought are known to compromise immune responsiveness in livestock (Chedid et al. 2014). Across climatic gradients, resilient immune capability is vital for survival and sustained productivity, and with climate change expanding parasitic and pathogen threats, selecting for immune-adapted livestock is increasingly important (Ciliberti et al. 2022).

Metabolic regulation allows dynamic energy adjustment to environmental demands (Lima et al. 2022). Triglyceride synthesis and lipid mobilization are central in thermoregulation and energy storage under varying conditions (Olsen et al. 2021). For example, *FTO*, strongly associated with BIO16 in the Sambucana sheep, is crucial for fat metabolism, feed efficiency, meat quality, and tail fat deposition—a reserve for coping with harsh conditions (Chung 2014; Jevsinek Skok et al. 2016; Wang et al. 2021; Chen et al. 2024a). Variants and epigenetic modification in *FTO* have also been linked to heat and environmental variability in sheep and goats (Chen et al. 2023, 2025a; Peng et al. 2024).

Thermoregulatory demands during heat stress prompt physiological adaptations like increased respiration, heart rate, and microvascular perfusion (Renaudeau et al. 2012; Cartwright et al. 2023). Indeed, we found GO terms related to cardiac function (BIO09) and microvascular development (aridity annual mean), which contribute to dissipate heat, support tissue perfusion, and preserve homeostasis under thermal and hydric stress.

In conclusion, this study provides a comprehensive analysis of the recent history and genomic evolution of Italian sheep biodiversity, revealing a multifaceted picture of both challenges and dynamics. Our results demonstrate the resilience and adaptability of these breeds, showing that despite demographic pressures, many populations maintain substantial genetic diversity and retain traits shaped by centuries of environmental adaptation. However, they also serve as a clear wake-up call regarding the survival prospects of many Italian breeds, highlighting reductions in population size, declines in genetic variability, and widespread cases of admixture. For instance, the evidence of clear introgression in some local breeds underscores the dual nature of gene flow: while it can introduce advantageous alleles that support adaptation or production, by increasing milk or meat yield, it may also alter traits defining breed identity, for example by altering the quality of typical dairy products or the production of wool used for traditional handicraft. Balancing the conservation of ancestral genomic signatures with the benefits of adaptive introgression is therefore a central challenge. In this context, breeders’ associations are pivotal: by collecting and analyzing farm-level data nationally, they support the management and conservation of genetic resources and foster farmers’ engagement in breed improvement. However, the coordinating role of breeders’ associations in safeguarding and improving populations does not appear to be fully leveraged and understood, highlighting the need for greater structured efforts and collaboration among all stakeholders in the Italian breeding system: breeders, breeders’ associations, and government institutions. Complementing these initiatives with an integrative approach that combines demographic, farm-level, and genomic data is essential to preserve genetic diversity and support the long-term sustainability of Italian sheep biodiversity.

## Supplementary Information

Below is the link to the electronic supplementary material.

**Supplementary Fig. S1** Trends in yearly animal and farm registrations per breed from 2010 to 2024. (PDF 26325 kb)

**Supplementary Fig. S2** Admixture analysis for a number of clusters (K) ranging from 2 to 35. (PNG 499 kb)

**Supplementary Fig. S3** Genetic diversity of Italian sheep breeds. a) Boxplot of individual inbreeding coefficients based on runs of homozygosity (F_ROH_). Each point corresponds to one individual. Boxplots show the median (line), interquartile range (IQR, box), whiskers extending up to 1.5 × IQR, and points beyond whiskers representing outliers. b) Barplot of mean F_ROH_ per breed. Colors indicate the contribution of ROH of different length classes. Breeds are ordered by total mean F_ROH_. (PNG 1156 kb)

**Supplementary Fig. S4** Admixture analysis for a number of clusters (K) ranging from 20 to 30, including recent and older (codes including ‘bv’) samples. (PNG 383 kb)

**Supplementary Fig. S5** Local ancestry inference with Gentile di Puglia (GDP) as the target population, older GDP samples (GDPbv) as the background, and Merinizzata Italiana (MER) as the candidate source of introgression. (PDF 584 kb)

**Supplementary Fig. S6** Local ancestry inference with Nera di Arbus (PNA) as the target population, older PNA samples (PNAbv) as the background, and Sarda (SAR) as the candidate source of introgression. (PDF 589 kb)

**Supplementary Tables** Supplementary Table S1 Dataset composition for the main analyses performed in the present study.Supplementary Table S‎2 Description of the climatic variables used for correlation analysis of Fst values with climatic change in each population's breeding range.Supplementary Table S‎3 SNPs associated with the highest 1% |Fst| values in each breed comparison (older vs. recent samples of the same breed).Supplementary Table S4 Genes associated with the highly differentiated SNPs identified in each breed comparison (older vs. recent samples of the same breed) through Fst (see Table S3).Supplementary Table S5 GO terms associated with the highly differentiated genes identified in each breed comparison (older vs. recent samples of the same breed) through Fst (see Table S4).Supplementary Table S‎6 SNPs associated with the highest 1% |Fst| values in at least one breed comparison and significantly correlated to at least one environmental variable change.Supplementary Table S7 Genes associated with the SNPs identified in correlation analysis of Fst values with climate change (see Table S6).Supplementary Table S8 GO terms associated with the highly identified in correlation analysis of Fst values with climate change (see Table S7).Supplementary Table S9 SNPs in the 99th percentile of most introgressed regions of older Gentile di Puglia population (GDPbv) or Merinizzata Italiana (MER) in Gentile di Puglia (GDP) or of older Nera di Arbus (PNAbv) or Sarda (SAR) in Nera di Arbus (PNA) according to local ancestry inference (LAI) analysis.Supplementary Table S10 Genes associated with the most introgressed regions identified in LAI analysis (see Table S9). (XLSX 23033 kb)

## Data Availability

The data that support the findings of this study are available from Asso.Na.Pa. but restrictions apply to the availability of these data, which were used under licence for the current study, and so are not publicly available. For further information, please write to S.G. at direzione@assonapa.it.
