# Supplementary material for: Genomic insights into the recent evolution and biodiversity of Italian sheep breeds
Source: Mamm Genome. 2025 Nov 22;37(1):5. doi: 10.1007/s00335-025-10170-8 (PMC12640353; doi:10.1007/s00335-025-10170-8)
Supplement: Supplementary file 1 — Supplementary Fig. S1 Trends in yearly animal and farm registrations per breed from 2010 to 2024. (PDF 26325 kb) [file 335_2025_10170_MOESM1_ESM.pdf]

ALPAGOTA

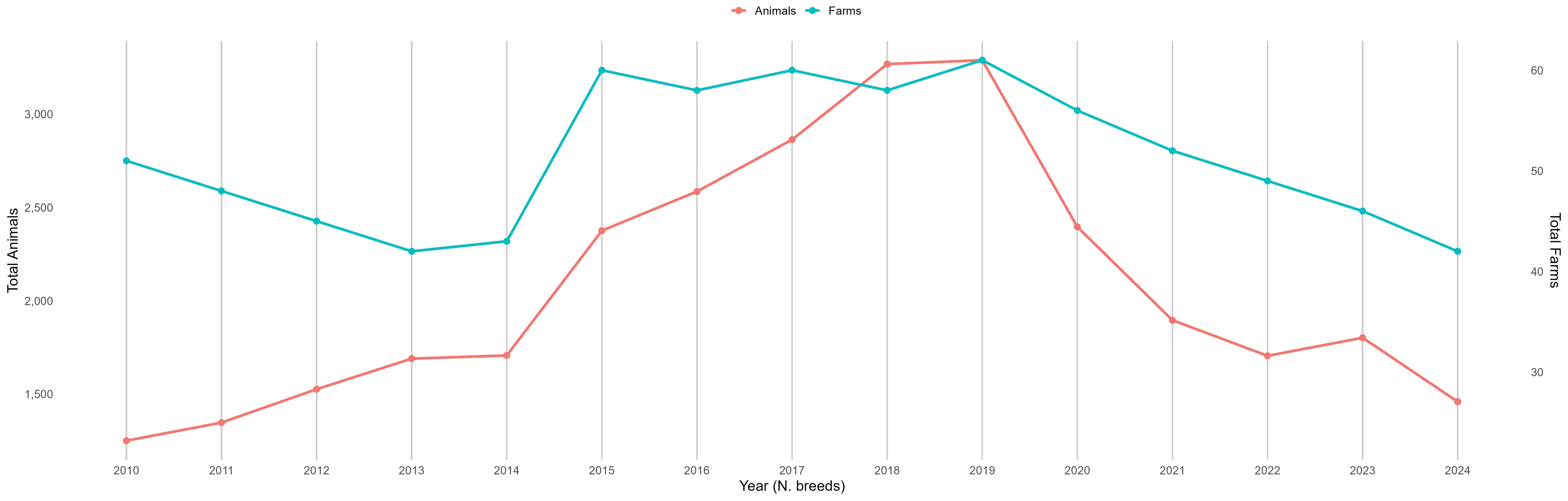

Animals
Farms
3,000
2,500
2,000
1,500
Total Animals
60
50
40
30
Total Farms
2010
2011
2012
2013
2014
2015
2016
2017
2018
2019
2020
2021
2022
2023
2024
Year (N. breeds)

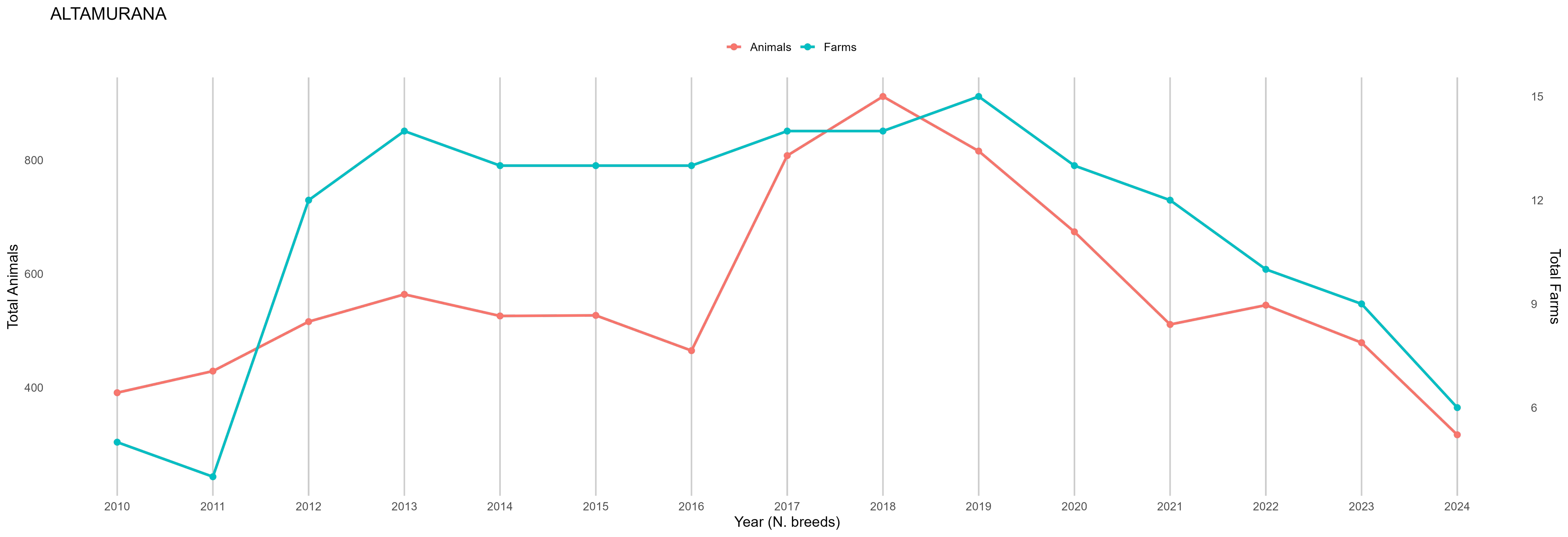
ALTAMURANA
Animals
Farms
Total Animals
Total Farms
Year (N. breeds)
400
600
800
6
9
12
15
2010
2011
2012
2013
2014
2015
2016
2017
2018
2019
2020
2021
2022
2023
2024

APPENNINICA

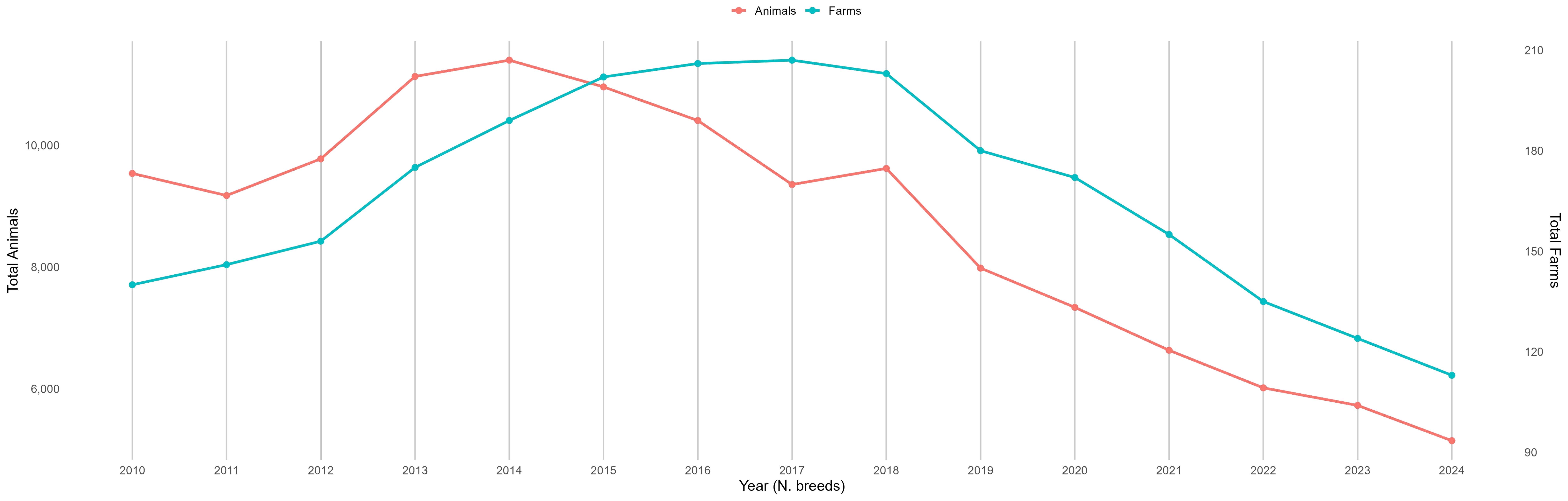

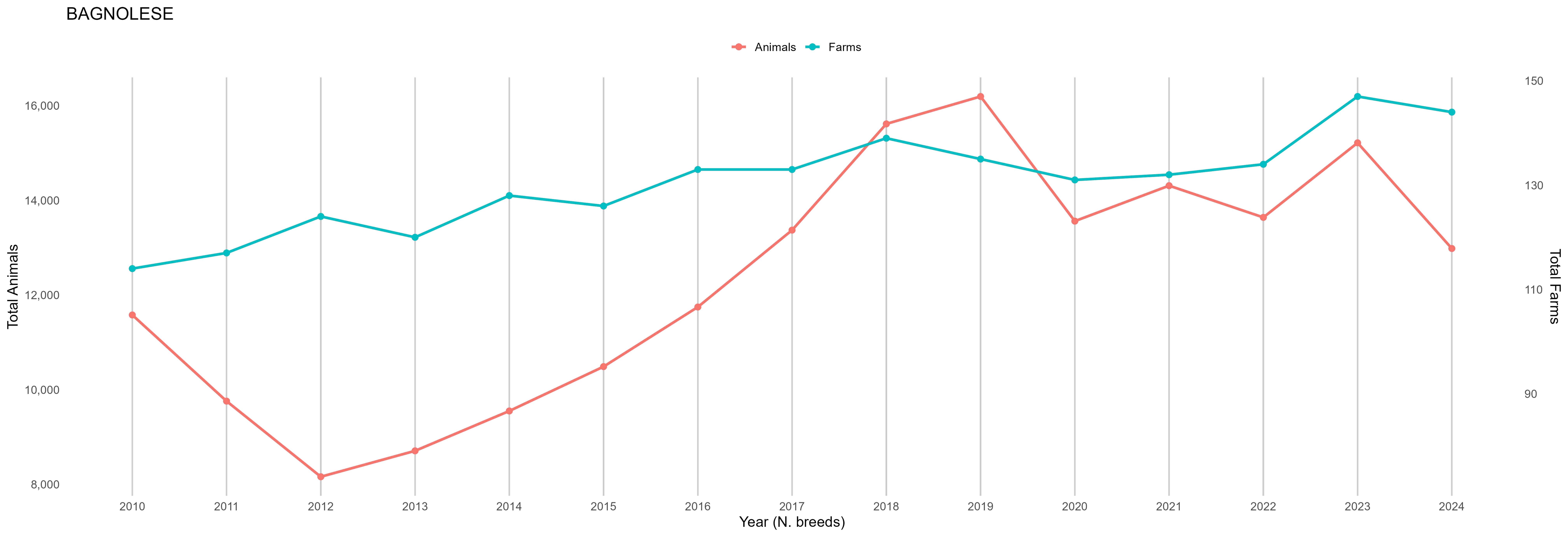
BAGNOLESE
Animals
Farms
Total Animals
16,000
14,000
12,000
10,000
8,000
Total Farms
150
130
110
90
2010
2011
2012
2013
2014
2015
2016
2017
2018
2019
2020
2021
2022
2023
2024
Year (N. breeds)

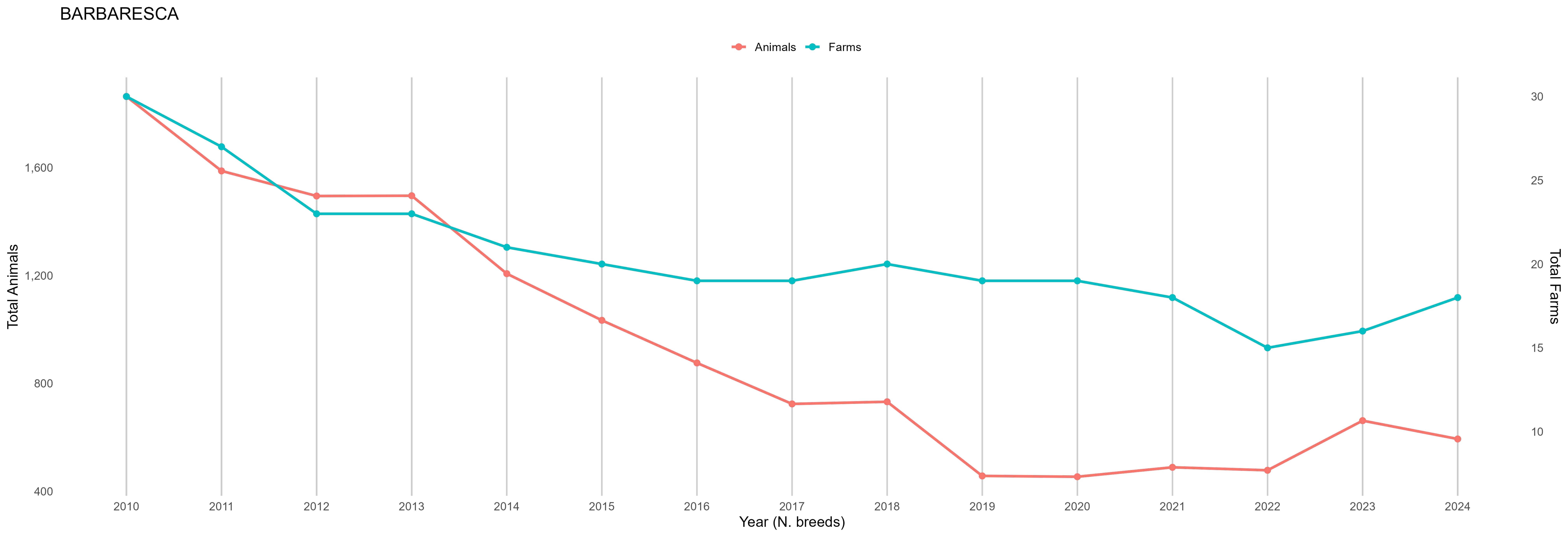

BARBARESCA
Animals
Farms
Total Animals
1,600
1,200
800
400
Total Farms
30
25
20
15
10
2010
2011
2012
2013
2014
2015
2016
2017
2018
2019
2020
2021
2022
2023
2024
Year (N. breeds)

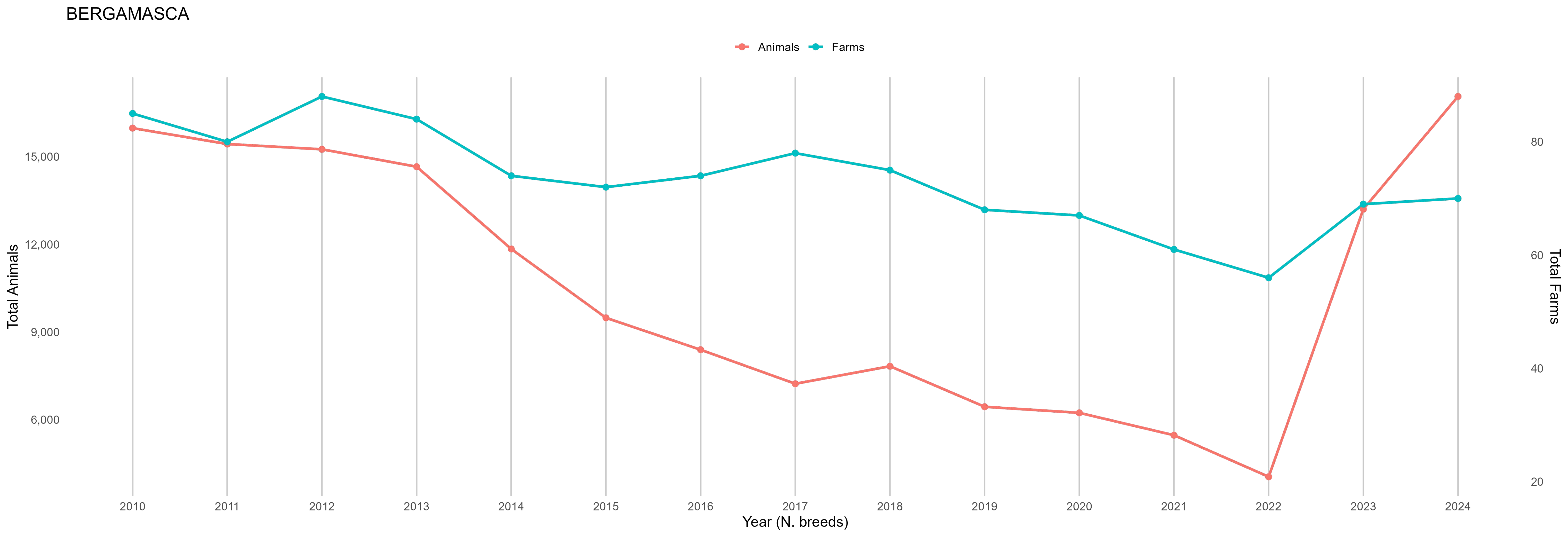

BERGAMASCA
Animals
Farms
Total Animals
15,000
12,000
9,000
6,000
Total Farms
80
60
40
20
2010
2011
2012
2013
2014
2015
2016
2017
2018
2019
2020
2021
2022
2023
2024
Year (N. breeds)

BIELLESE

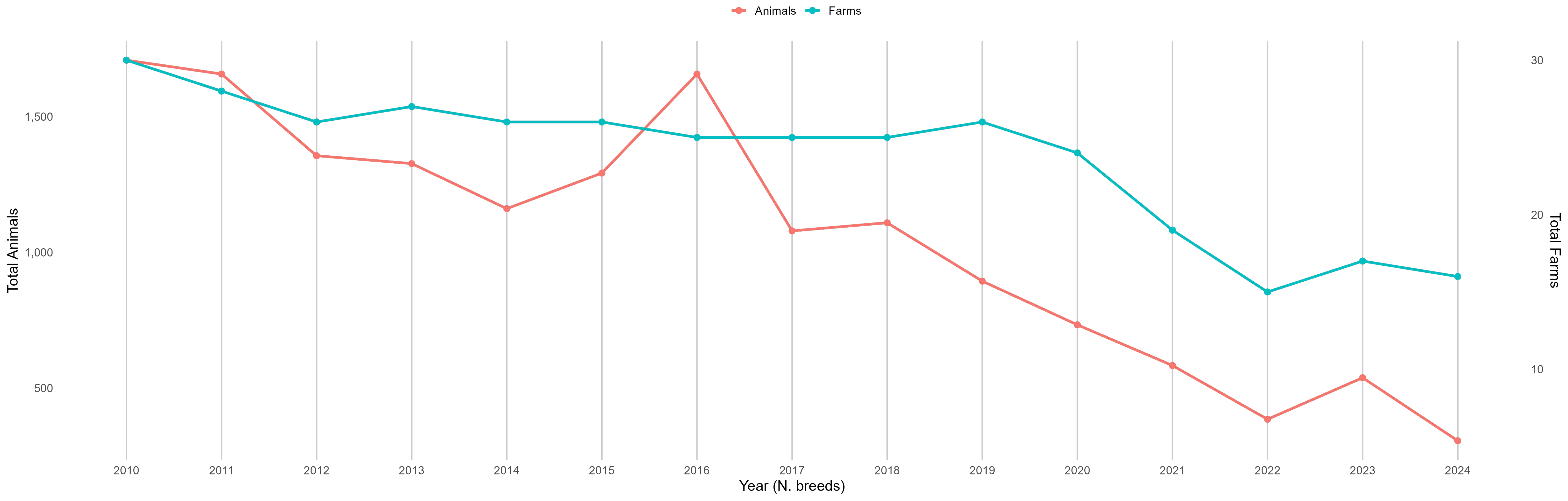

Animals
Farms
Total Animals
Total Farms
1,500
1,000
500
30
20
10
2010
2011
2012
2013
2014
2015
2016
2017
2018
2019
2020
2021
2022
2023
2024
Year (N. breeds)

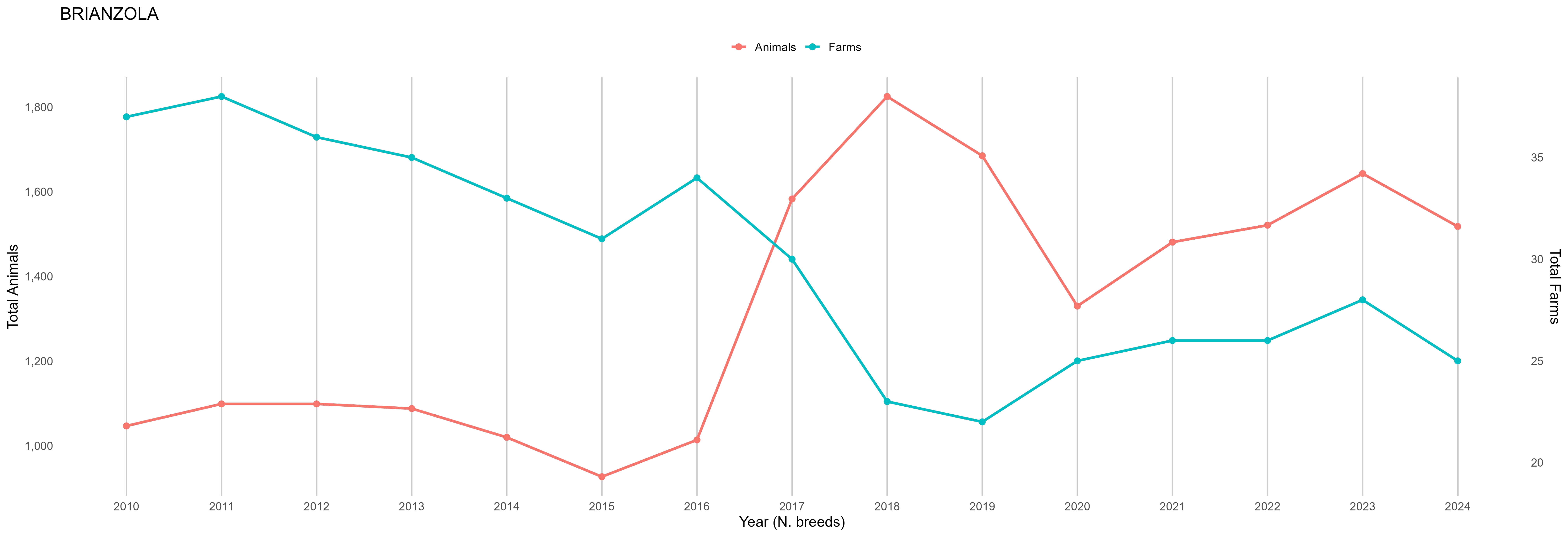

BRIANZOLA
Animals
Farms
Total Animals
1,800
1,600
1,400
1,200
1,000
Total Farms
35
30
25
20
2010
2011
2012
2013
2014
2015
2016
2017
2018
2019
2020
2021
2022
2023
2024
Year (N. breeds)

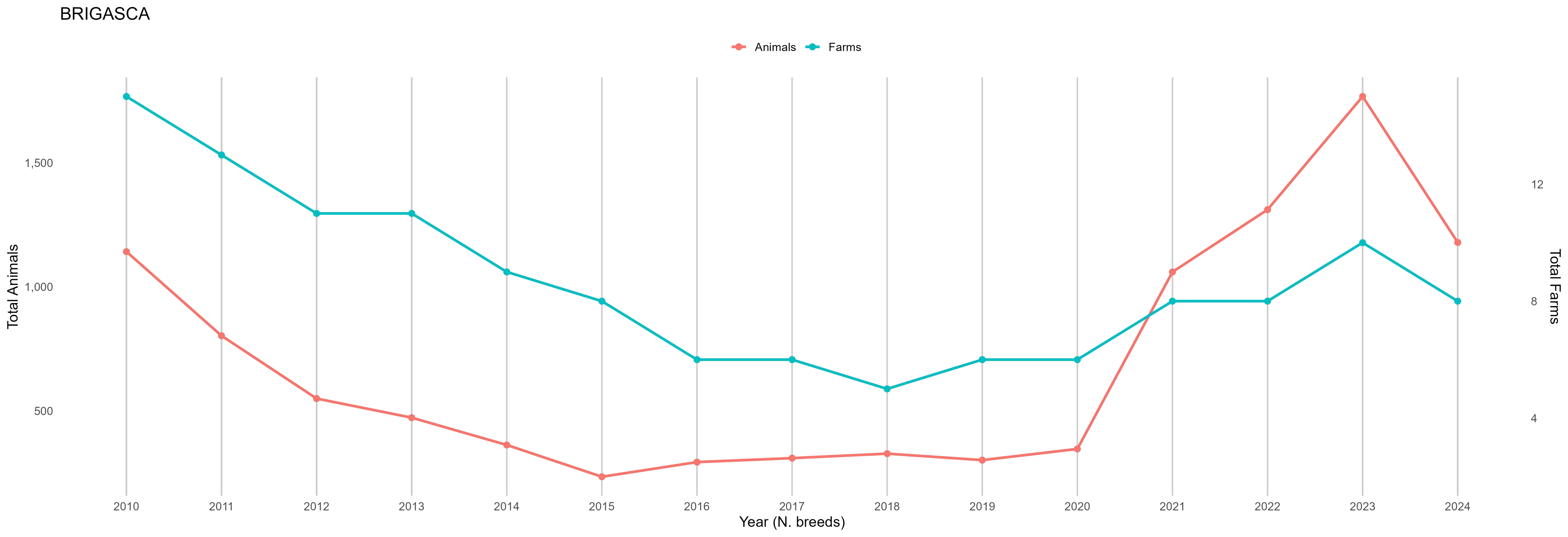
BRIGASCA
Animals
Farms
Total Animals
1,500
1,000
500
Total Farms
12
8
4
2010
2011
2012
2013
2014
2015
2016
2017
2018
2019
2020
2021
2022
2023
2024
Year (N. breeds)

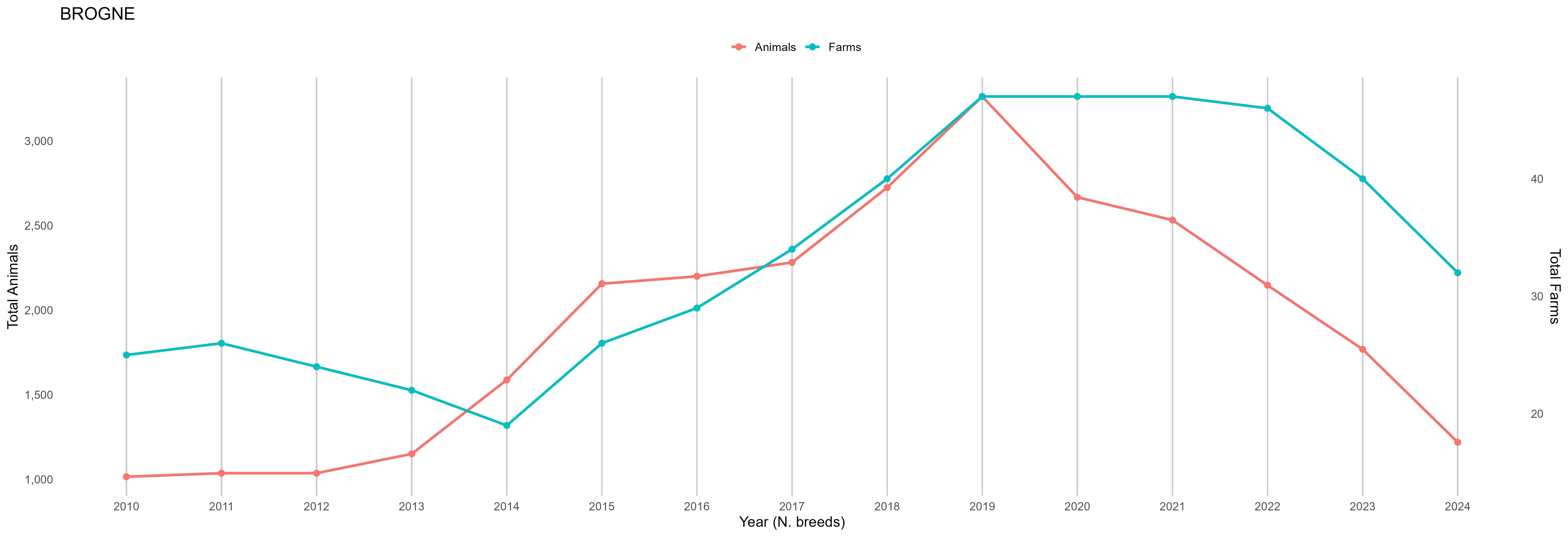

BROGNE
Animals
Farms
Total Animals
3,000
2,500
2,000
1,500
1,000
Total Farms
40
30
20
2010
2011
2012
2013
2014
2015
2016
2017
2018
2019
2020
2021
2022
2023
2024
Year (N. breeds)

COMISANA

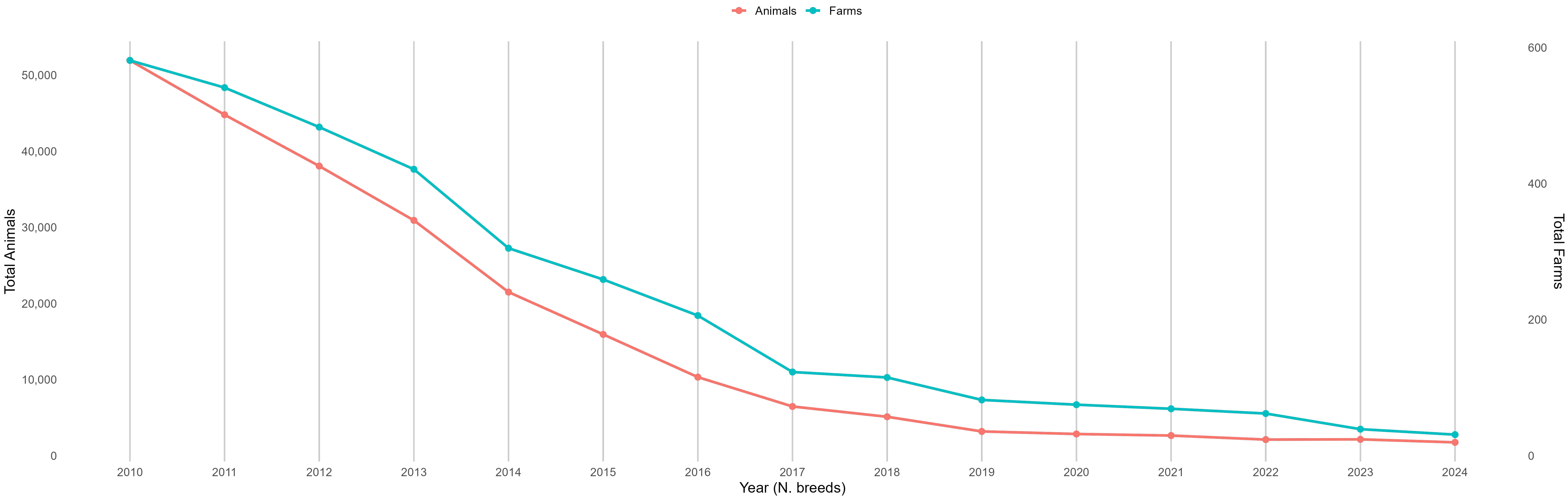

Animals
Farms
Total Animals
Total Farms
50,000
40,000
30,000
20,000
10,000
0
600
400
200
0
2010
2011
2012
2013
2014
2015
2016
2017
2018
2019
2020
2021
2022
2023
2024
Year (N. breeds)

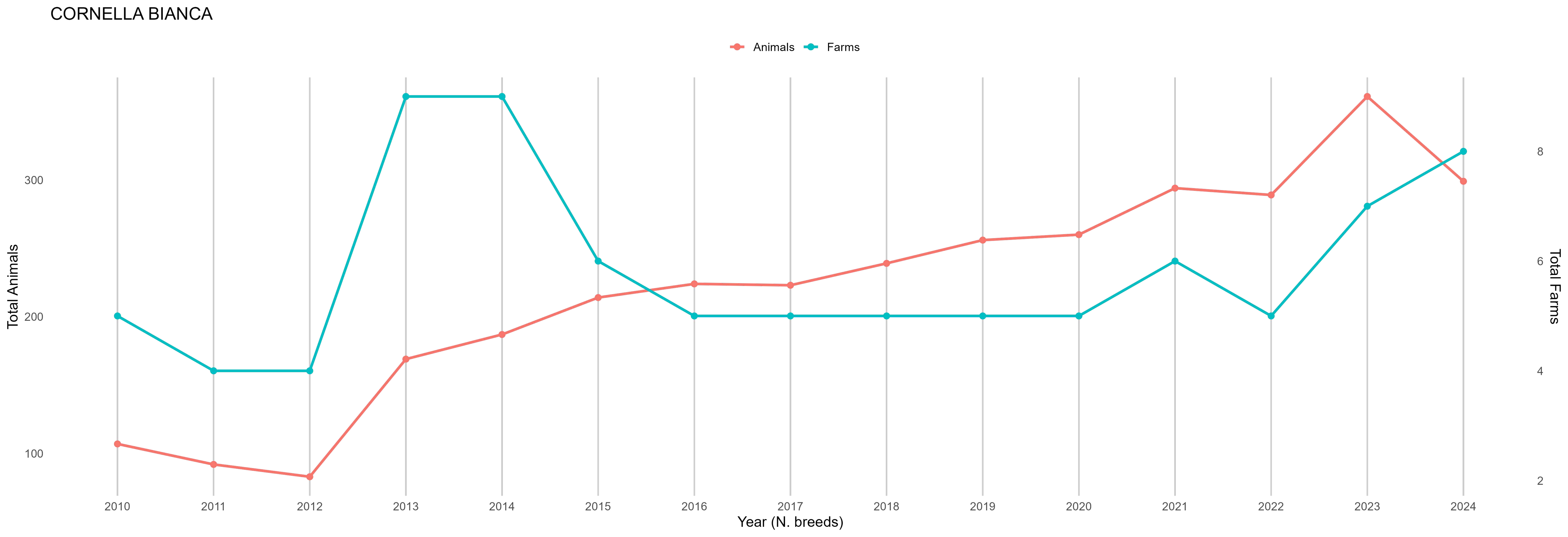
CORNELLA BIANCA
Animals
Farms
Total Animals
Total Farms
Year (N. breeds)
300
200
100
8
6
4
2
2010
2011
2012
2013
2014
2015
2016
2017
2018
2019
2020
2021
2022
2023
2024

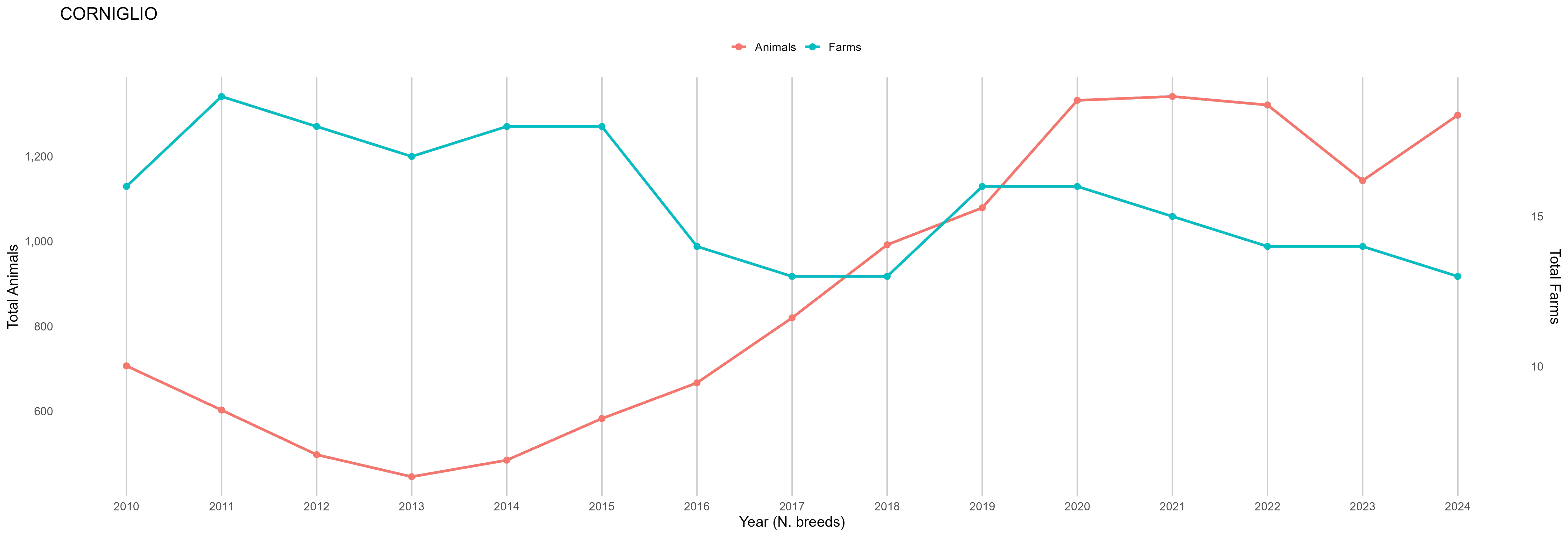

CORNIGLIO
Animals
Farms
1,200
1,000
800
600
Total Animals
15
10
Total Farms
2010
2011
2012
2013
2014
2015
2016
2017
2018
2019
2020
2021
2022
2023
2024
Year (N. breeds)

DELL AMIATA

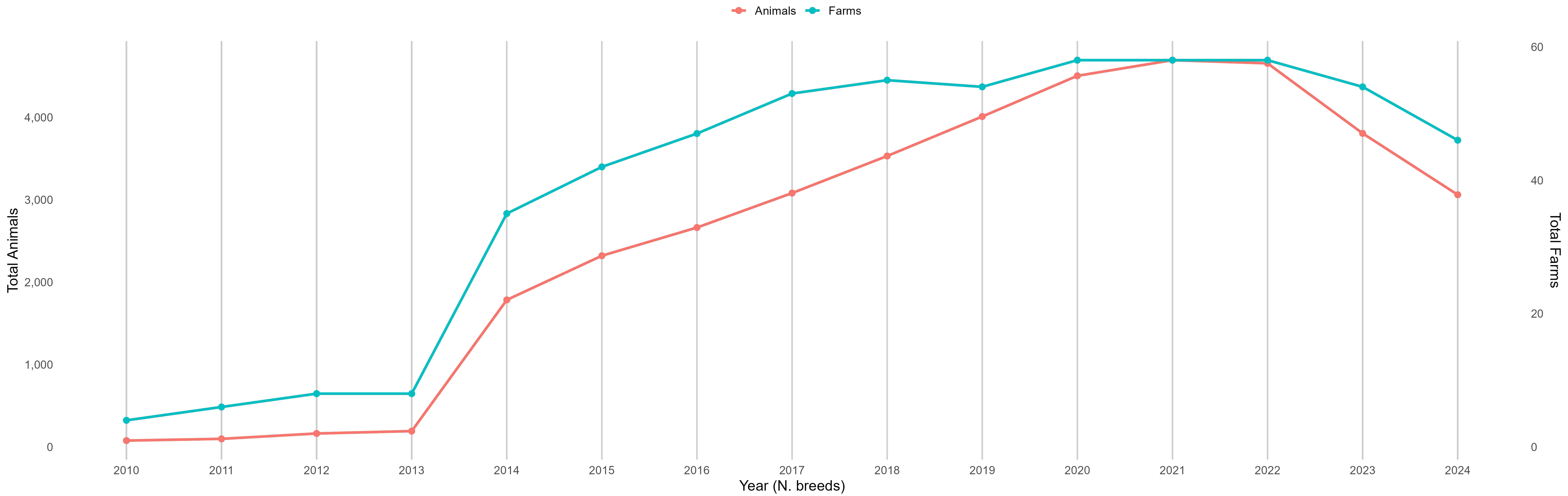

Animals
Farms
Total Animals
Total Farms
Year (N. breeds)
0
1,000
2,000
3,000
4,000
0
20
40
60
2010
2011
2012
2013
2014
2015
2016
2017
2018
2019
2020
2021
2022
2023
2024

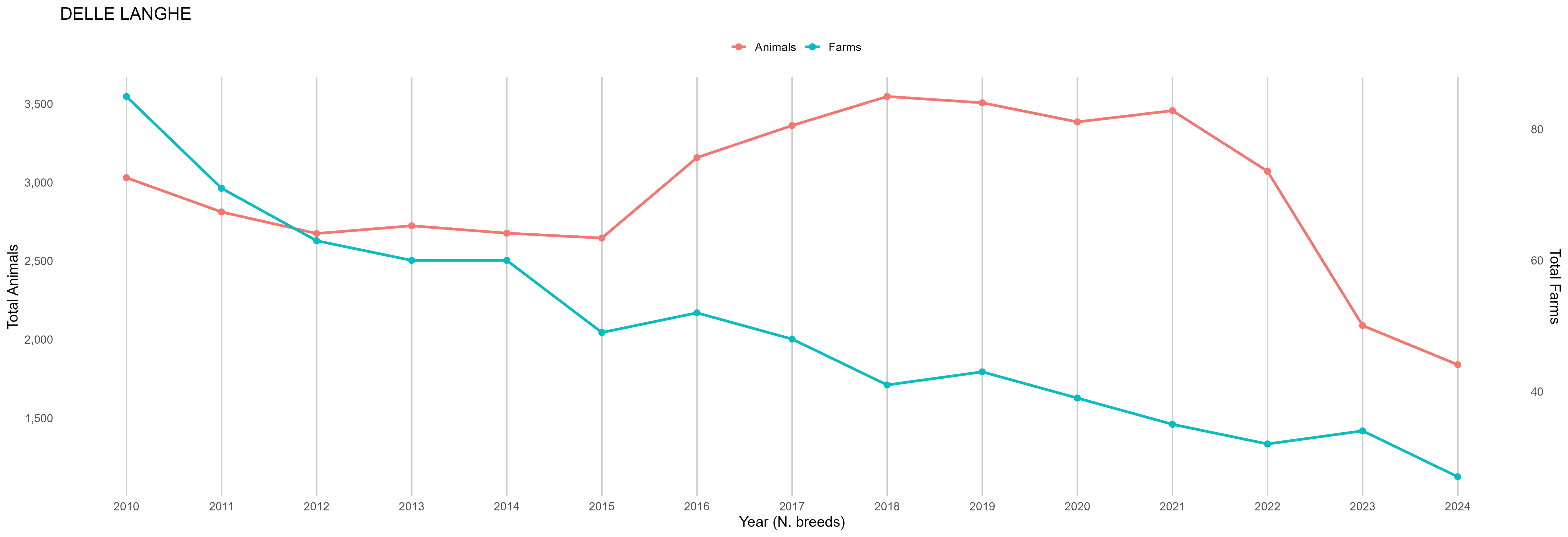

DELLE LANGHE
Animals
Farms
Total Animals
3,500
3,000
2,500
2,000
1,500
Total Farms
80
60
40
2010
2011
2012
2013
2014
2015
2016
2017
2018
2019
2020
2021
2022
2023
2024
Year (N. breeds)

DI BENEVENTO-QUADRELLA

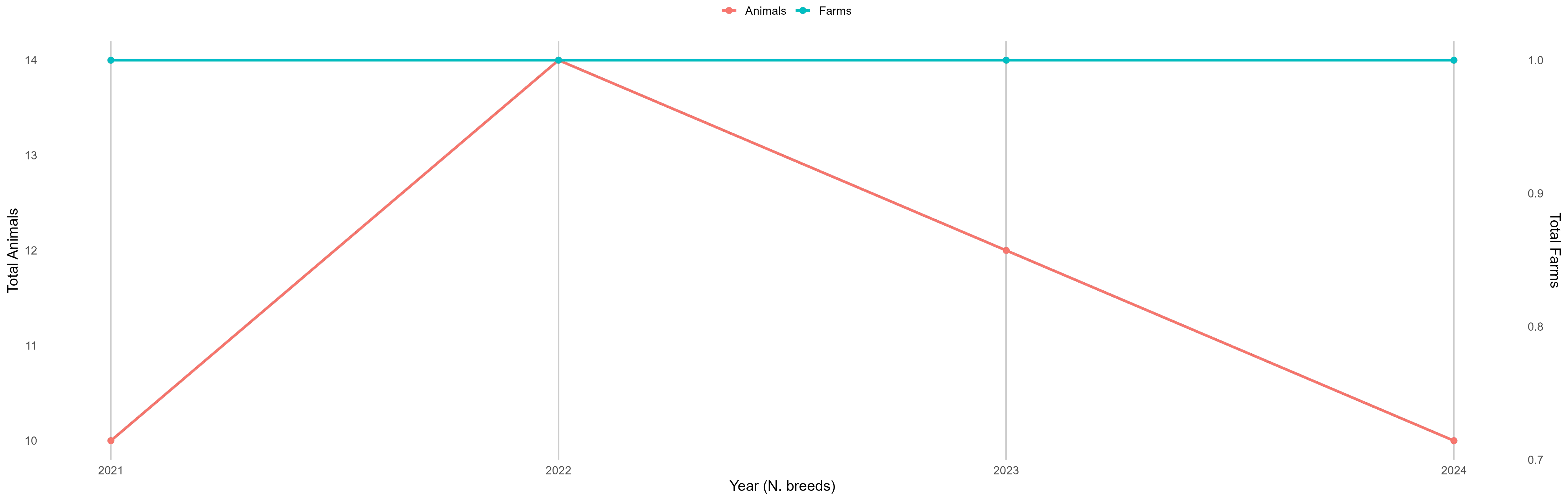

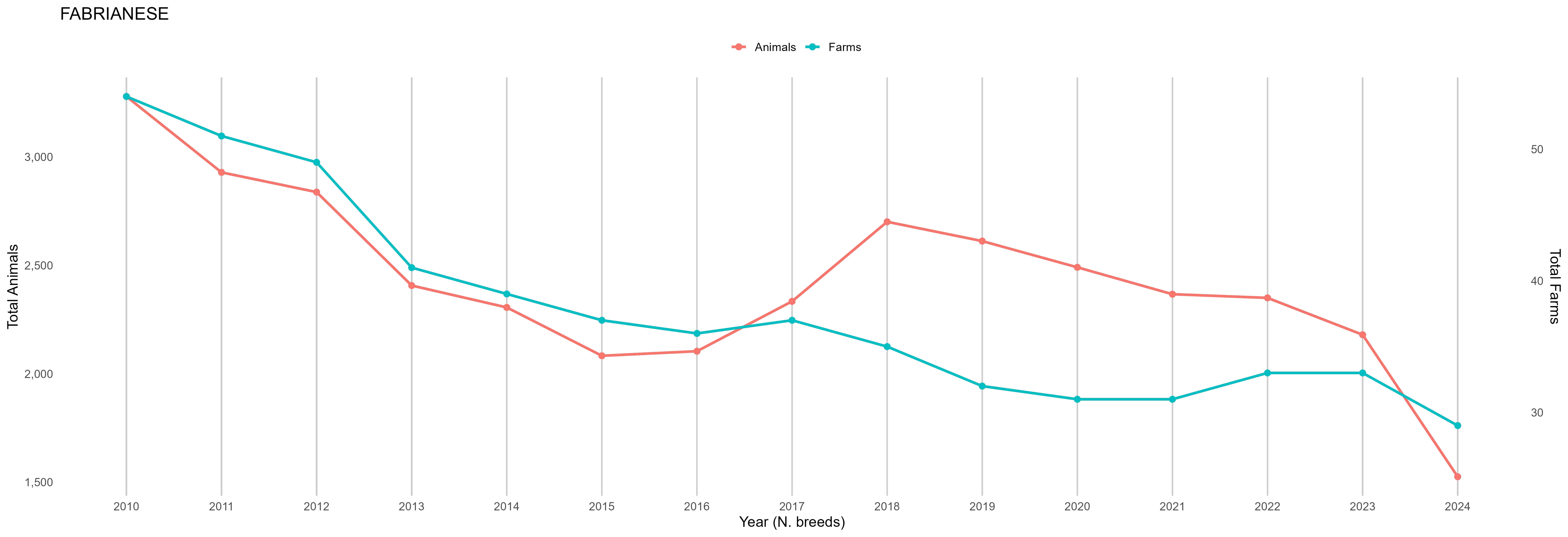
FABRIANESE
Animals
Farms
Total Animals
Total Farms
3,000
2,500
2,000
1,500
50
40
30
2010
2011
2012
2013
2014
2015
2016
2017
2018
2019
2020
2021
2022
2023
2024
Year (N. breeds)

FRABOSANA

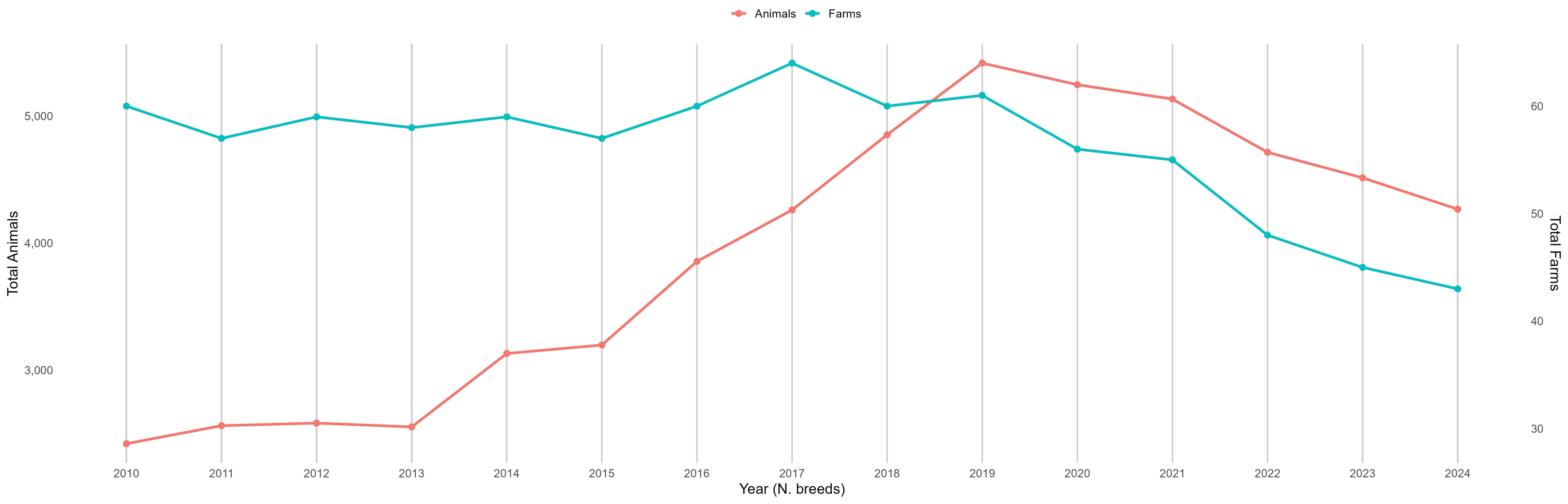
Animals
Farms
Total Animals
5,000
4,000
3,000
Total Farms
60
50
40
30
2010
2011
2012
2013
2014
2015
2016
2017
2018
2019
2020
2021
2022
2023
2024
Year (N. breeds)

GARESSINA

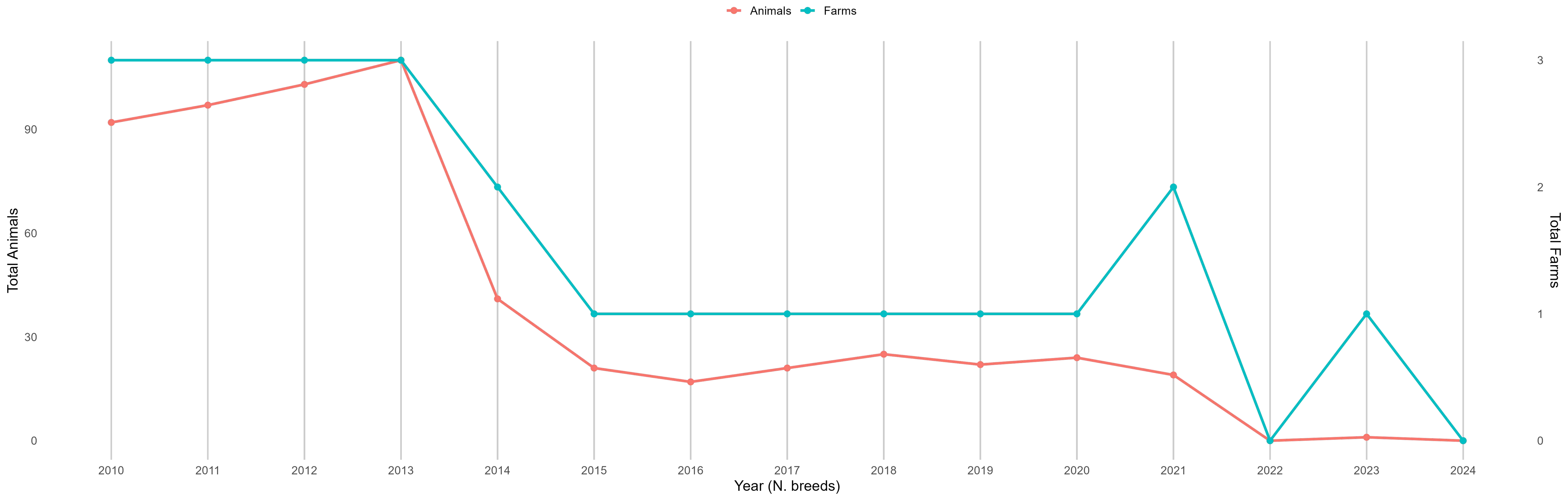

Animals
Farms
Total Animals
Total Farms
Year (N. breeds)
0
30
60
90
0
1
2
3
2010
2011
2012
2013
2014
2015
2016
2017
2018
2019
2020
2021
2022
2023
2024

GARFAGNINA BIANCA

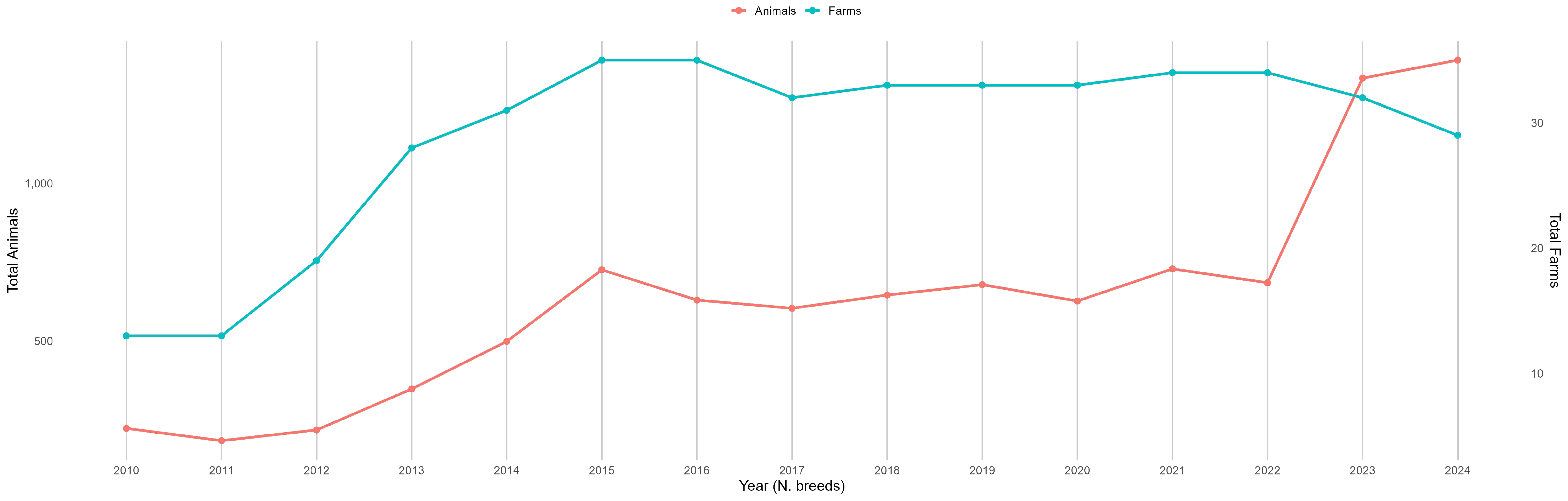

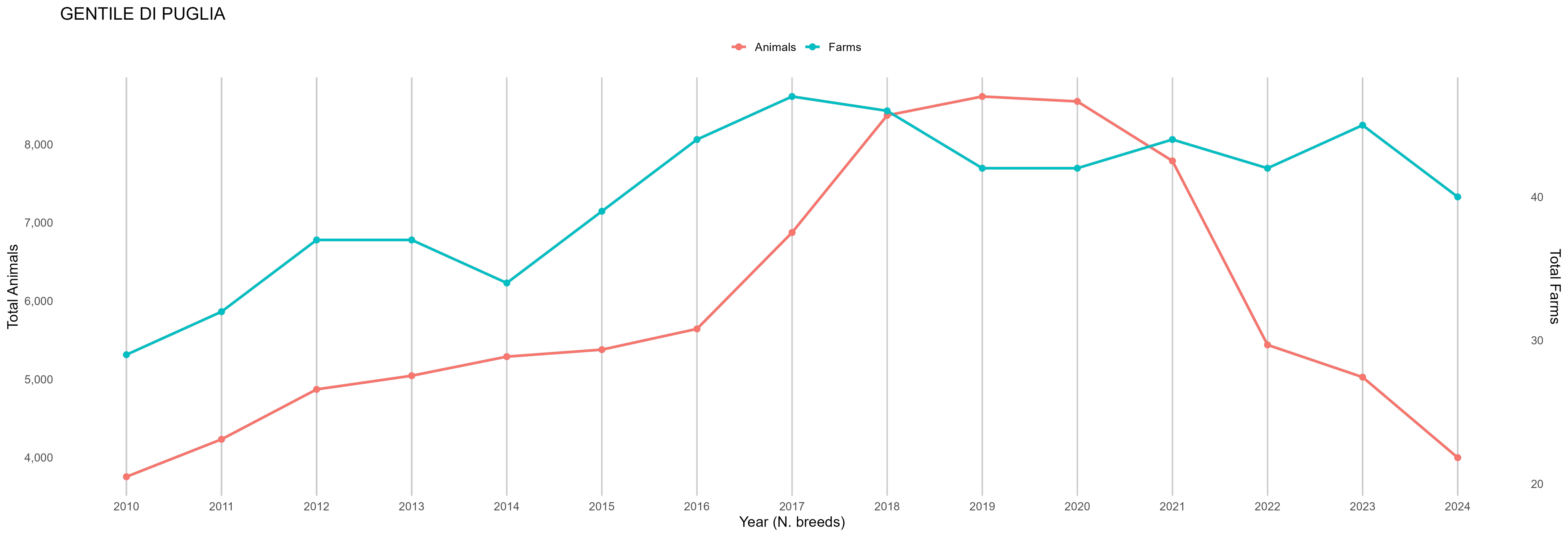

GENTILE DI PUGLIA
Animals
Farms
8,000
7,000
6,000
5,000
4,000
Total Animals
40
30
20
Total Farms
2010
2011
2012
2013
2014
2015
2016
2017
2018
2019
2020
2021
2022
2023
2024
Year (N. breeds)

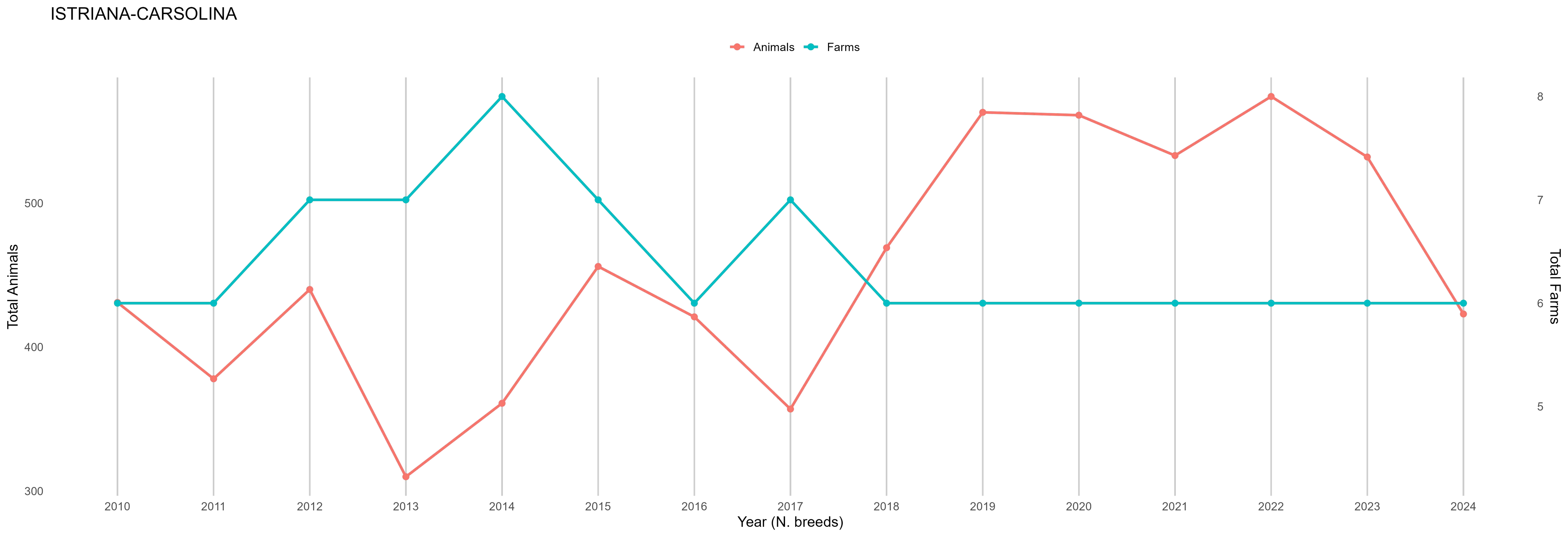

ISTRIANA-CARSOLINA
Animals
Farms
Total Animals
Total Farms
Year (N. breeds)
300
400
500
5
6
7
8
2010
2011
2012
2013
2014
2015
2016
2017
2018
2019
2020
2021
2022
2023
2024

JURASCHAF-GIURASSICA

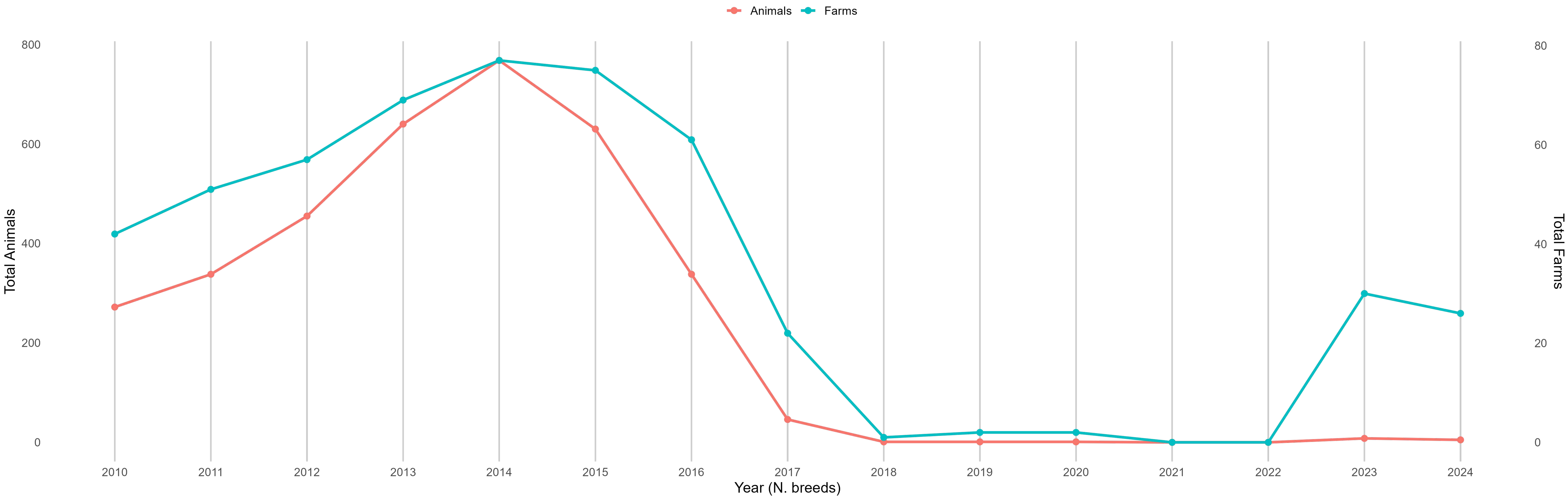

LAMON

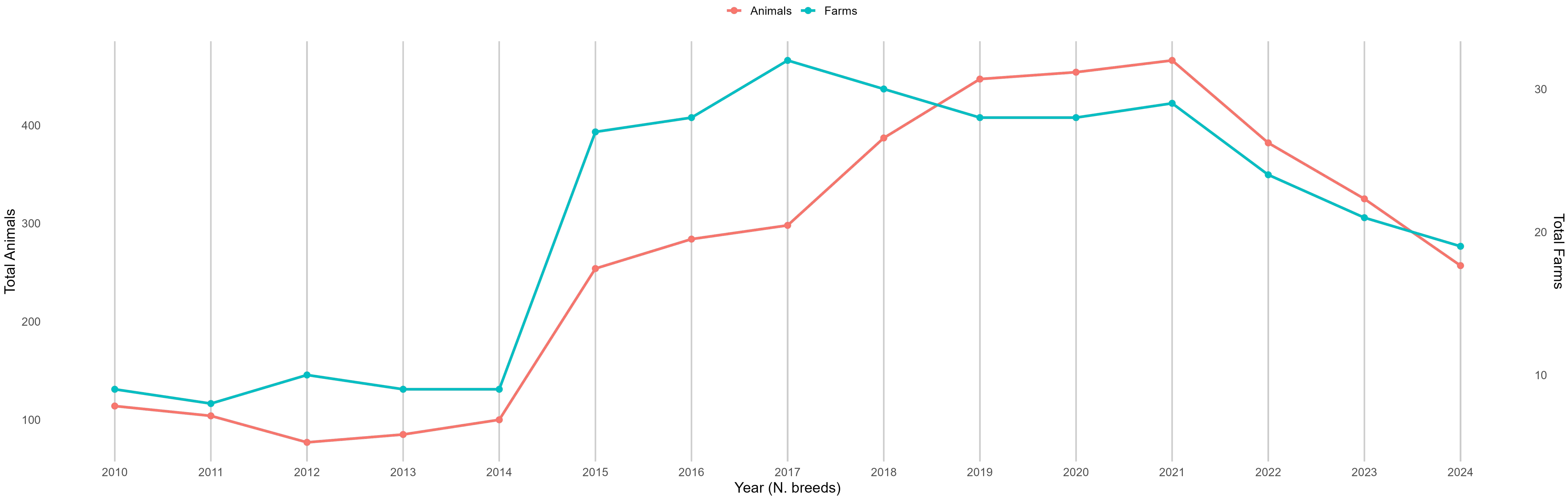

Animals
Farms
Total Animals
Total Farms
100
200
300
400
10
20
30
2010
2011
2012
2013
2014
2015
2016
2017
2018
2019
2020
2021
2022
2023
2024
Year (N. breeds)

LATICAUDA

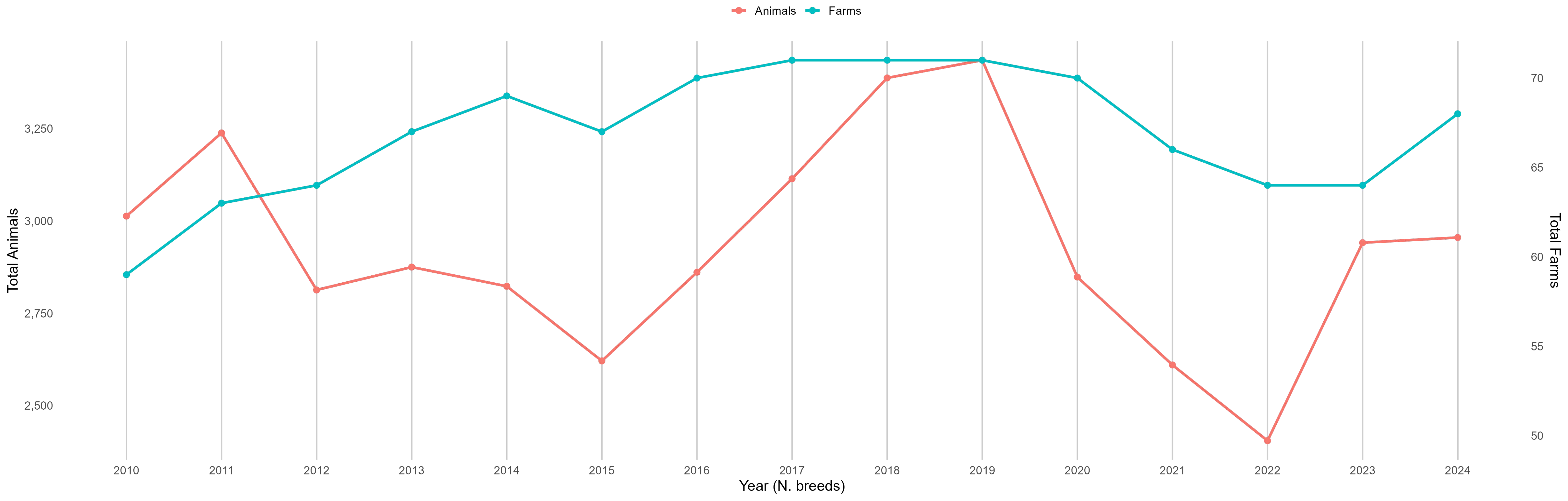
Animals
Farms
Total Animals
3,250
3,000
2,750
2,500
Total Farms
70
65
60
55
50
2010
2011
2012
2013
2014
2015
2016
2017
2018
2019
2020
2021
2022
2023
2024
Year (N. breeds)

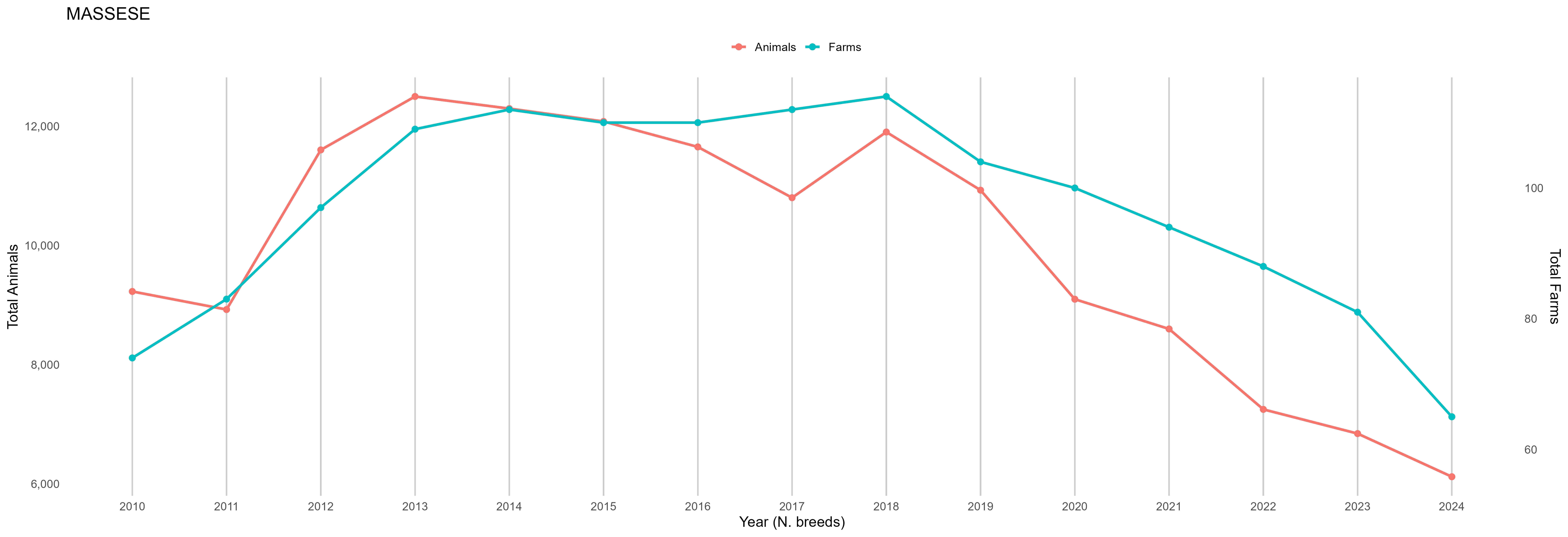
MASSESE
Animals
Farms
Total Animals
Total Farms
12,000
10,000
8,000
6,000
100
80
60
2010
2011
2012
2013
2014
2015
2016
2017
2018
2019
2020
2021
2022
2023
2024
Year (N. breeds)

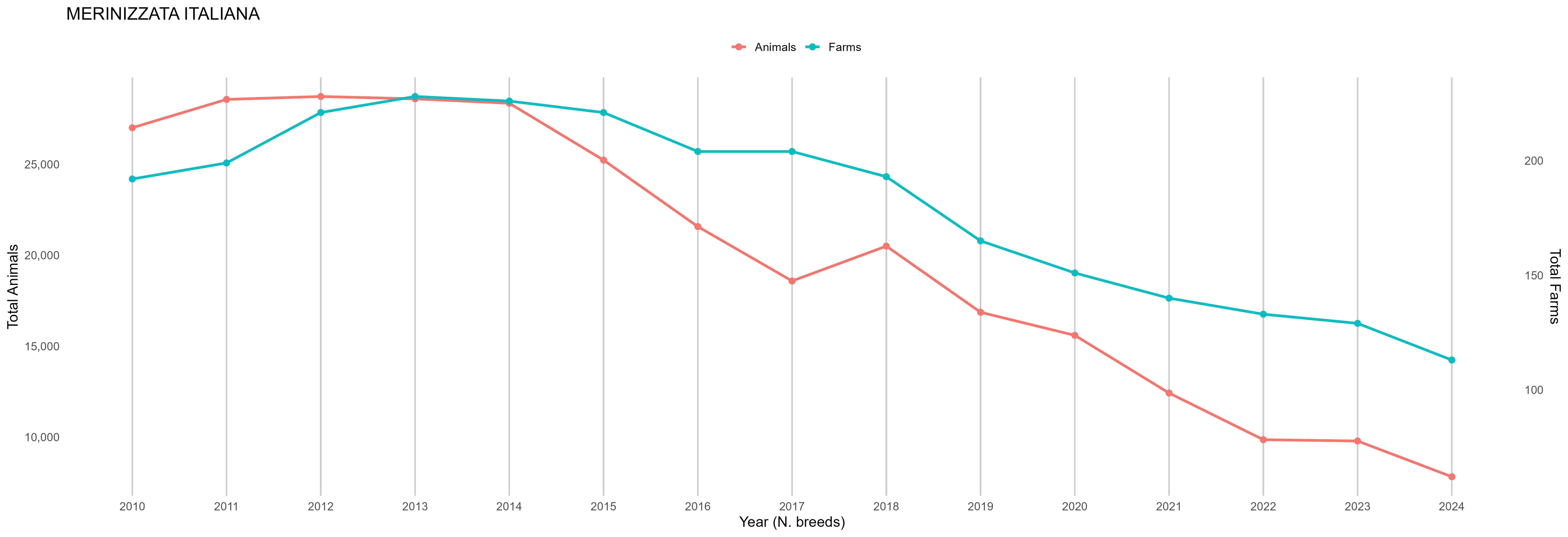

MERINIZZATA ITALIANA
Animals
Farms
Total Animals
25,000
20,000
15,000
10,000
Total Farms
200
150
100
2010
2011
2012
2013
2014
2015
2016
2017
2018
2019
2020
2021
2022
2023
2024
Year (N. breeds)

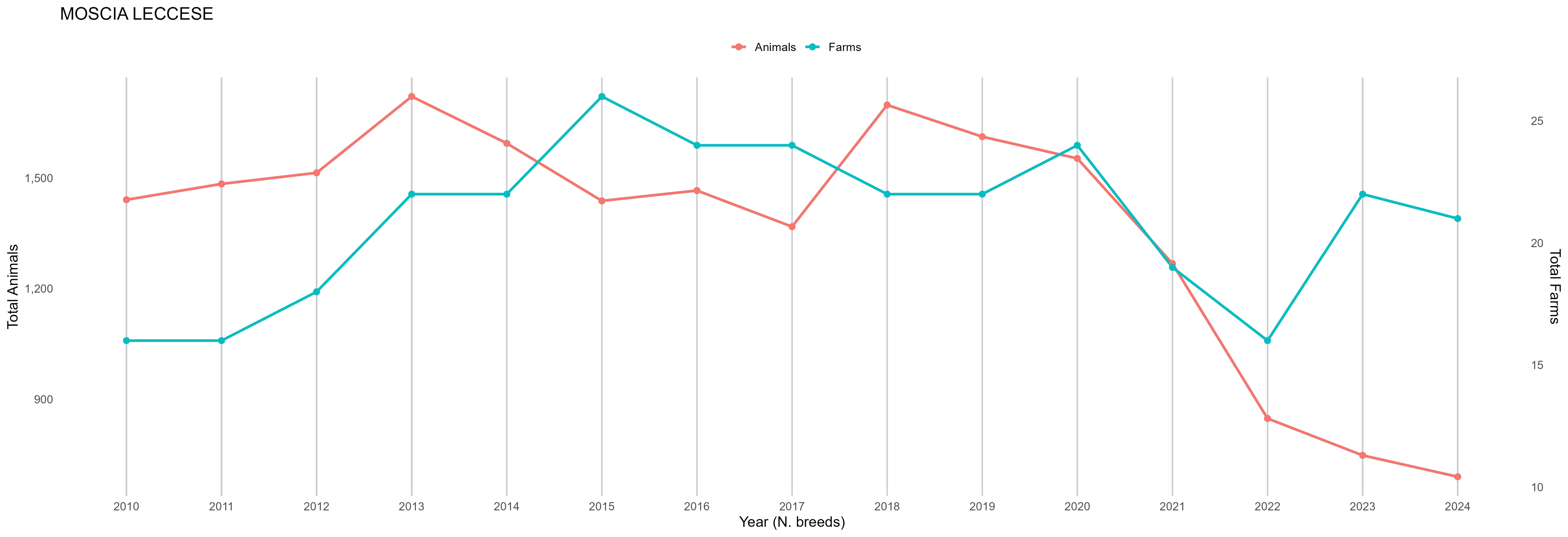
MOSCIA LECCESE
Animals
Farms
Total Animals
Total Farms
Year (N. breeds)
1,500
1,200
900
25
20
15
10
2010
2011
2012
2013
2014
2015
2016
2017
2018
2019
2020
2021
2022
2023
2024

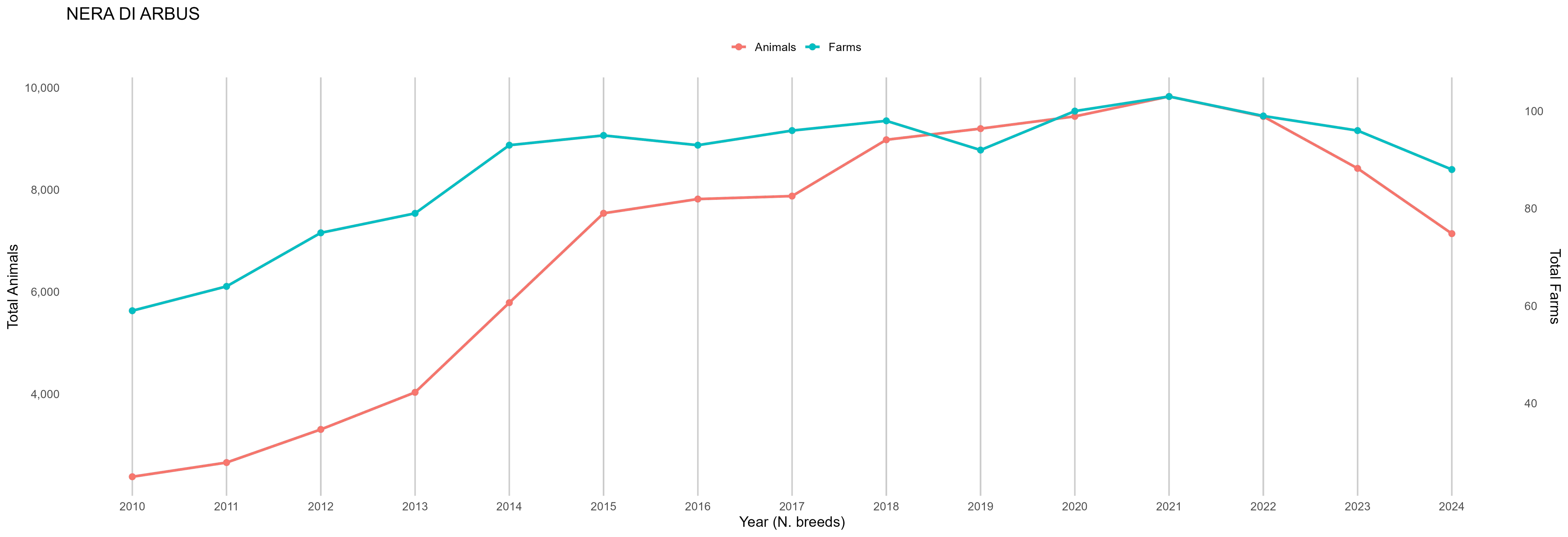
NERA DI ARBUS
Animals
Farms
10,000
8,000
6,000
4,000
Total Animals
100
80
60
40
Total Farms
2010
2011
2012
2013
2014
2015
2016
2017
2018
2019
2020
2021
2022
2023
2024
Year (N. breeds)

NOSTRANA

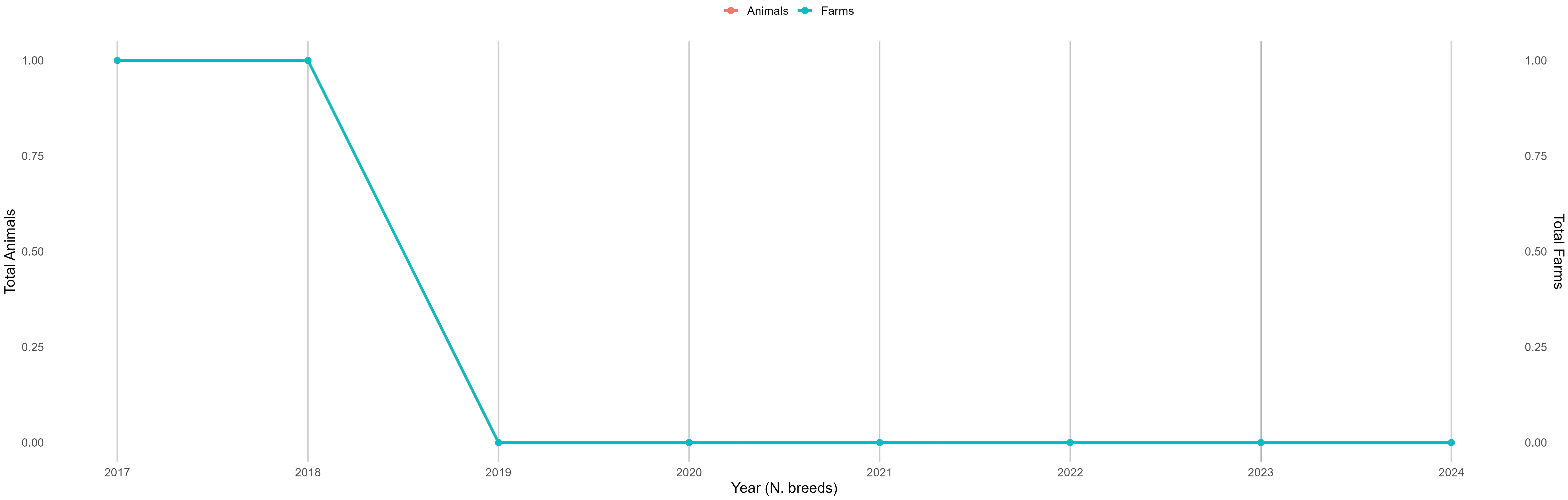

Animals
Farms
Total Animals
Total Farms
1.00
0.75
0.50
0.25
0.00
2017
2018
2019
2020
2021
2022
2023
2024
Year (N. breeds)

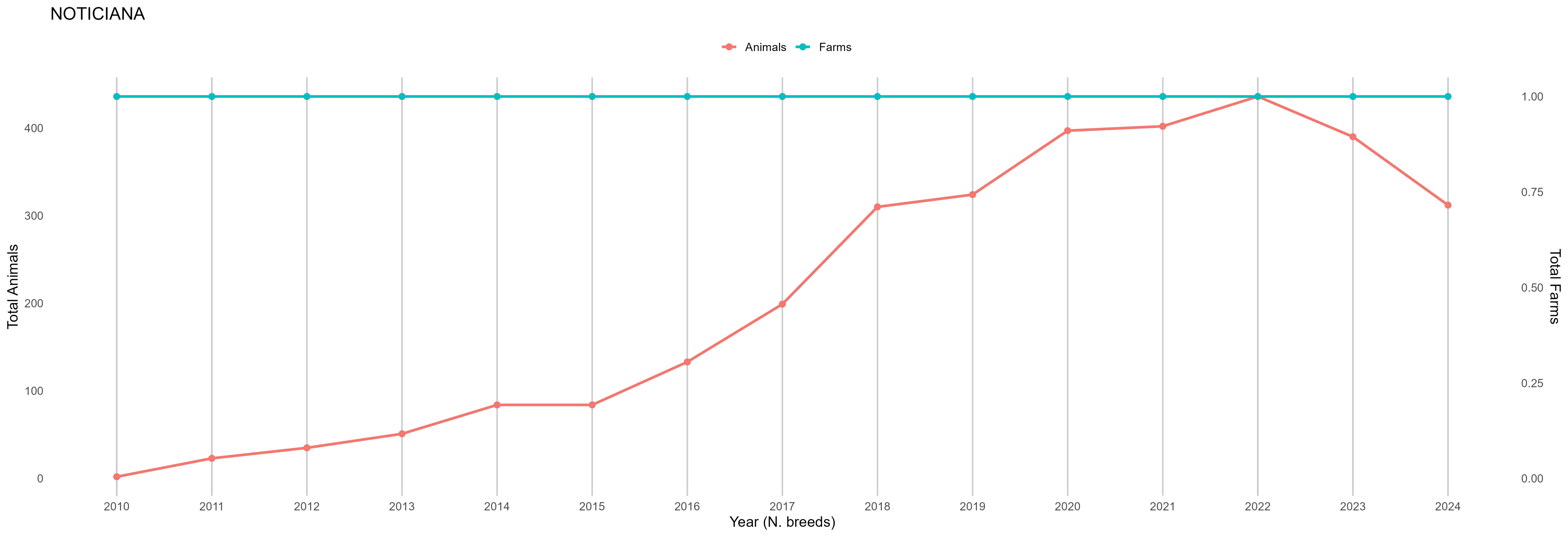
NOTICIANA
Animals
Farms
Total Animals
Total Farms
Year (N. breeds)
0
100
200
300
400
0.00
0.25
0.50
0.75
1.00
2010
2011
2012
2013
2014
2015
2016
2017
2018
2019
2020
2021
2022
2023
2024

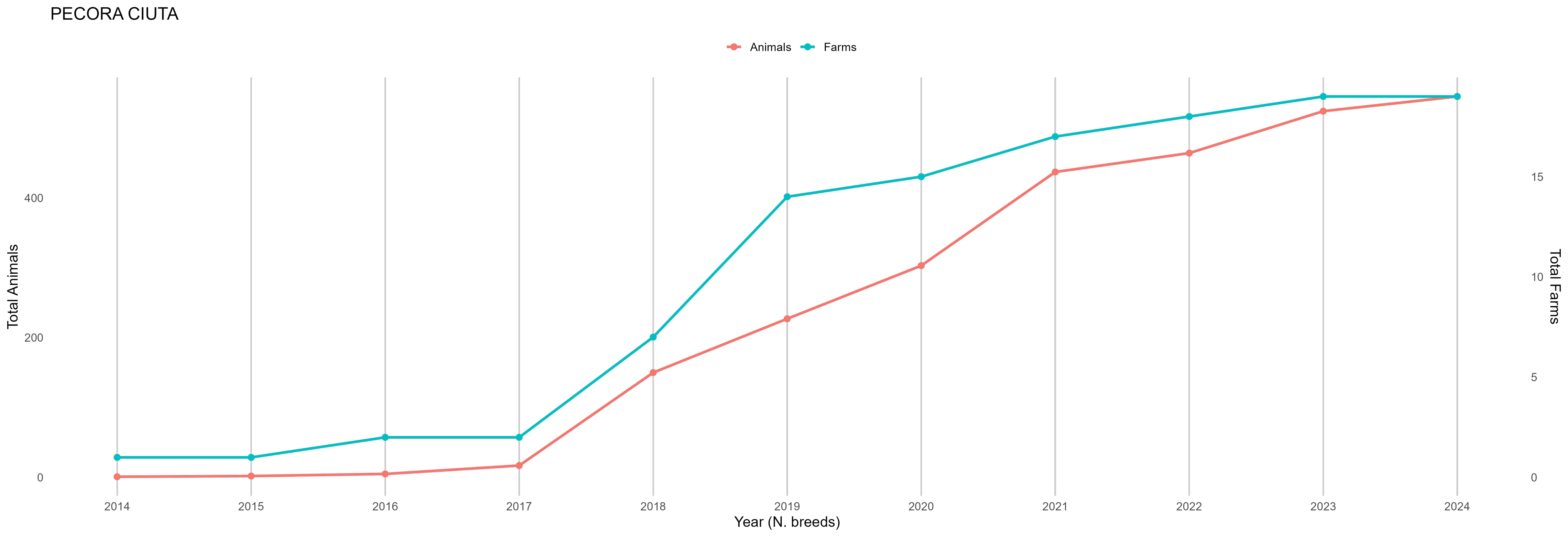
PECORA CIUTA
Animals
Farms
Total Animals
0
200
400
Total Farms
0
5
10
15
2014
2015
2016
2017
2018
2019
2020
2021
2022
2023
2024
Year (N. breeds)

PECORA DI CORTENO

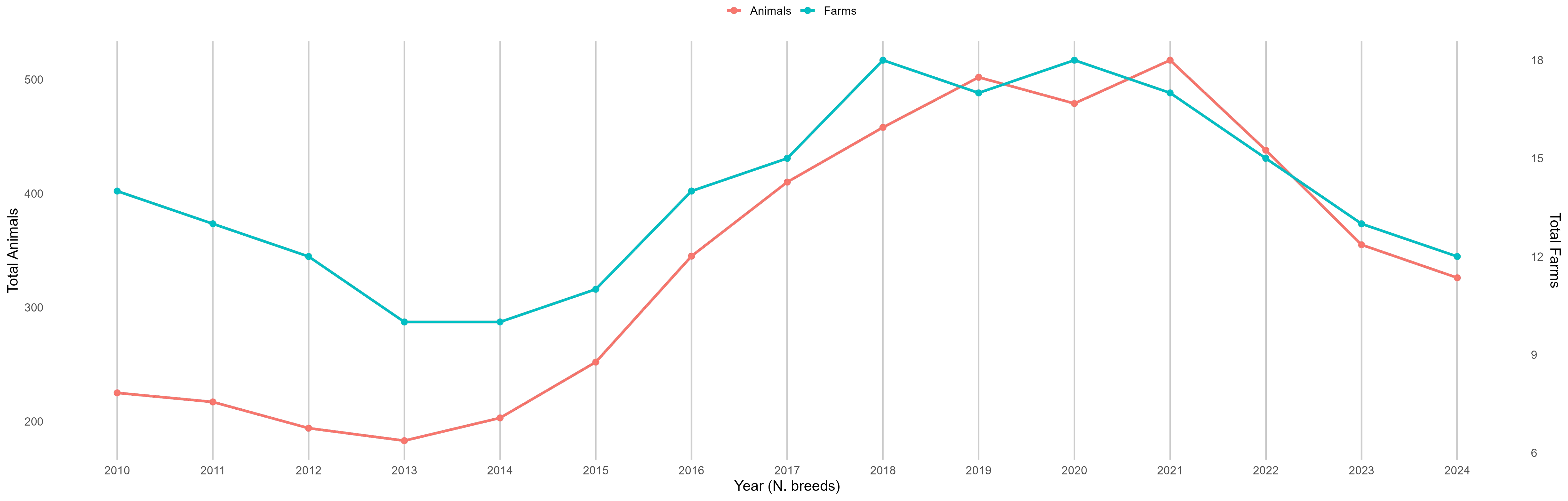

PINZIRITA

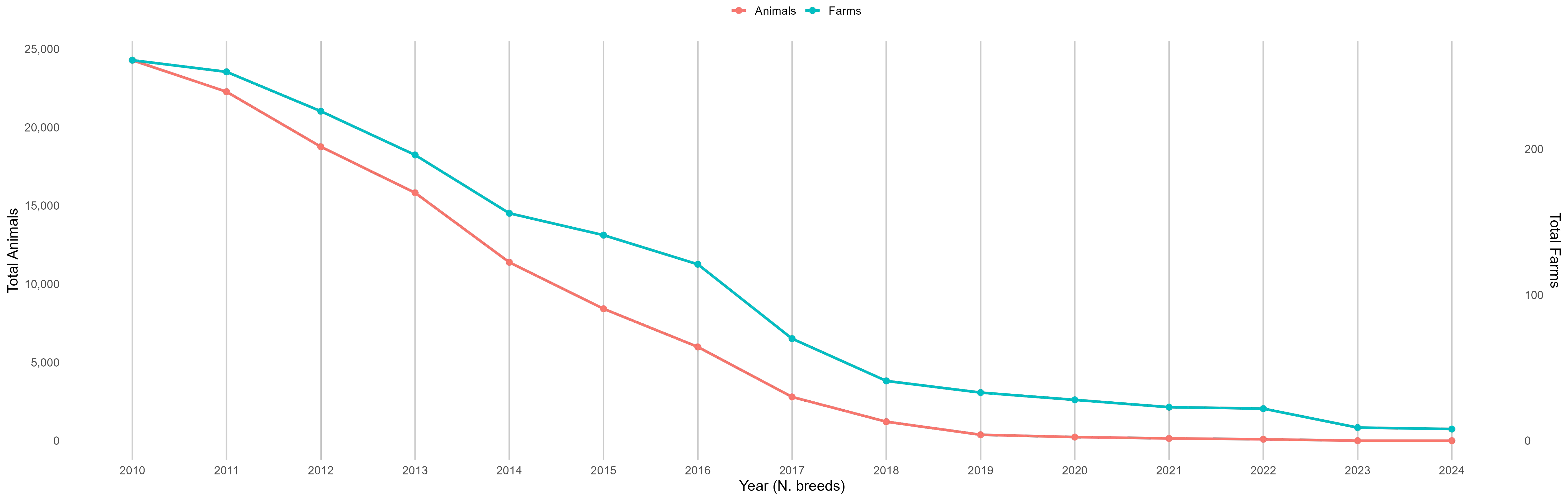

Animals
Farms
25,000
20,000
15,000
10,000
5,000
0
Total Animals
200
100
0
Total Farms
2010
2011
2012
2013
2014
2015
2016
2017
2018
2019
2020
2021
2022
2023
2024
Year (N. breeds)

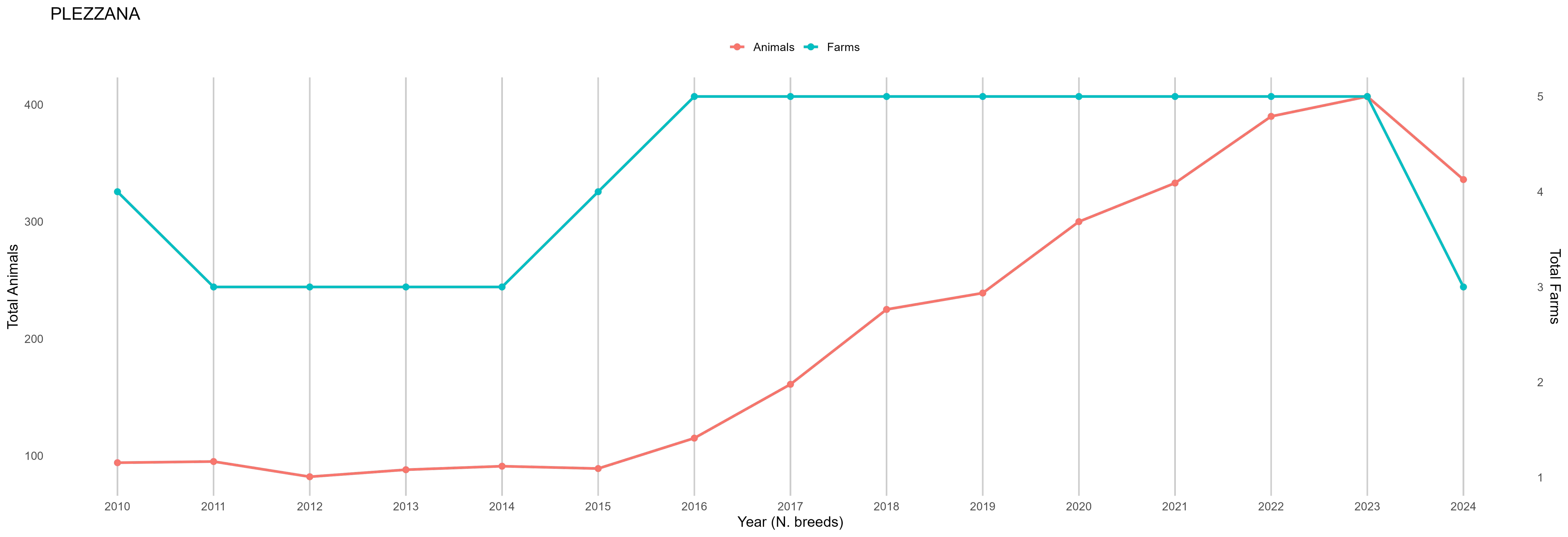
PLEZZANA
Animals
Farms
Total Animals
Total Farms
Year (N. breeds)
100
200
300
400
1
2
3
4
5
2010
2011
2012
2013
2014
2015
2016
2017
2018
2019
2020
2021
2022
2023
2024

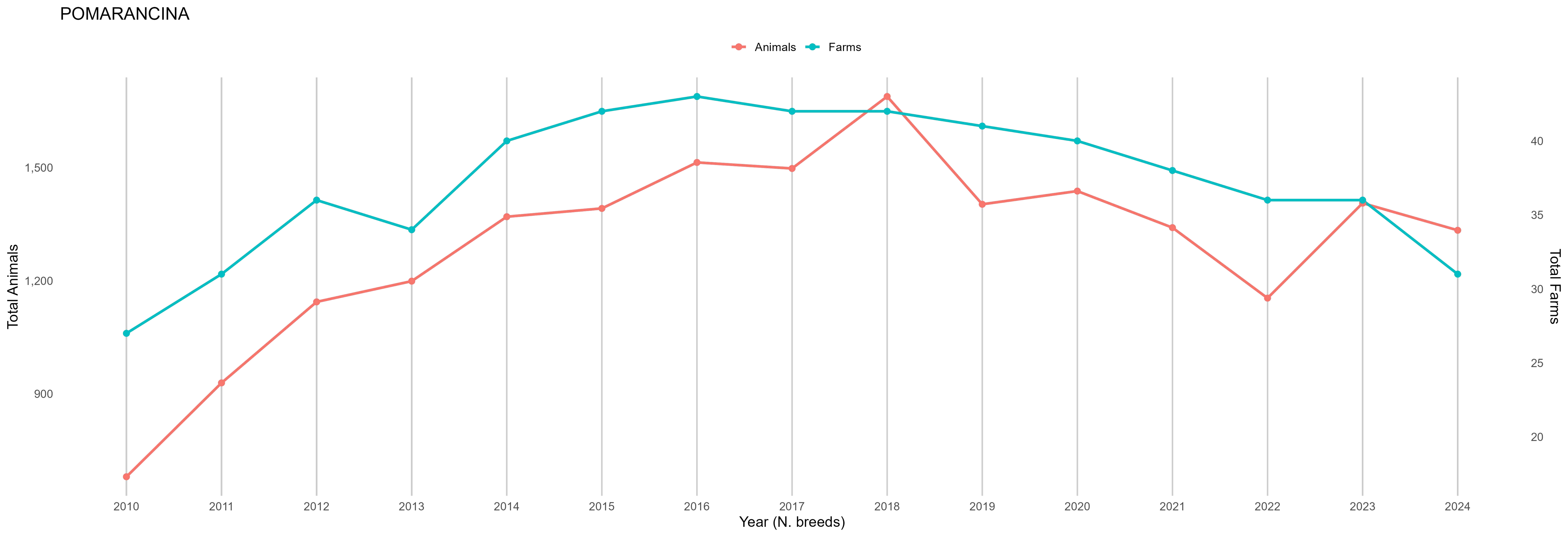

POMARANCINA
Animals
Farms
Total Animals
1,500
1,200
900
Total Farms
40
35
30
25
20
2010
2011
2012
2013
2014
2015
2016
2017
2018
2019
2020
2021
2022
2023
2024
Year (N. breeds)

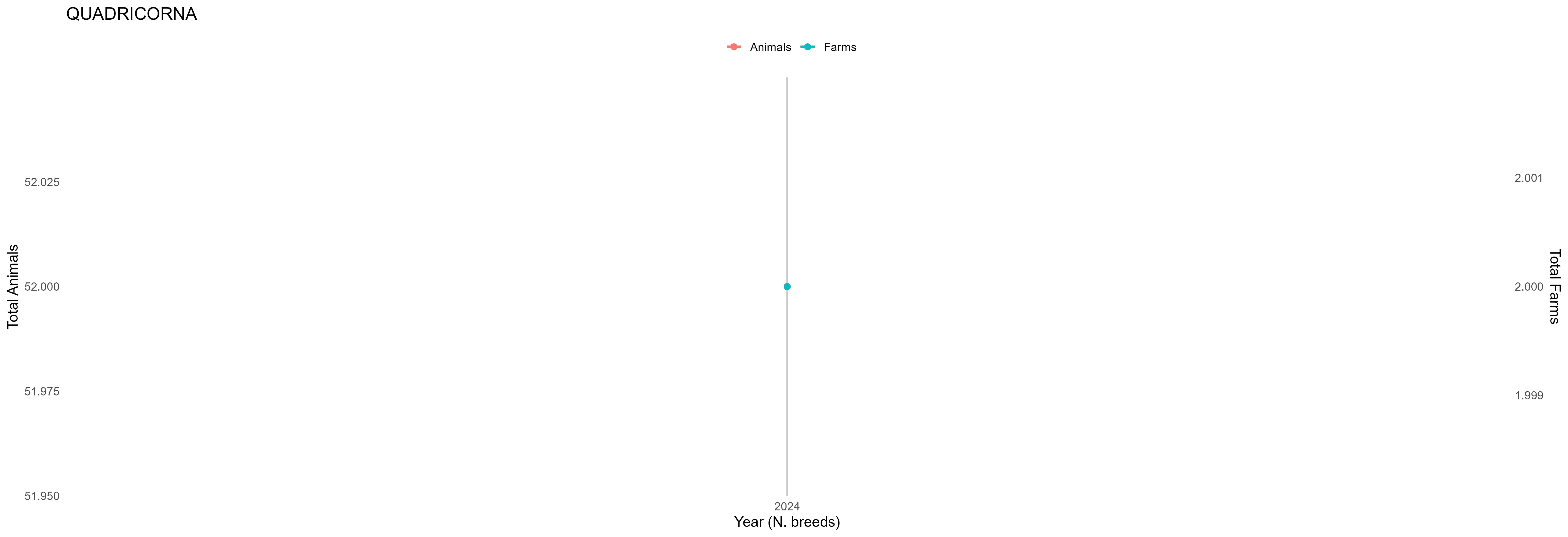
QUADRICORNA
Animals
Farms
Total Animals
52.025
52.000
51.975
51.950
Total Farms
2.001
2.000
1.999
2024
Year (N. breeds)

ROSSET

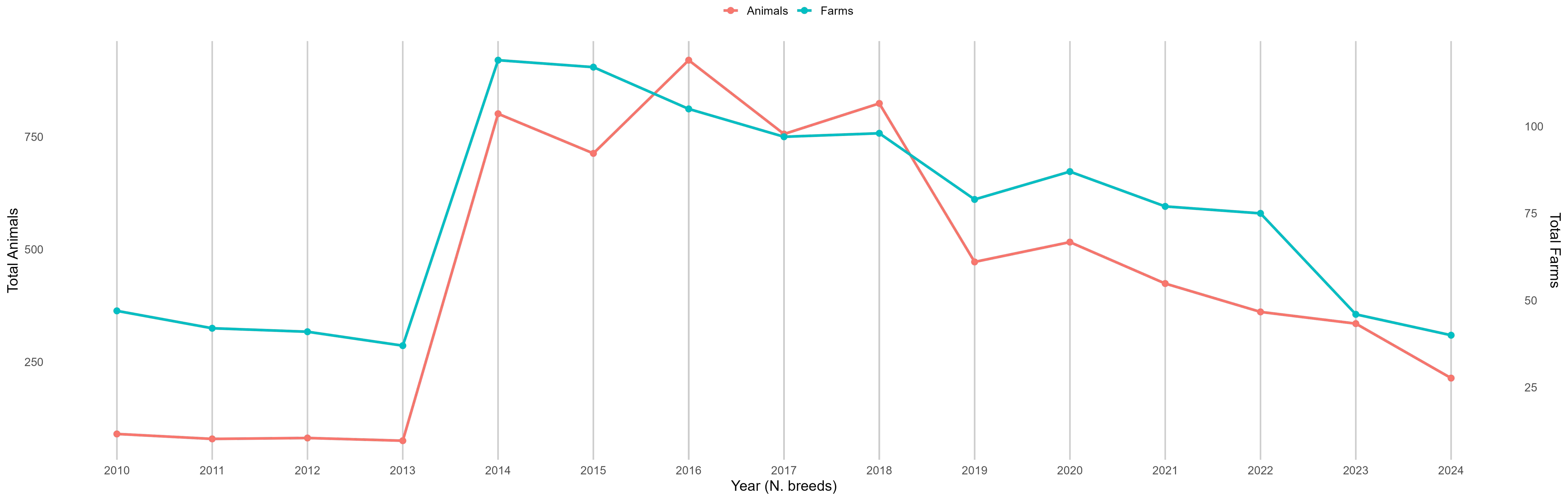

Animals
Farms
Total Animals
750
500
250
Total Farms
100
75
50
25
2010
2011
2012
2013
2014
2015
2016
2017
2018
2019
2020
2021
2022
2023
2024
Year (N. breeds)

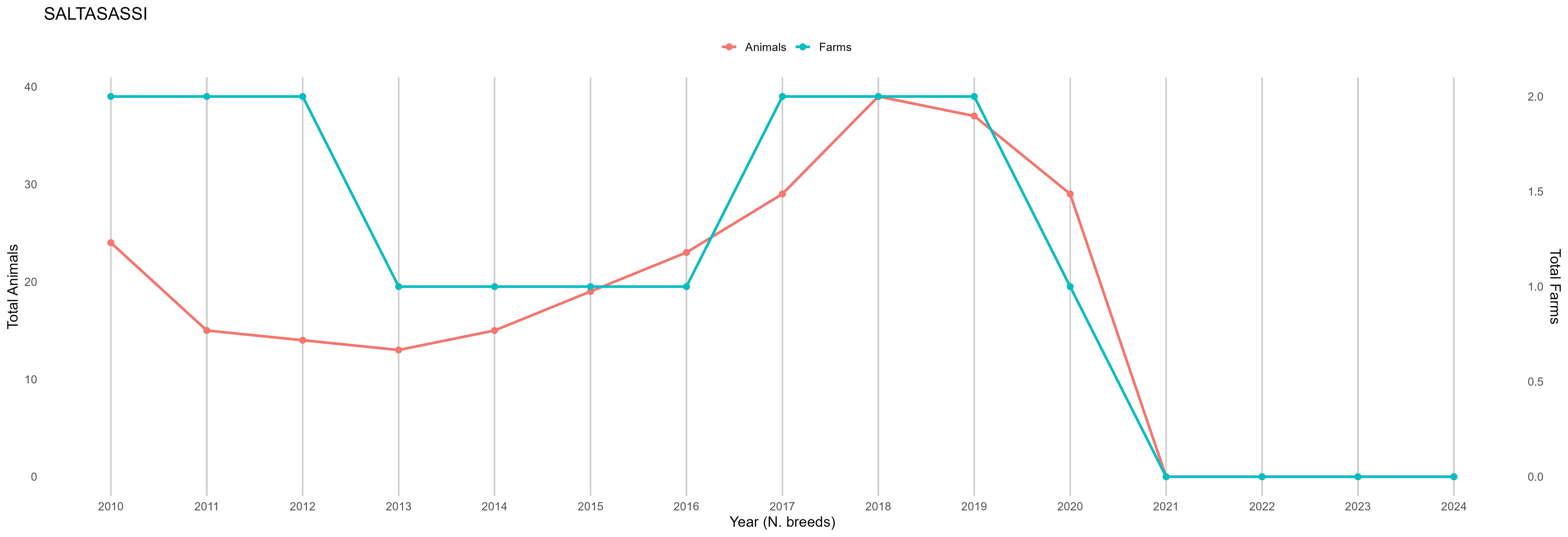
SALTASASSI
Animals
Farms
Total Animals
Total Farms
Year (N. breeds)
0
10
20
30
40
0.0
0.5
1.0
1.5
2.0
2010
2011
2012
2013
2014
2015
2016
2017
2018
2019
2020
2021
2022
2023
2024

SAMBUCANA

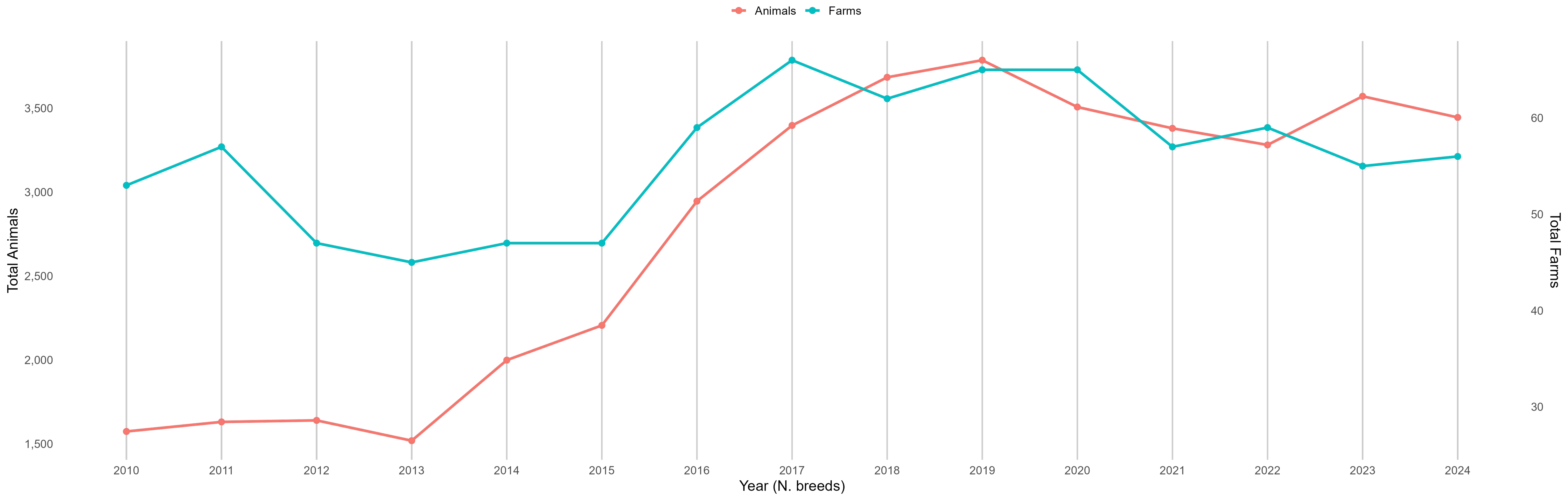

Animals
Farms
3,500
3,000
2,500
2,000
1,500
Total Animals
60
50
40
30
Total Farms
2010
2011
2012
2013
2014
2015
2016
2017
2018
2019
2020
2021
2022
2023
2024
Year (N. breeds)

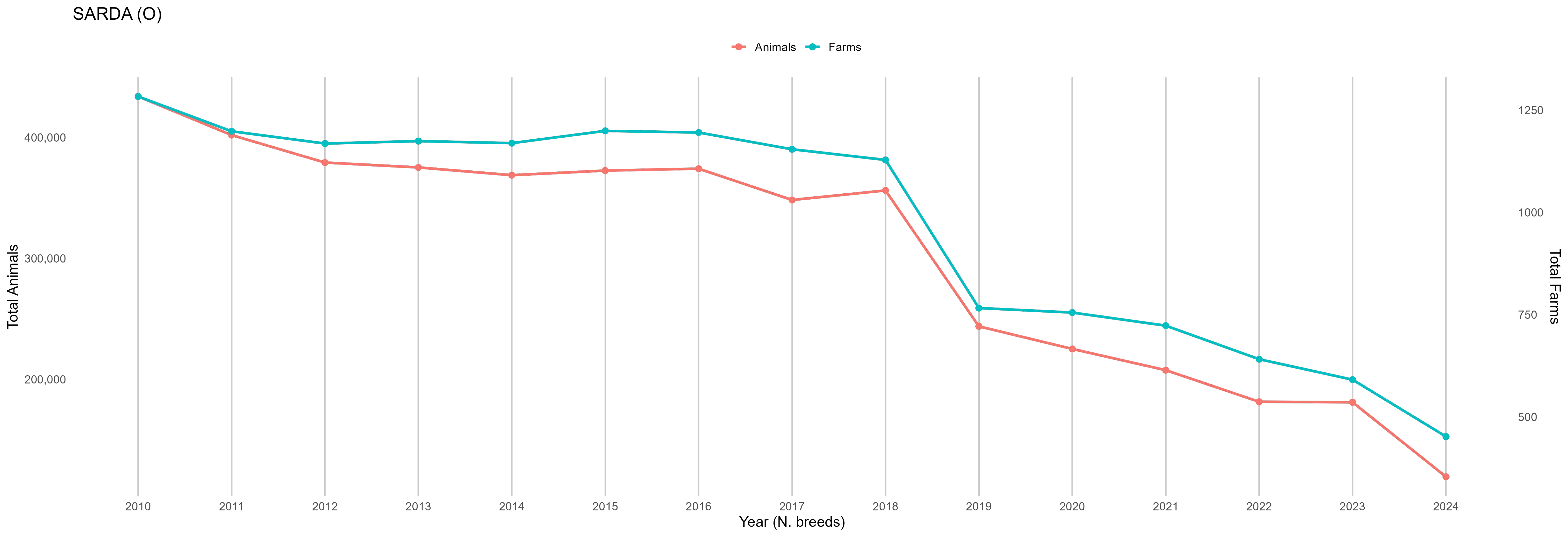

SARDA (O)
Animals
Farms
Total Animals
400,000
300,000
200,000
Total Farms
1250
1000
750
500
2010
2011
2012
2013
2014
2015
2016
2017
2018
2019
2020
2021
2022
2023
2024
Year (N. breeds)

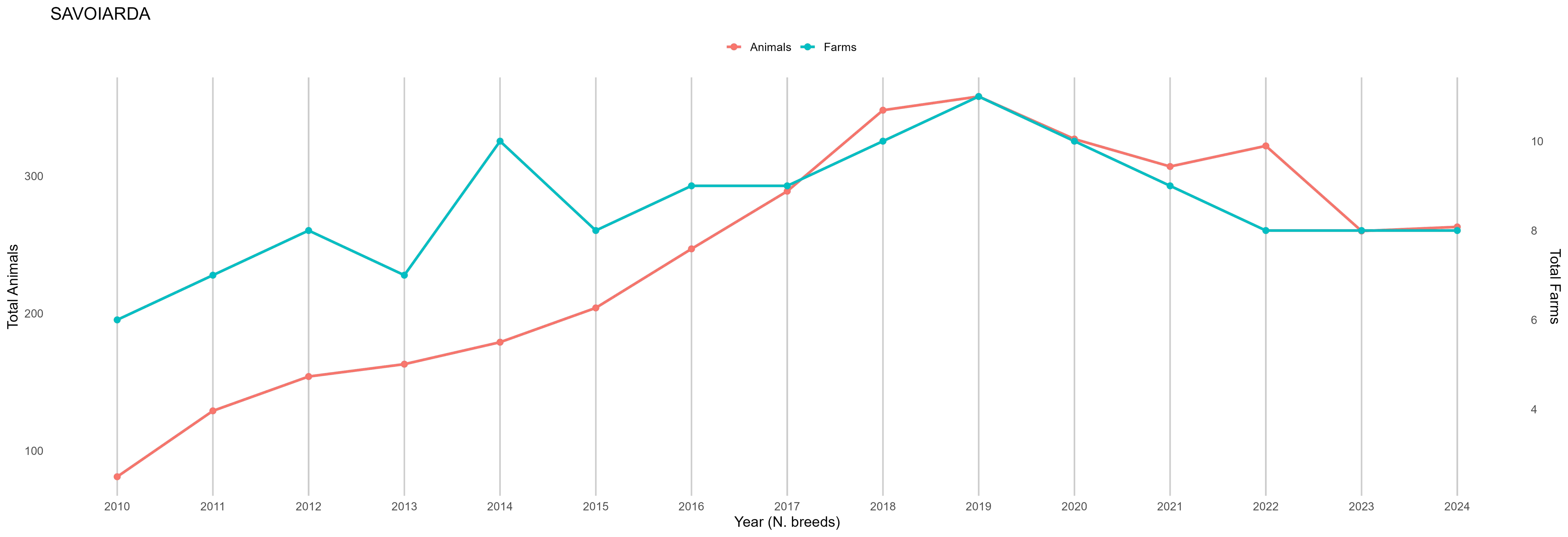
SAVOIARDA
Animals
Farms
Total Animals
Total Farms
Year (N. breeds)
100
200
300
4
6
8
10
2010
2011
2012
2013
2014
2015
2016
2017
2018
2019
2020
2021
2022
2023
2024

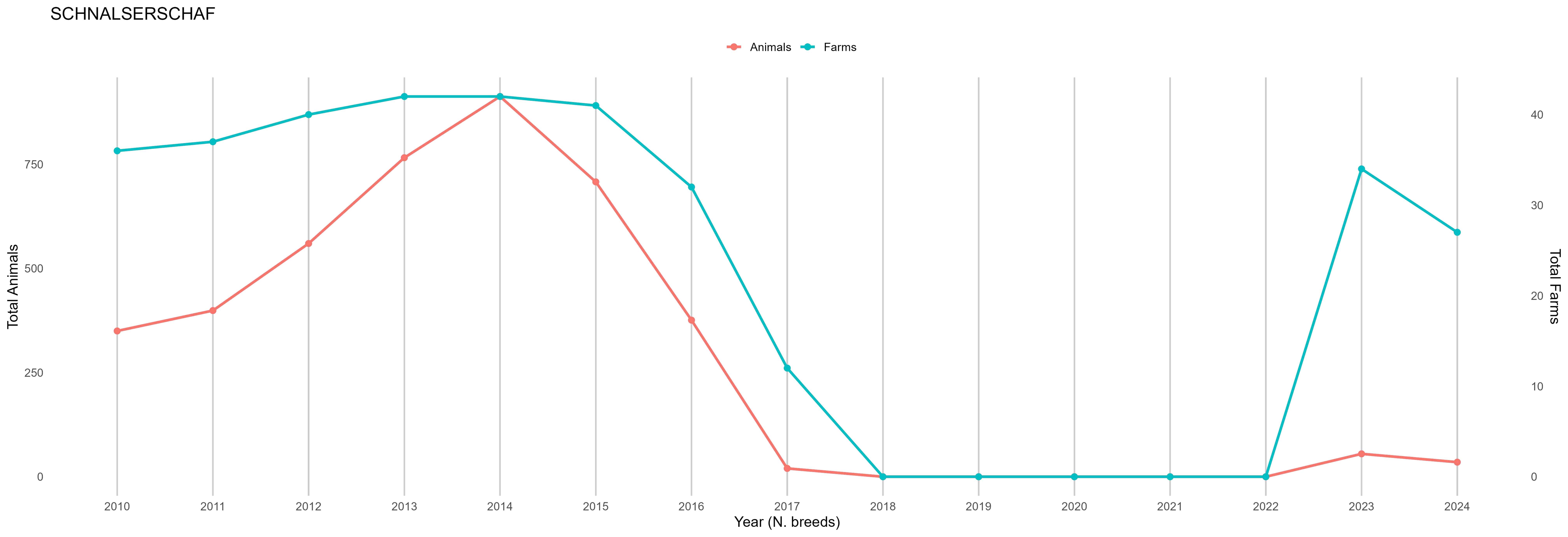
SCHNALSERSCHAF
Animals
Farms
Total Animals
0
250
500
750
Total Farms
0
10
20
30
40
2010
2011
2012
2013
2014
2015
2016
2017
2018
2019
2020
2021
2022
2023
2024
Year (N. breeds)

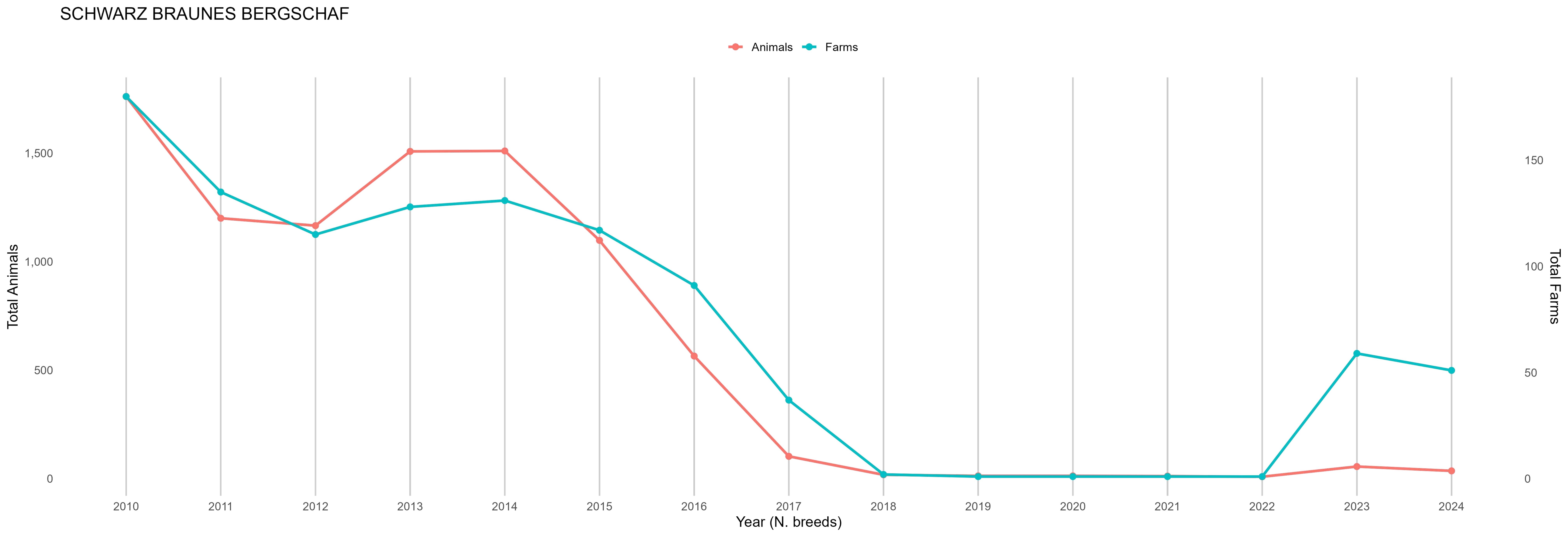

SCHWARZ BRAUNES BERGSCHAF
Animals
Farms
Total Animals
Total Farms
Year (N. breeds)
1,500
1,000
500
0
150
100
50
0
2010
2011
2012
2013
2014
2015
2016
2017
2018
2019
2020
2021
2022
2023
2024

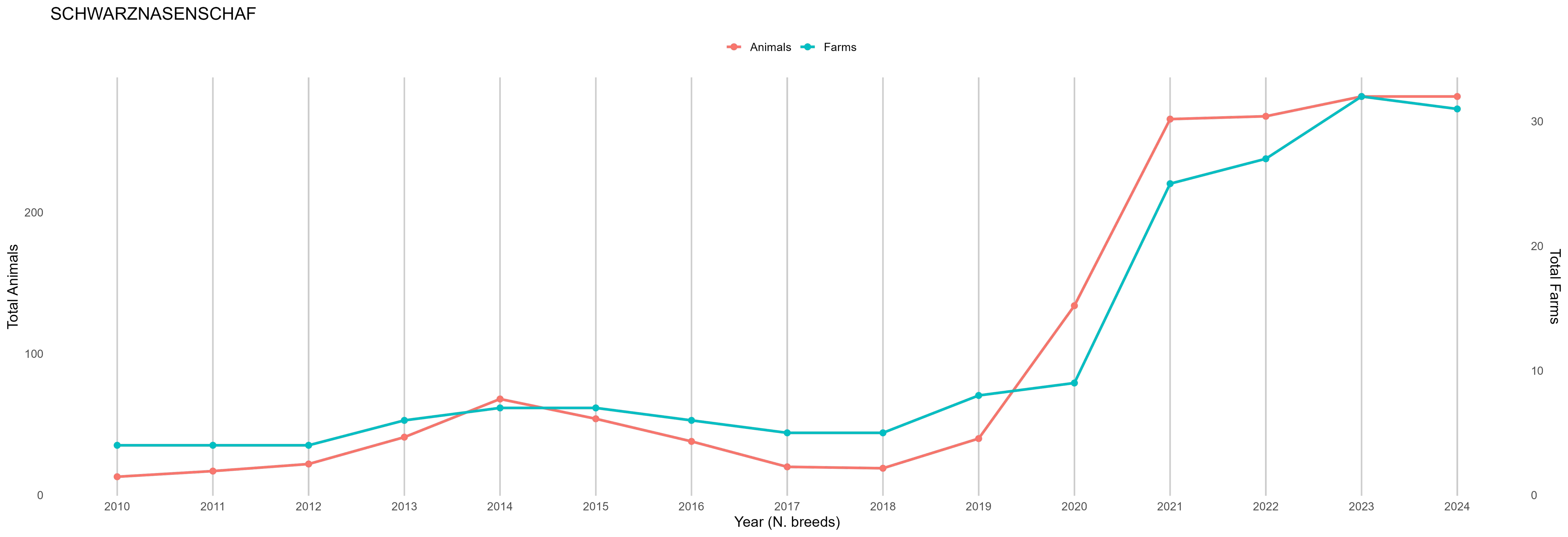

SCHWARZNASENSCHAF
Animals
Farms
Total Animals
Total Farms
Year (N. breeds)
0
100
200
0
10
20
30
2010
2011
2012
2013
2014
2015
2016
2017
2018
2019
2020
2021
2022
2023
2024

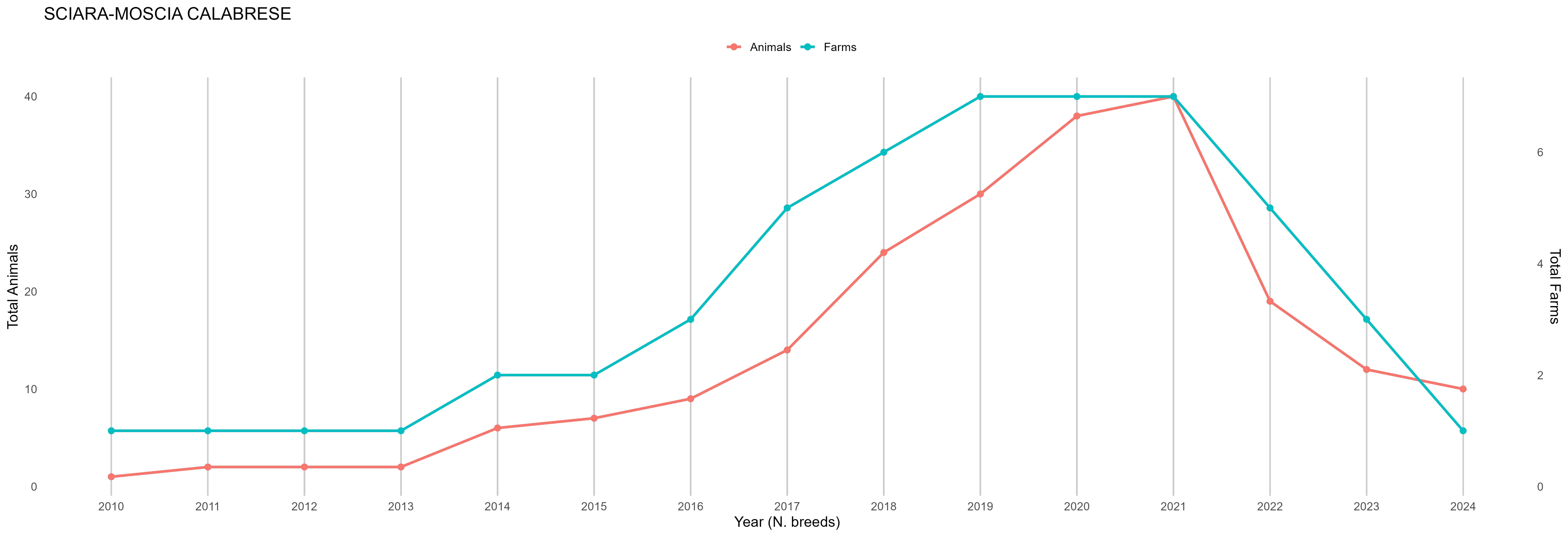
SCIARA-MOSCIA CALABRESE
Animals
Farms
Total Animals
Total Farms
Year (N. breeds)
0
10
20
30
40
0
2
4
6
2010
2011
2012
2013
2014
2015
2016
2017
2018
2019
2020
2021
2022
2023
2024

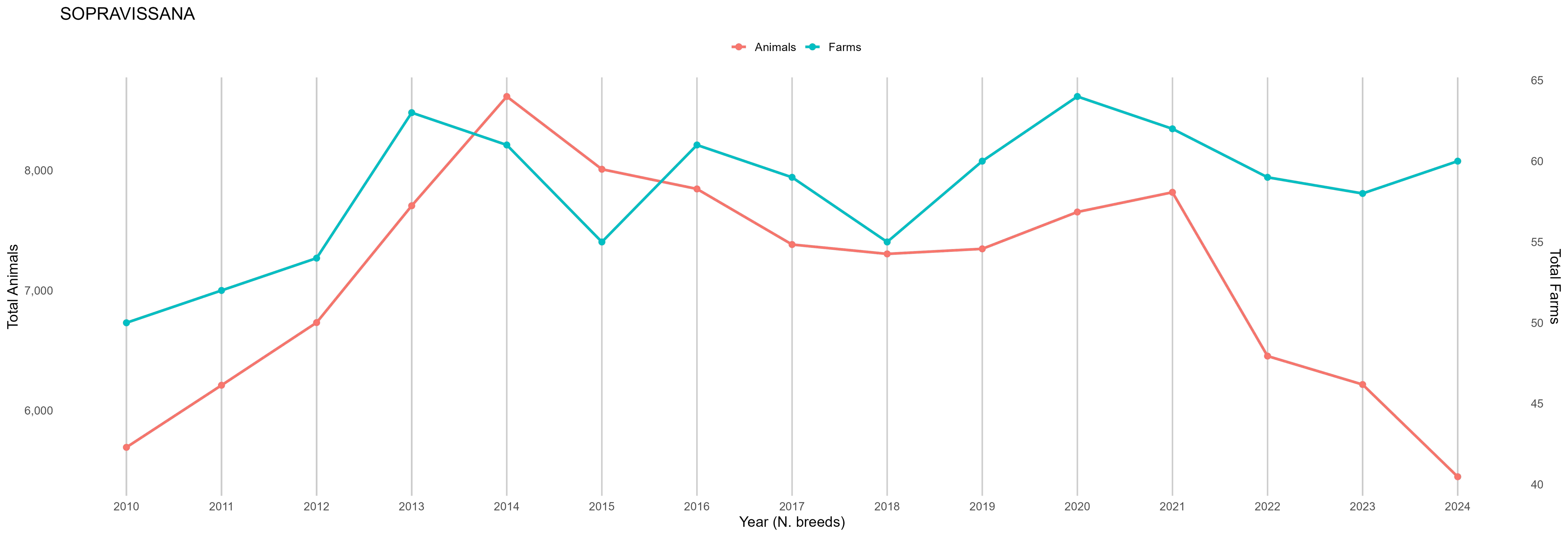
SOPRAVISSANA
Animals
Farms
Total Animals
8,000
7,000
6,000
Total Farms
65
60
55
50
45
40
2010
2011
2012
2013
2014
2015
2016
2017
2018
2019
2020
2021
2022
2023
2024
Year (N. breeds)

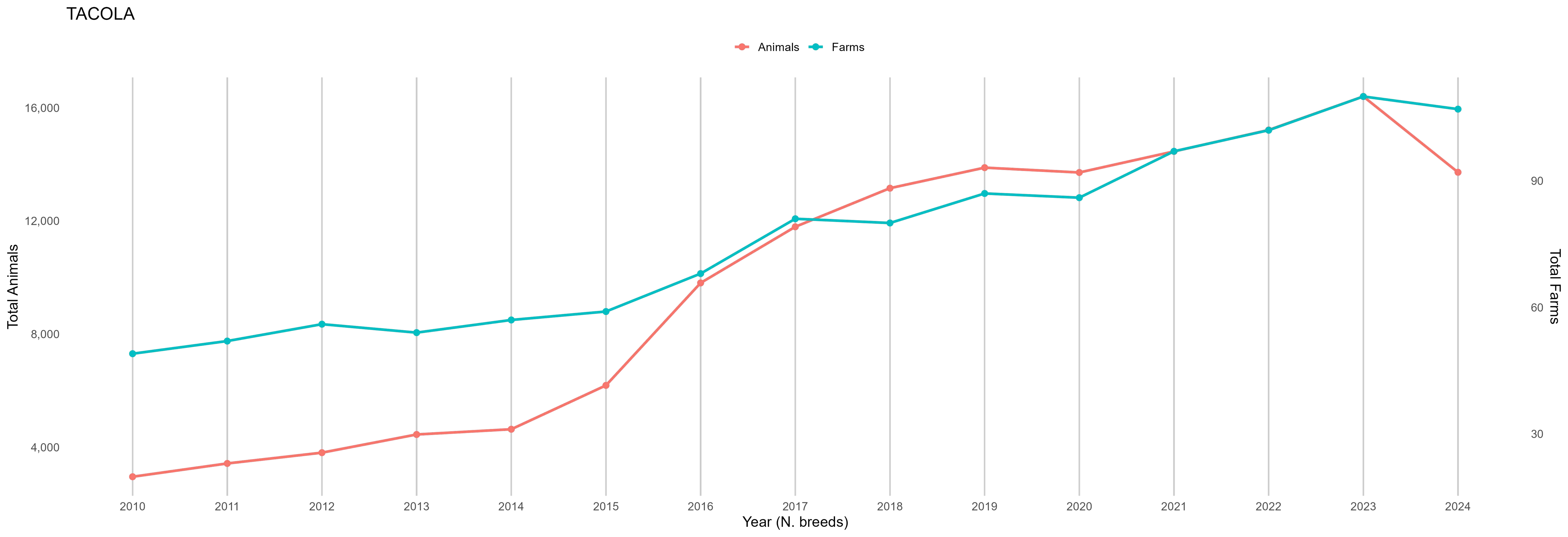

TACOLA
Animals
Farms
16,000
12,000
8,000
4,000
Total Animals
90
60
30
Total Farms
2010
2011
2012
2013
2014
2015
2016
2017
2018
2019
2020
2021
2022
2023
2024
Year (N. breeds)

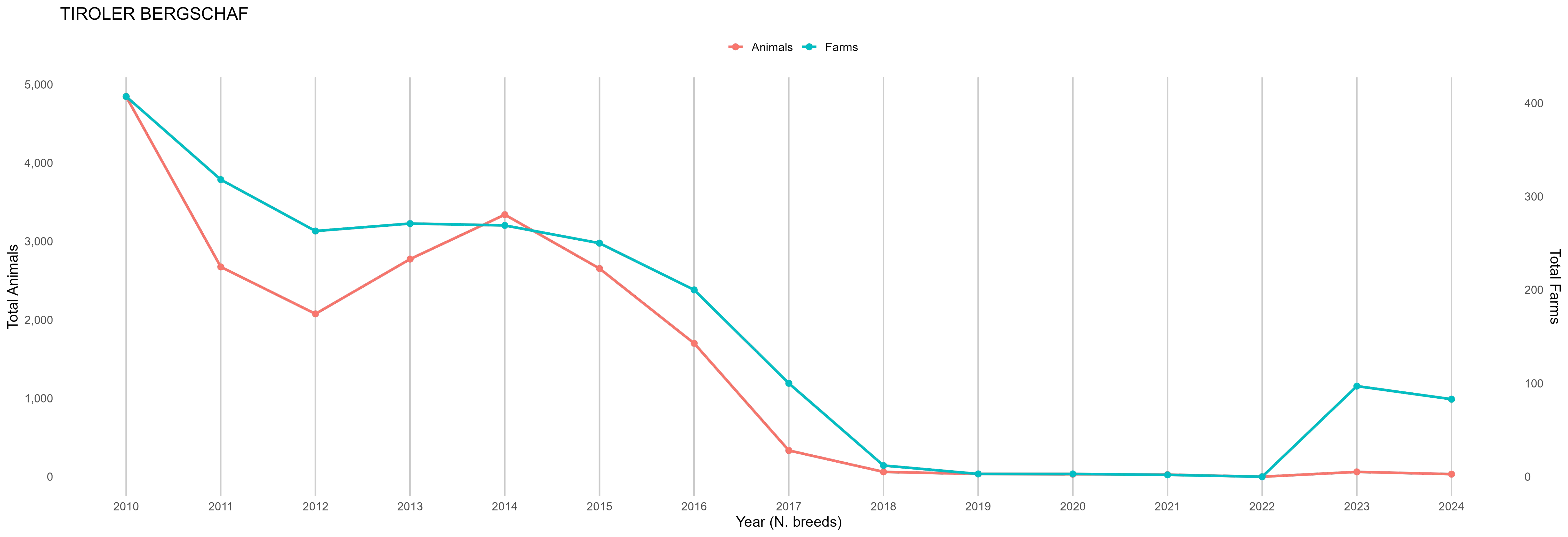

TIROLER BERGSCHAF
Animals
Farms
Total Animals
Total Farms
Year (N. breeds)
5,000
4,000
3,000
2,000
1,000
0
400
300
200
100
0
2010
2011
2012
2013
2014
2015
2016
2017
2018
2019
2020
2021
2022
2023
2024

TIROLER STEINSCHAF

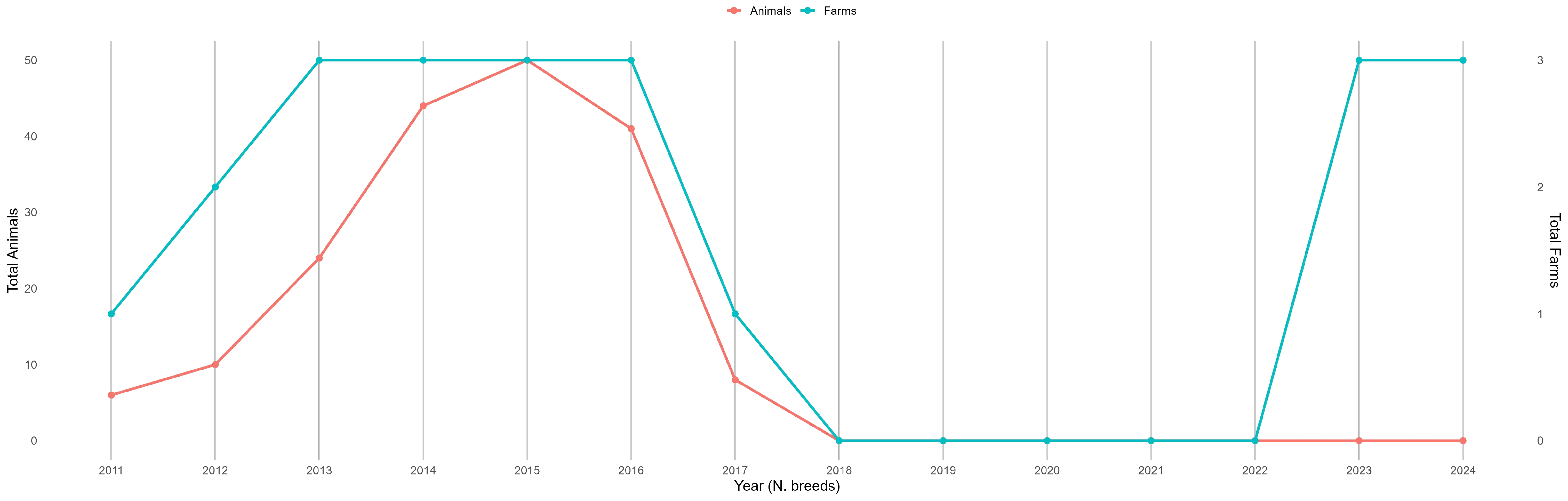

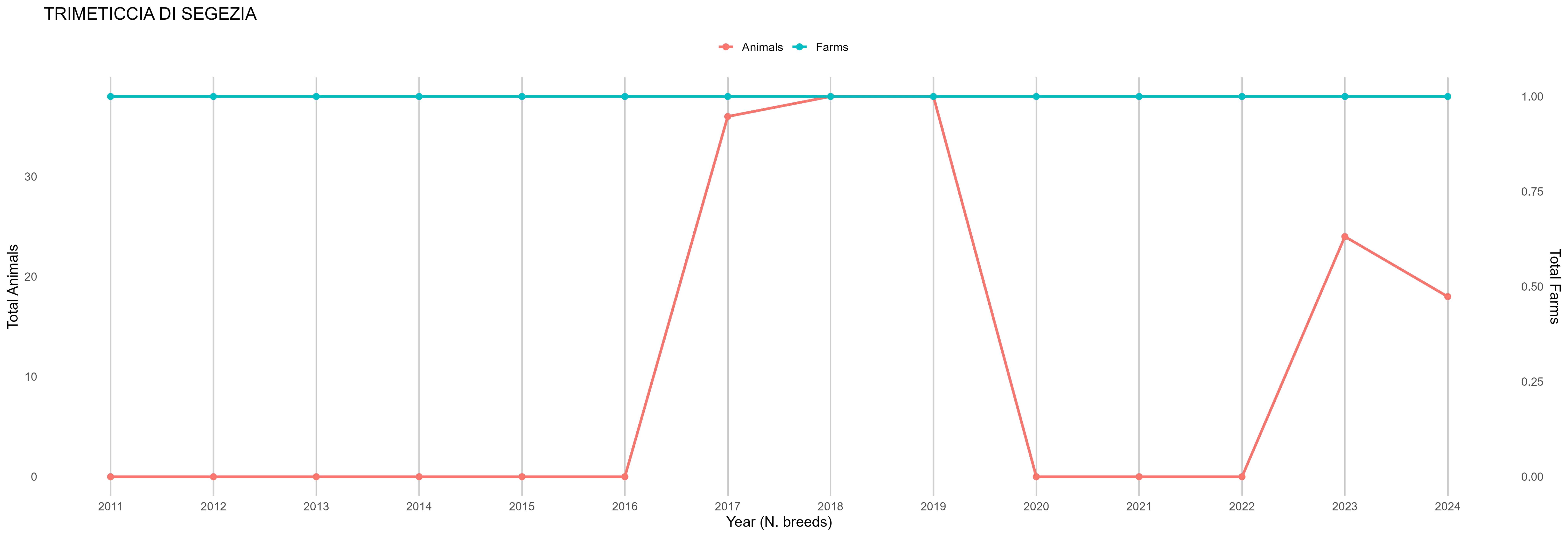

TRIMETICCIA DI SEGEZIA
Animals
Farms
Total Animals
Total Farms
0
10
20
30
0.00
0.25
0.50
0.75
1.00
2011
2012
2013
2014
2015
2016
2017
2018
2019
2020
2021
2022
2023
2024
Year (N. breeds)

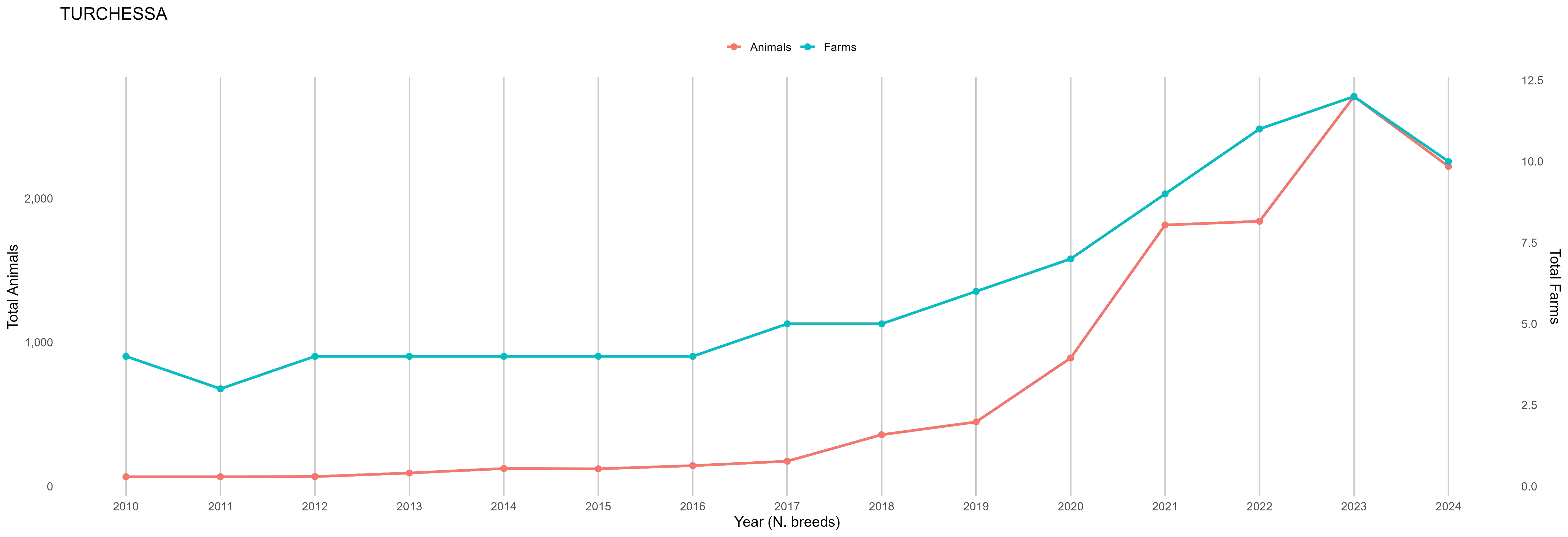
TURCHESSA
Animals
Farms
Total Animals
Total Farms
Year (N. breeds)
0
1,000
2,000
0.0
2.5
5.0
7.5
10.0
12.5
2010
2011
2012
2013
2014
2015
2016
2017
2018
2019
2020
2021
2022
2023
2024

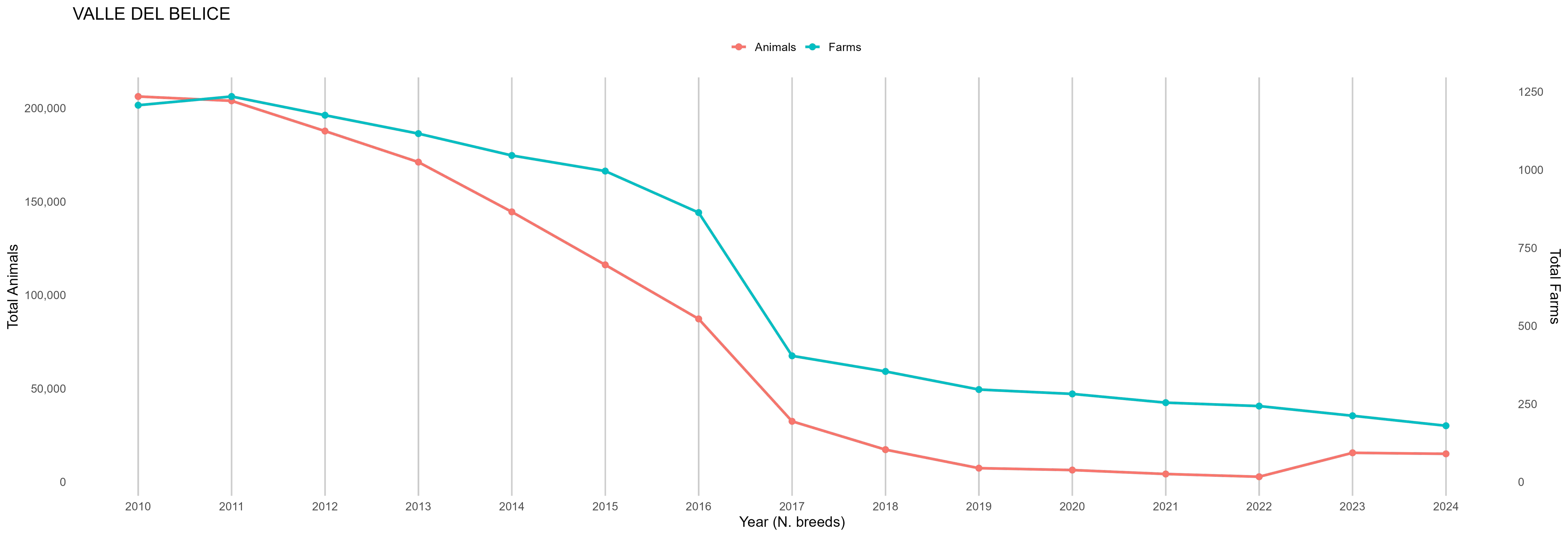
VALLE DEL BELICE
Animals
Farms
200,000
150,000
100,000
50,000
0
Total Animals
1250
1000
750
500
250
0
Total Farms
2010
2011
2012
2013
2014
2015
2016
2017
2018
2019
2020
2021
2022
2023
2024
Year (N. breeds)

VICENTINA-FOZA

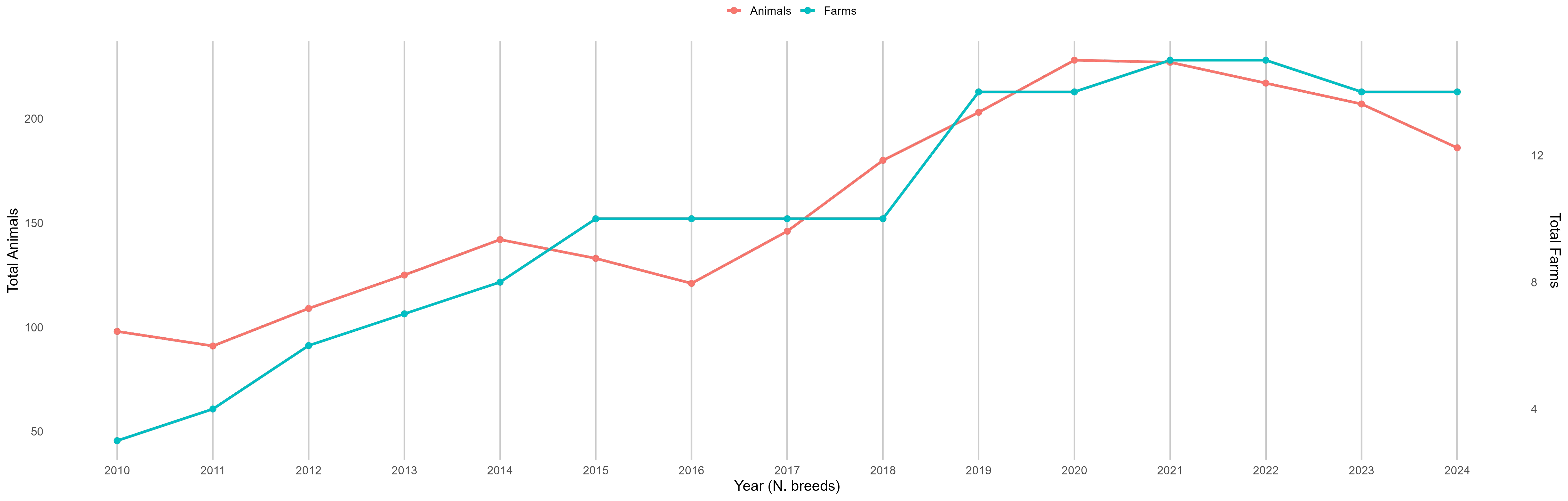

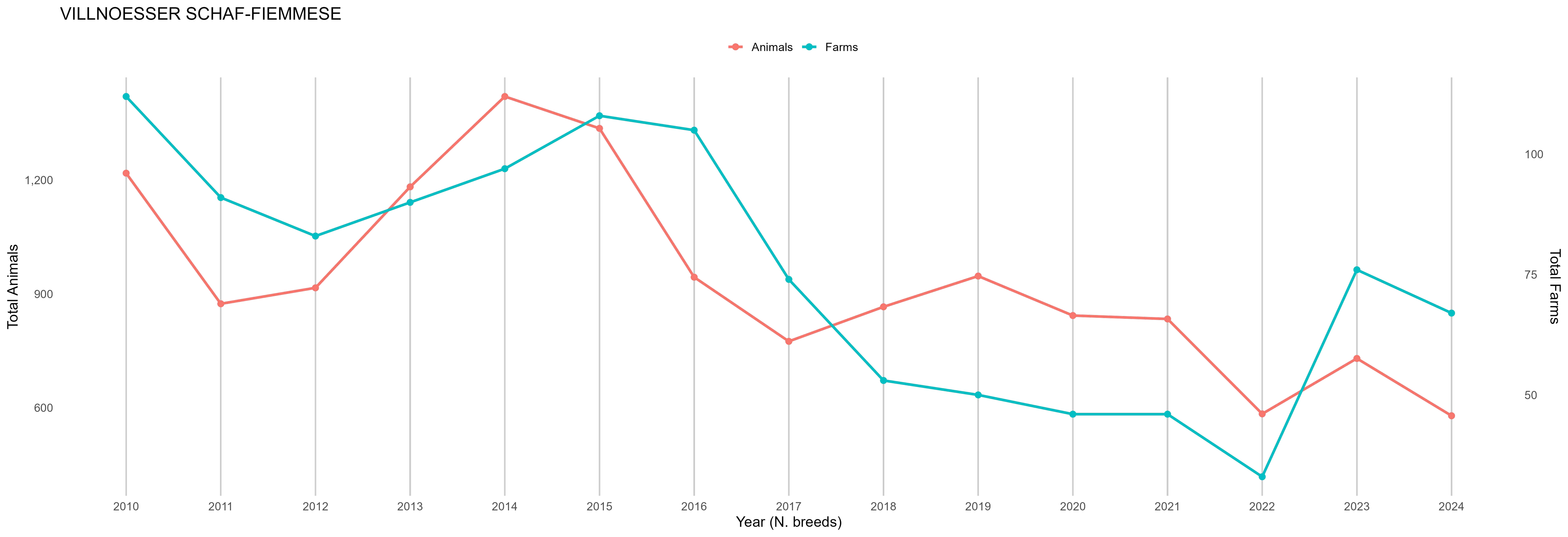

VILLNOESSER SCHAF-FIEMMESE
Animals
Farms
Total Animals
1,200
900
600
Total Farms
100
75
50
2010
2011
2012
2013
2014
2015
2016
2017
2018
2019
2020
2021
2022
2023
2024
Year (N. breeds)

ZERASCA

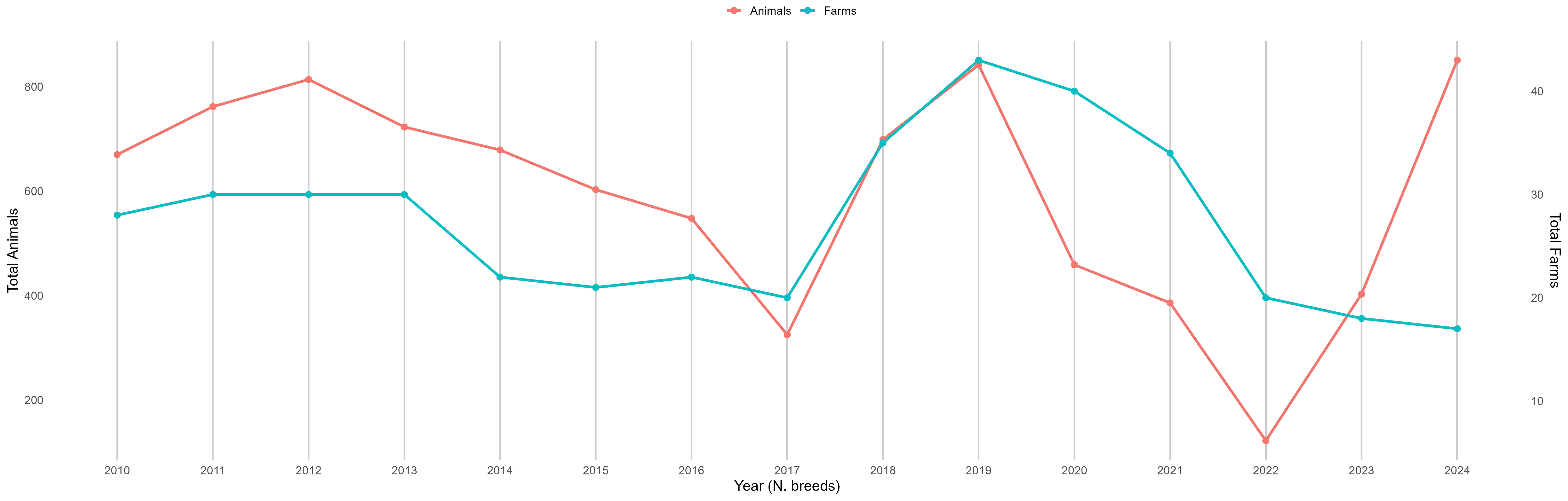

Animals
Farms
Total Animals
Total Farms
800
600
400
200
40
30
20
10
2010
2011
2012
2013
2014
2015
2016
2017
2018
2019
2020
2021
2022
2023
2024
Year (N. breeds)
