# Supplementary figures and images for: Genomic insights into the recent evolution and biodiversity of Italian sheep breeds
Source: Mamm Genome. 2025 Nov 22;37(1):5. doi: 10.1007/s00335-025-10170-8 (PMC12640353; doi:10.1007/s00335-025-10170-8)

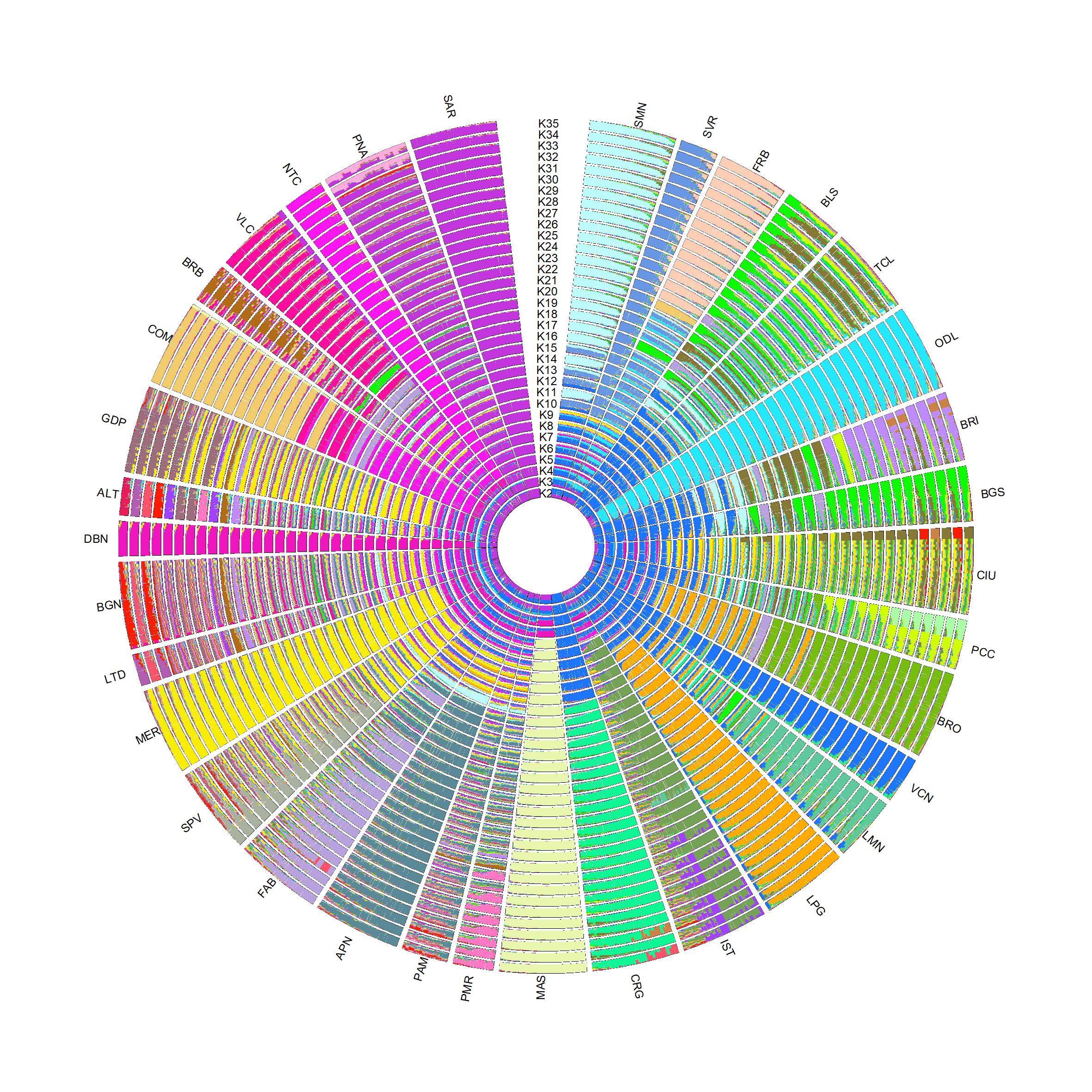

Supplement: Supplementary file 2 — Supplementary Fig. S2 Admixture analysis for a number of clusters (K) ranging from 2 to 35. (PNG 499 kb) [file 335_2025_10170_MOESM2_ESM.png]

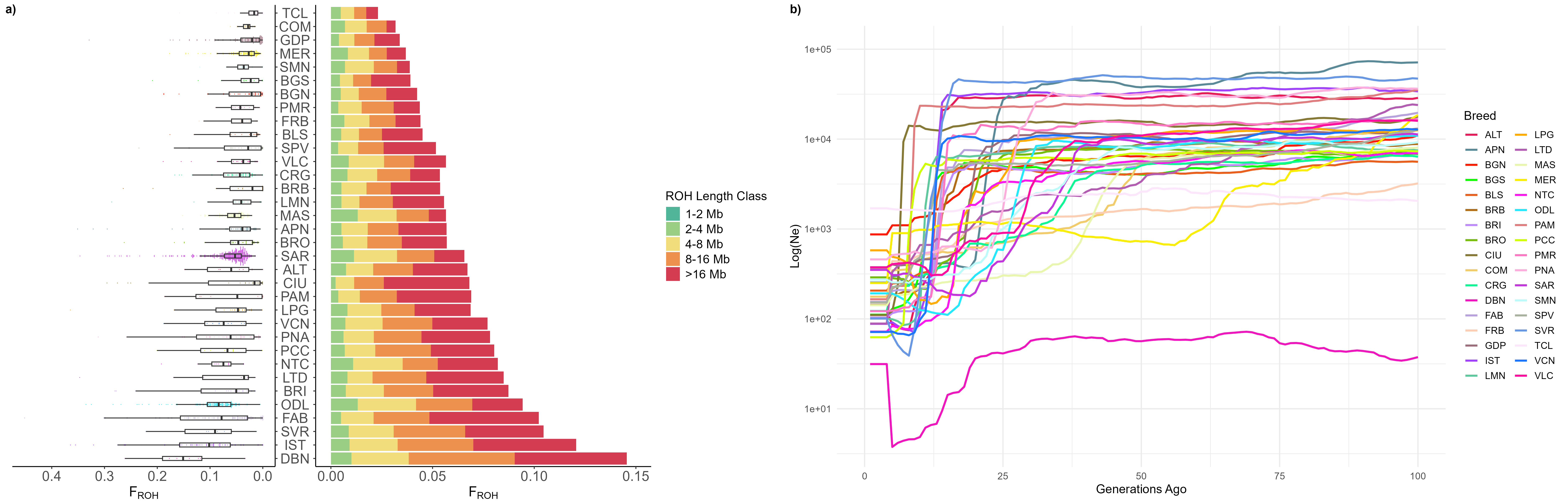

Supplement: Supplementary file 3 — Supplementary Fig. S3 Genetic diversity of Italian sheep breeds. a) Boxplot of individual inbreeding coefficients based on runs of homozygosity (FROH). Each point corresponds to one individual. Boxplots show the median (line), interquartile range (IQR, box), whiskers extending up to 1.5 × IQR, and points beyond whiskers representing outliers. b) Barplot of mean FROH per breed. Colors indicate the contribution of ROH of different length classes. Breeds are ordered by total mean FROH. (PNG 1156 kb) [file 335_2025_10170_MOESM3_ESM.png]

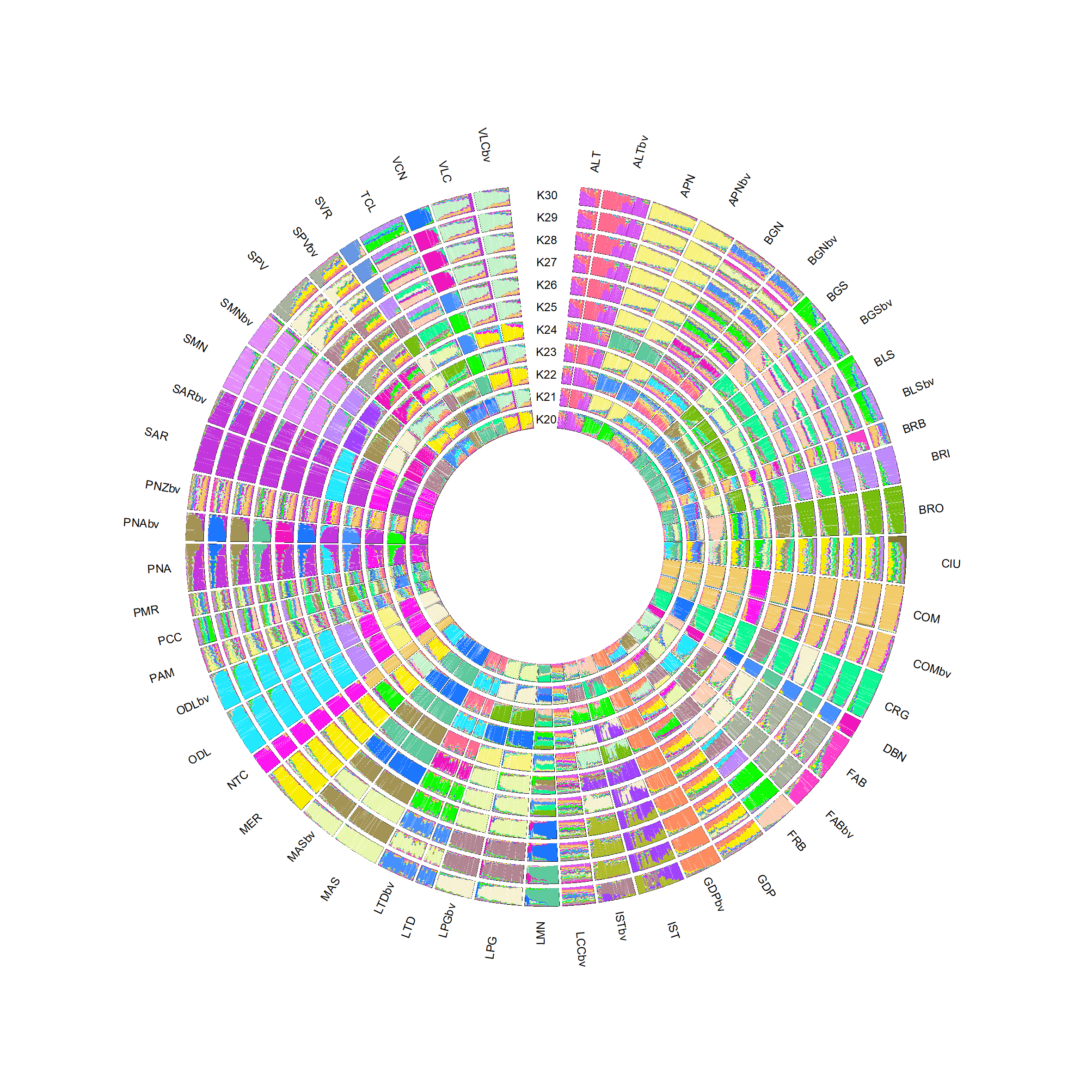

Supplement: Supplementary file 4 — Supplementary Fig. S4 Admixture analysis for a number of clusters (K) ranging from 20 to 30, including recent and older (codes including ‘bv’) samples. (PNG 383 kb) [file 335_2025_10170_MOESM4_ESM.png]
