# Supplementary material for: Genomic insights into the recent evolution and biodiversity of Italian sheep breeds
Source: Mamm Genome. 2025 Nov 22;37(1):5. doi: 10.1007/s00335-025-10170-8 (PMC12640353; doi:10.1007/s00335-025-10170-8)
Supplement: Supplementary file 5 — Supplementary Fig. S5 Local ancestry inference with Gentile di Puglia (GDP) as the target population, older GDP samples (GDPbv) as the background, and Merinizzata Italiana (MER) as the candidate source of introgression. (PDF 584 kb) [file 335_2025_10170_MOESM5_ESM.pdf]

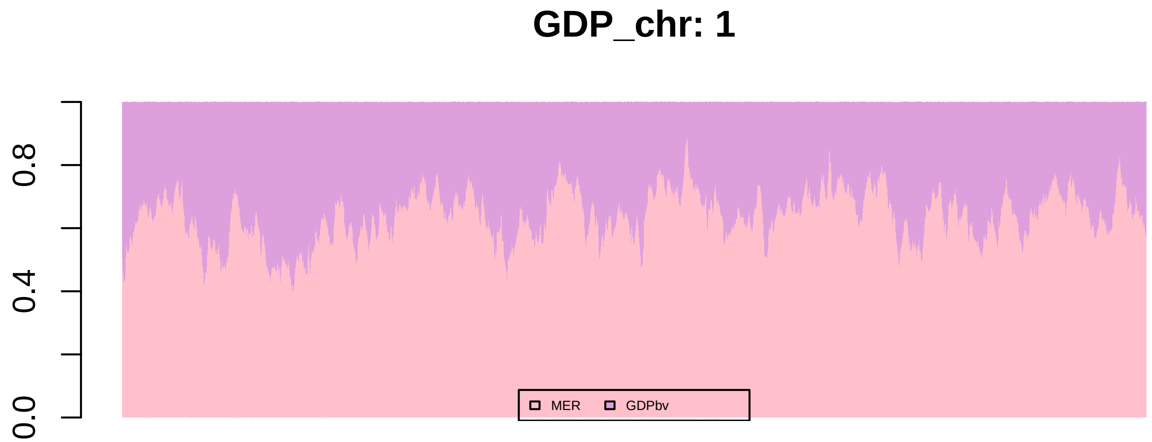
GDP_chr: 1
0.0
0.4
0.8
MER
GDPbv

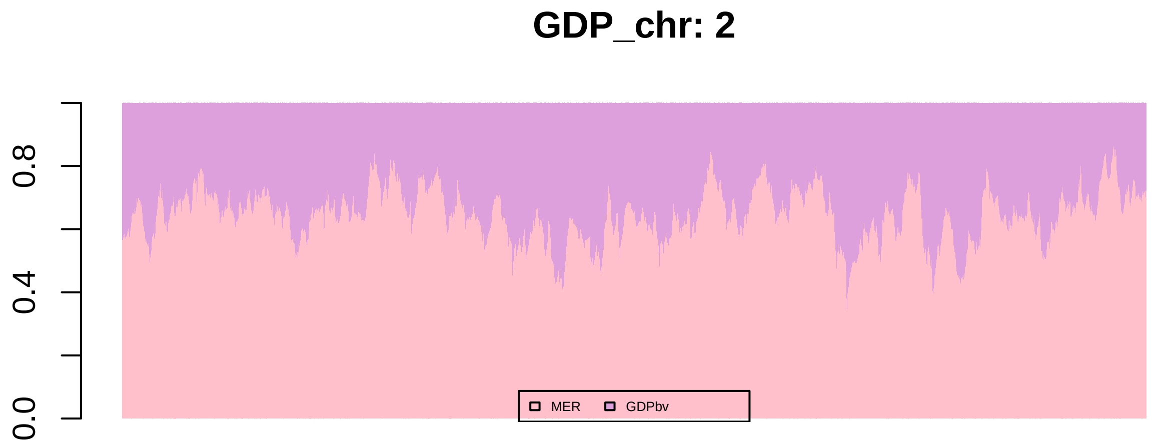
GDP_chr: 2
0.0
0.4
0.8
MER
GDPbv

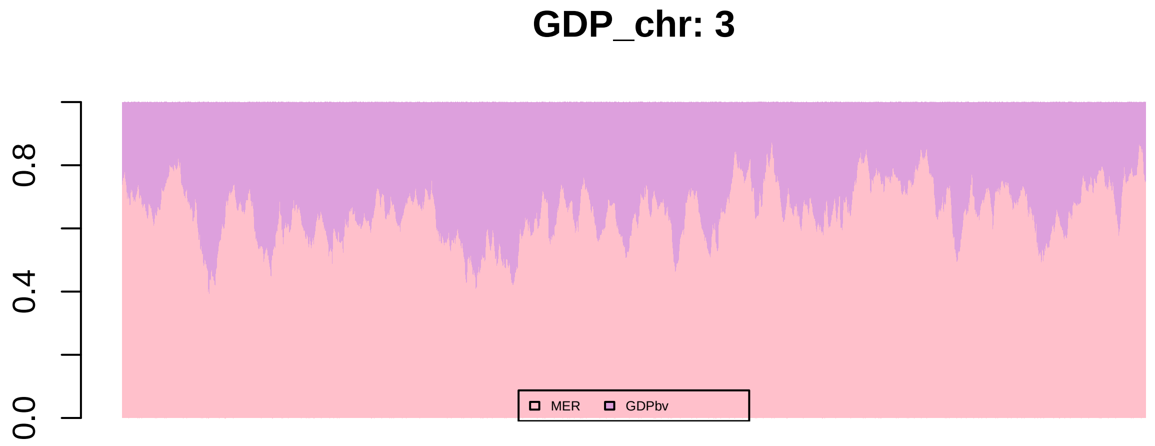

GDP_chr: 3
0.0
0.4
0.8
MER
GDPbv

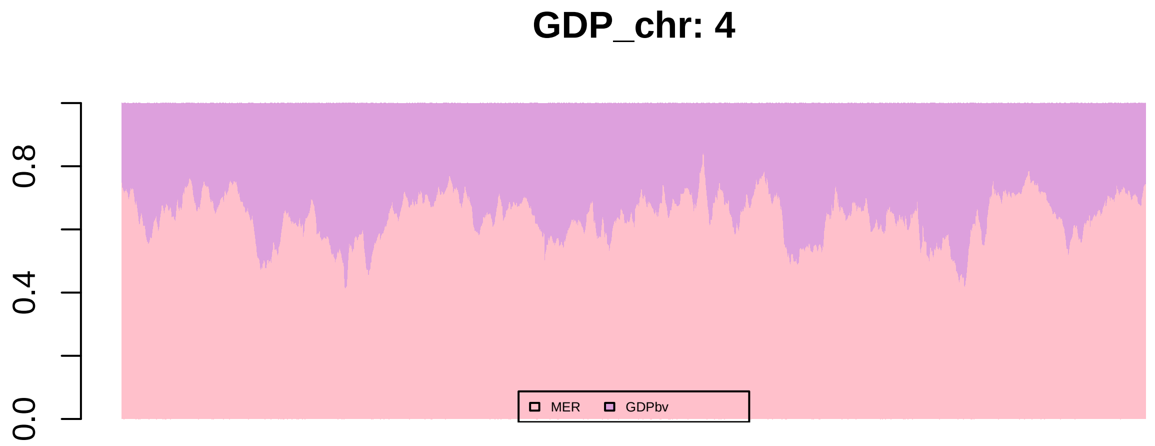

GDP_chr: 4
0.0
0.4
0.8
MER
GDPbv

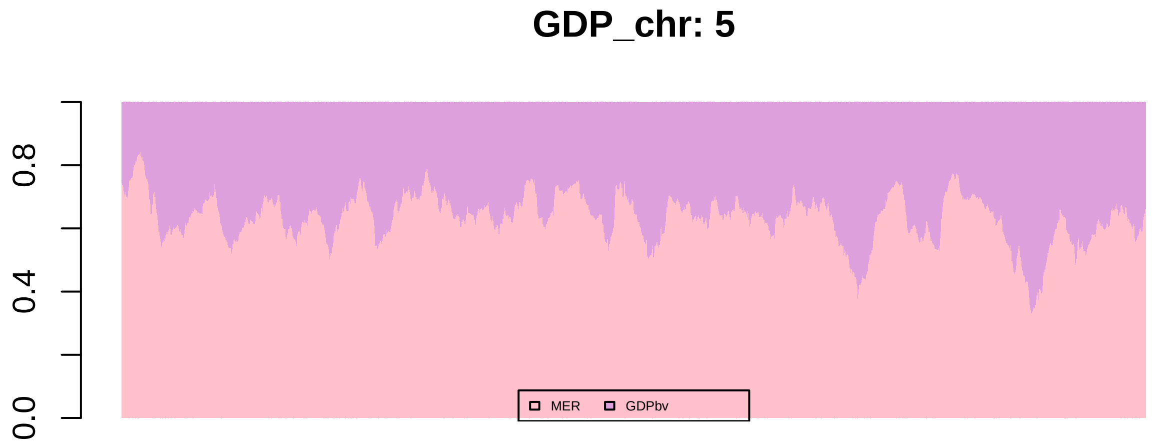
GDP_chr: 5
0.0
0.4
0.8
MER
GDPbv

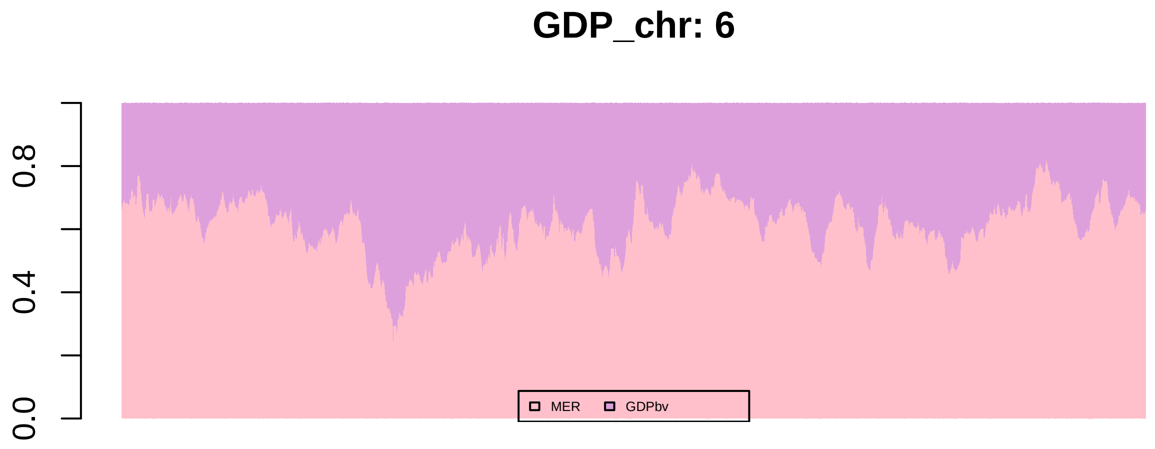
GDP_chr: 6
0.0
0.4
0.8
MER
GDPbv

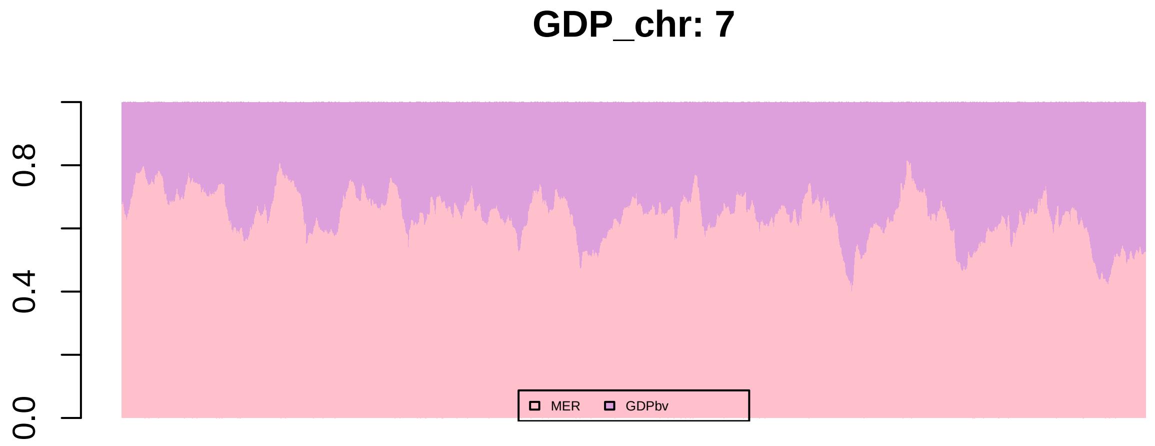
GDP_chr: 7
0.0
0.4
0.8
MER
GDPbv

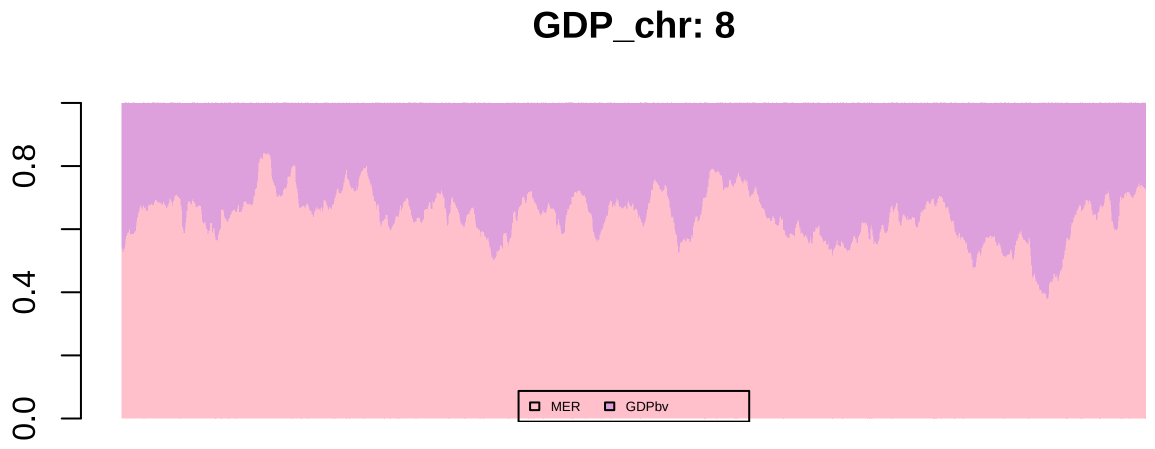
GDP_chr: 8
0.0
0.4
0.8
MER
GDPbv

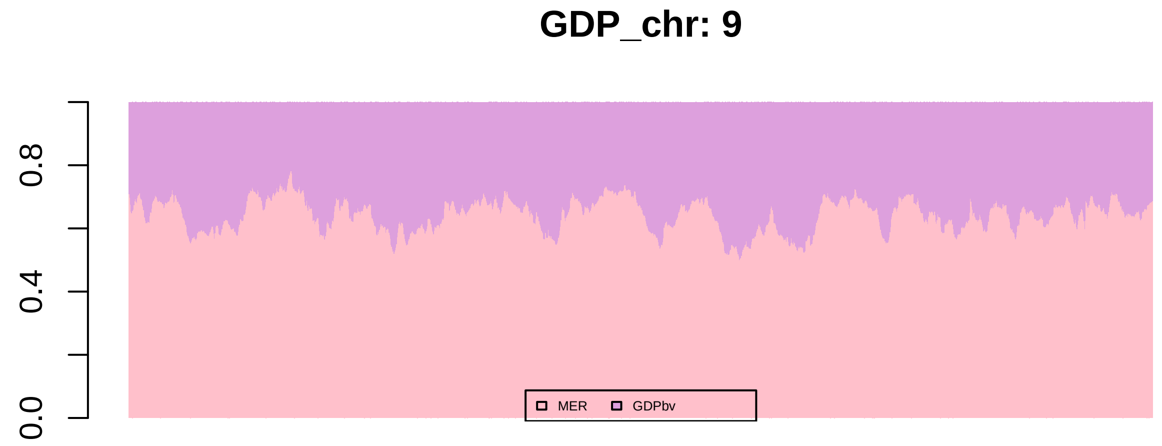
GDP_chr: 9
0.0
0.4
0.8
MER
GDPbv

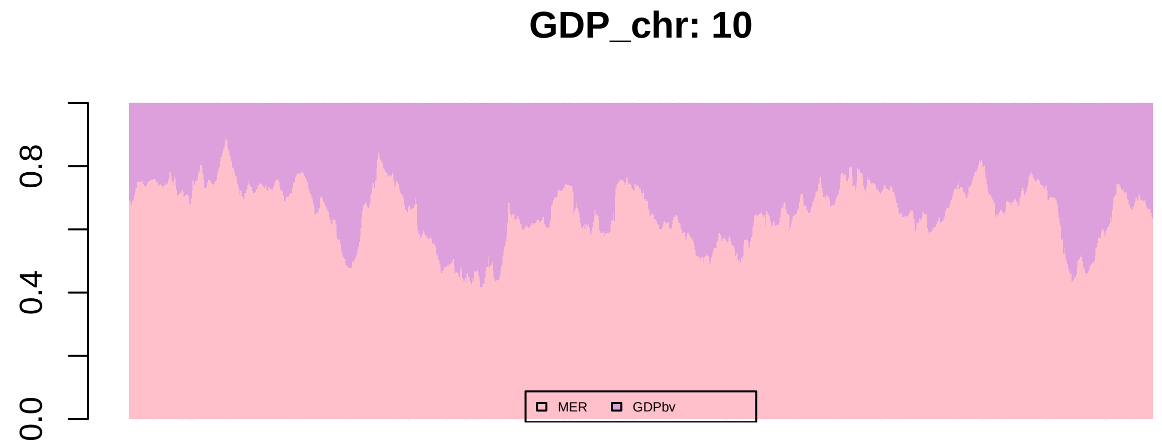
GDP_chr: 10
0.0
0.4
0.8
MER
GDPbv

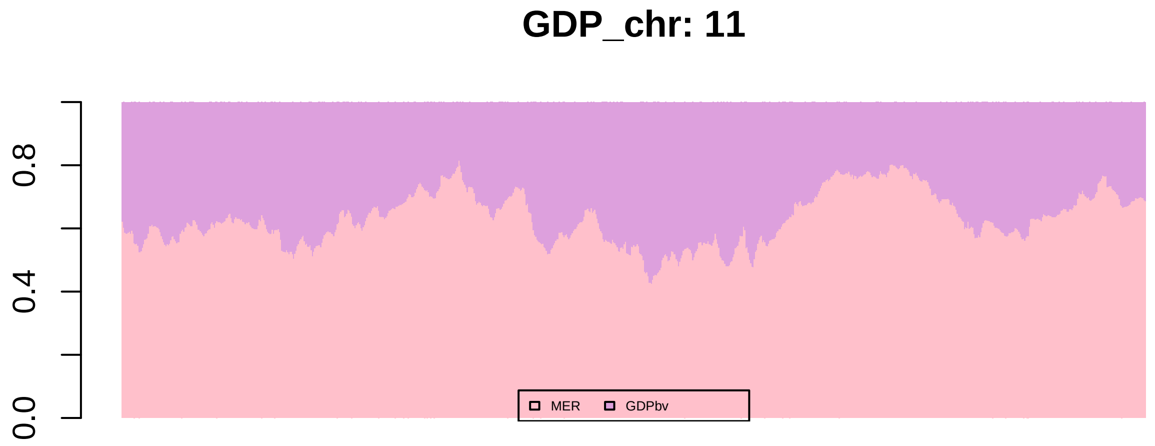
GDP_chr: 11
0.0
0.4
0.8
MER
GDPbv

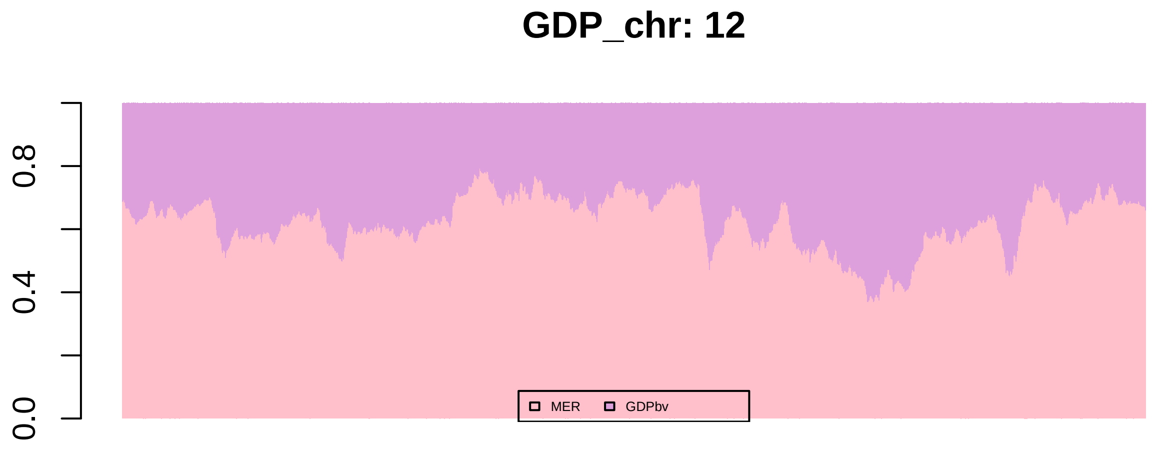
GDP_chr: 12
0.0
0.4
0.8
MER
GDPbv

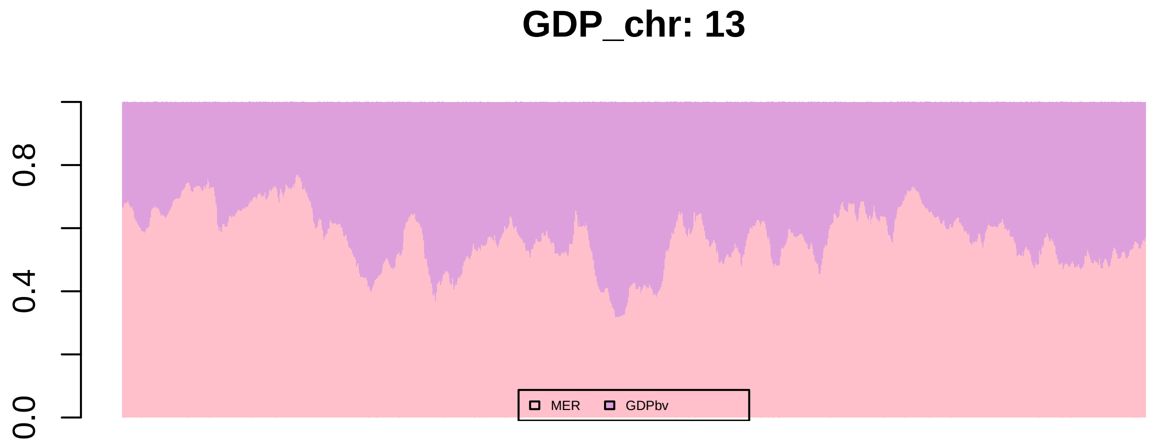

GDP_chr: 13
0.0
0.4
0.8
MER
GDPbv

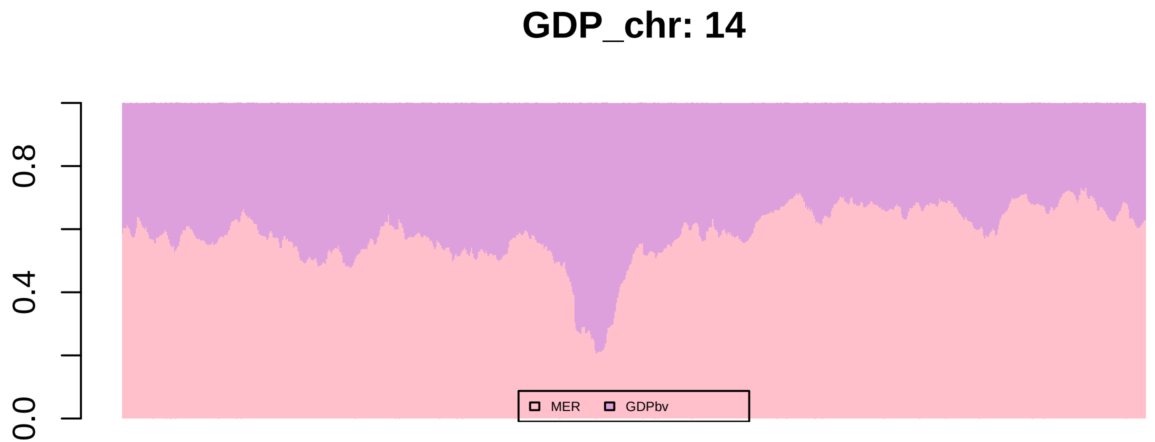

GDP_chr: 14
0.0
0.4
0.8
MER
GDPbv

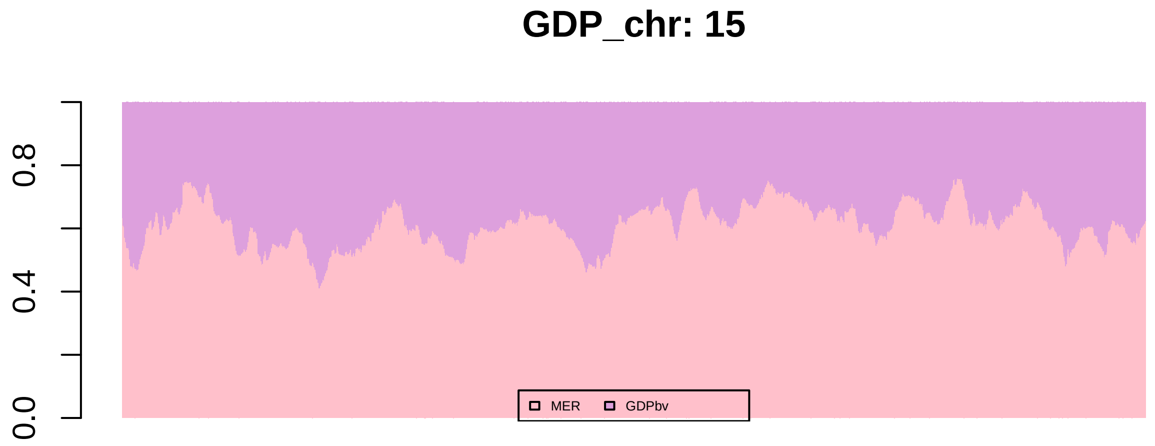
GDP_chr: 15
0.0
0.4
0.8
MER
GDPbv

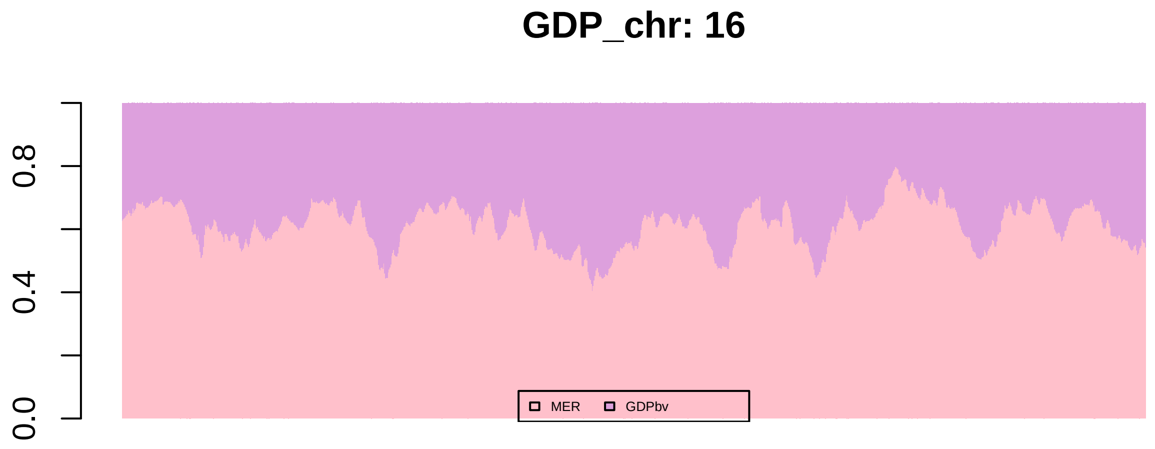
GDP_chr: 16
0.0
0.4
0.8
MER
GDPbv

GDP_chr: 17

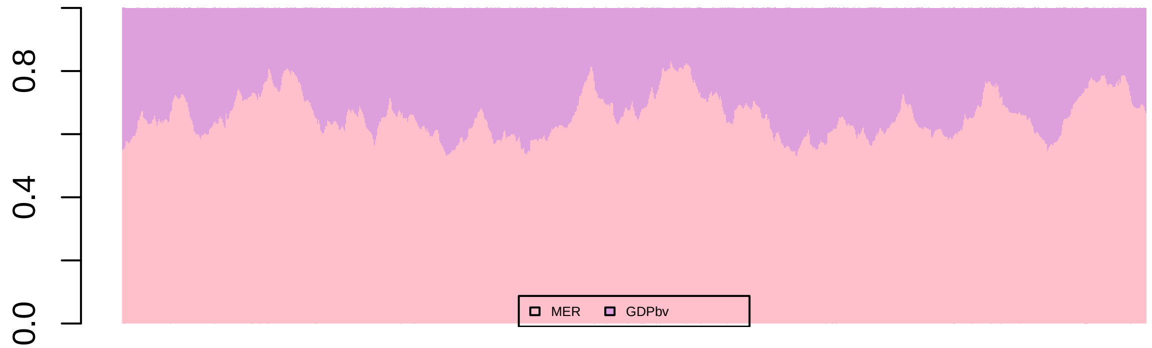

GDP_chr: 18

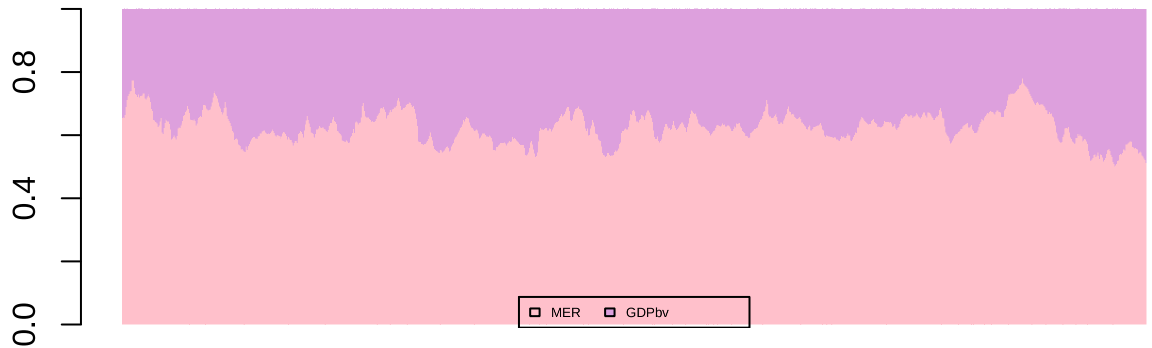

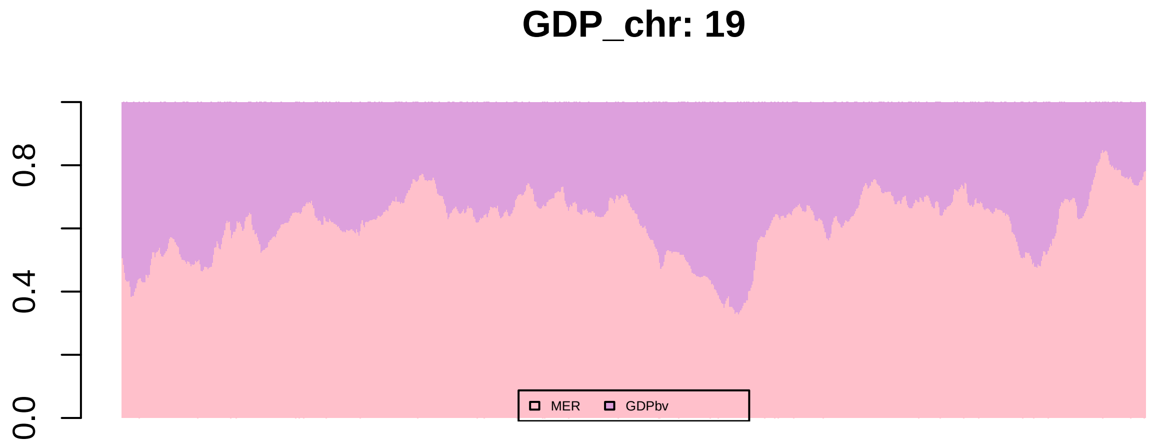
GDP_chr: 19
0.0
0.4
0.8
MER
GDPbv

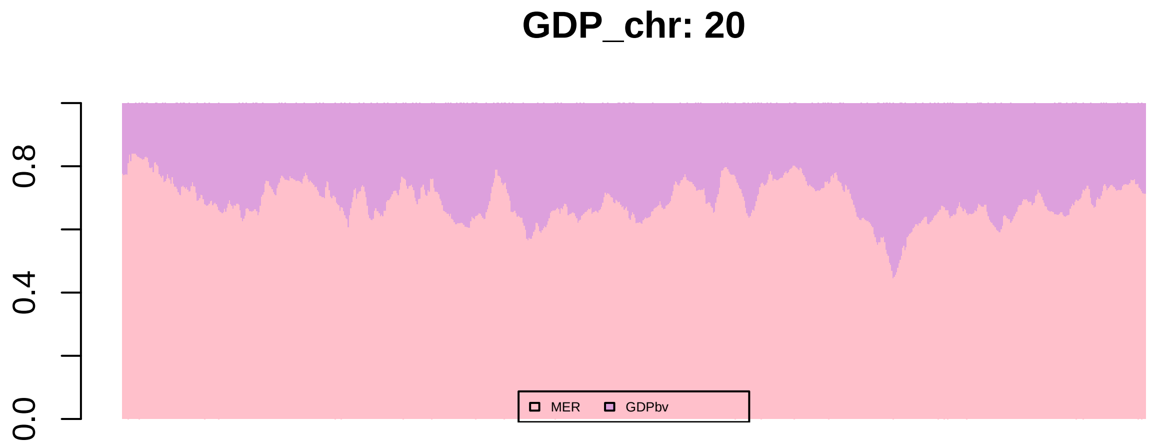
GDP_chr: 20
0.0
0.4
0.8
MER
GDPbv

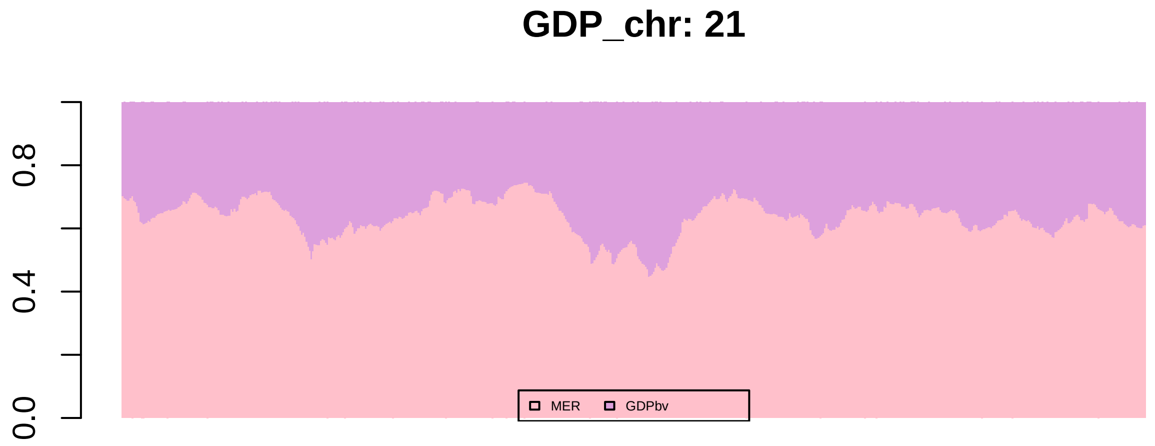
GDP_chr: 21
0.0
0.4
0.8
MER
GDPbv

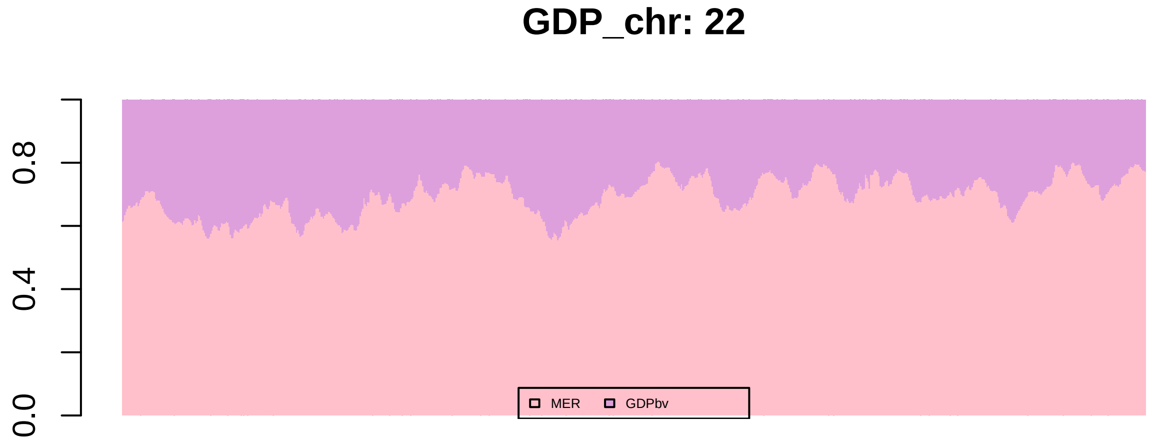
GDP_chr: 22
0.0
0.4
0.8
MER
GDPbv

GDP_chr: 23

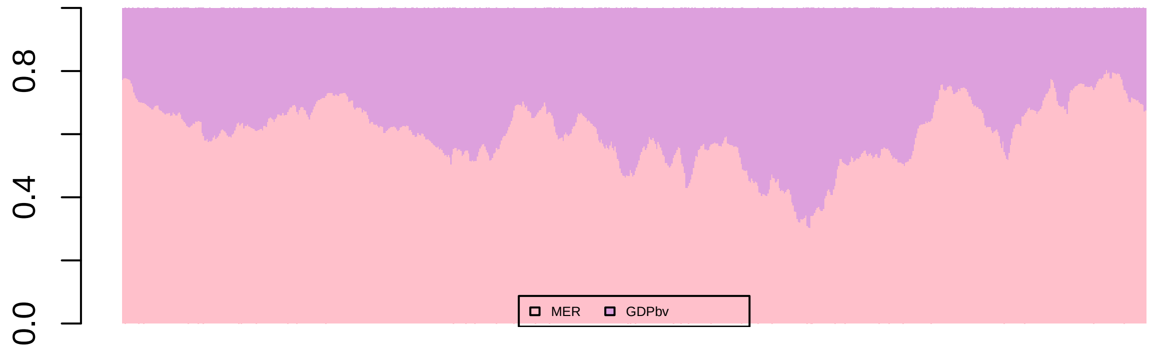

GDP_chr: 24

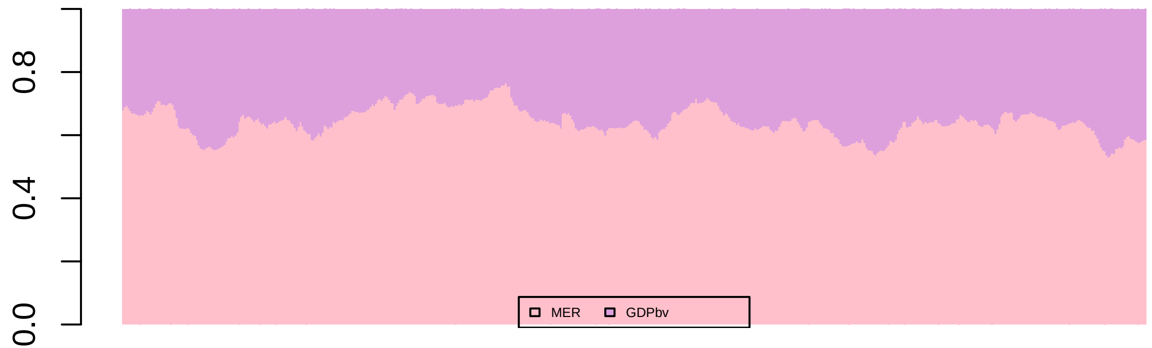

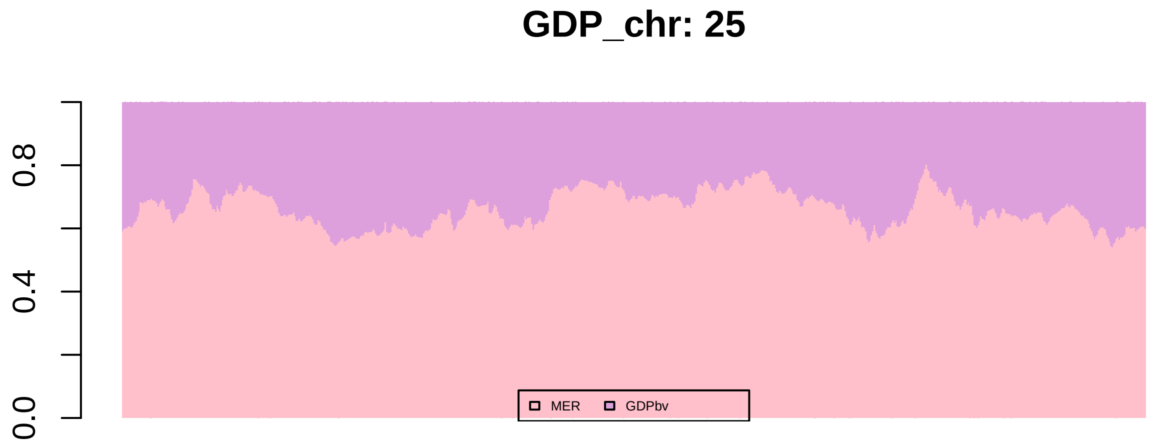
GDP_chr: 25
0.0
0.4
0.8
MER
GDPbv

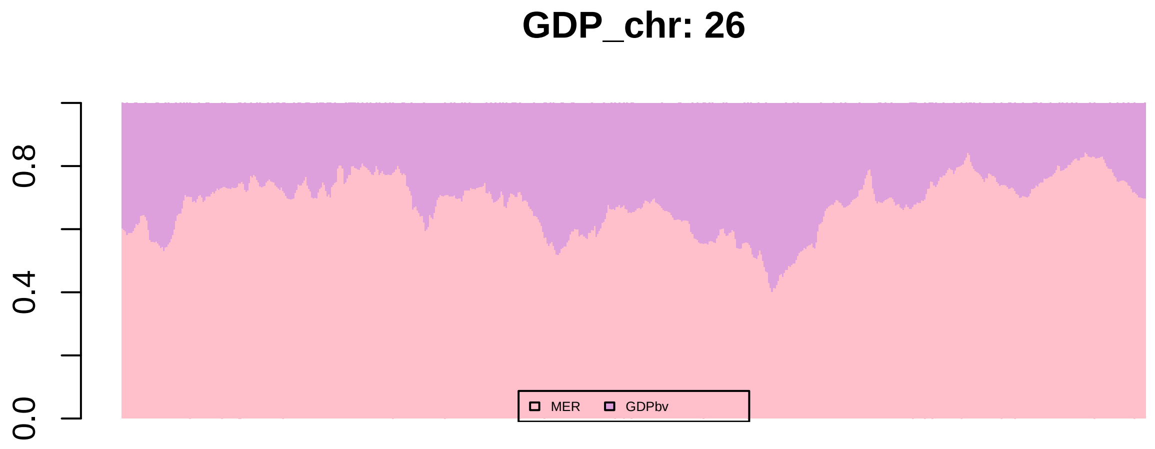
GDP_chr: 26
0.0
0.4
0.8
MER
GDPbv
