# Supplementary material for: Genomic insights into the recent evolution and biodiversity of Italian sheep breeds
Source: Mamm Genome. 2025 Nov 22;37(1):5. doi: 10.1007/s00335-025-10170-8 (PMC12640353; doi:10.1007/s00335-025-10170-8)
Supplement: Supplementary file 6 — Supplementary Fig. S6 Local ancestry inference with Nera di Arbus (PNA) as the target population, older PNA samples (PNAbv) as the background, and Sarda (SAR) as the candidate source of introgression. (PDF 589 kb) [file 335_2025_10170_MOESM6_ESM.pdf]

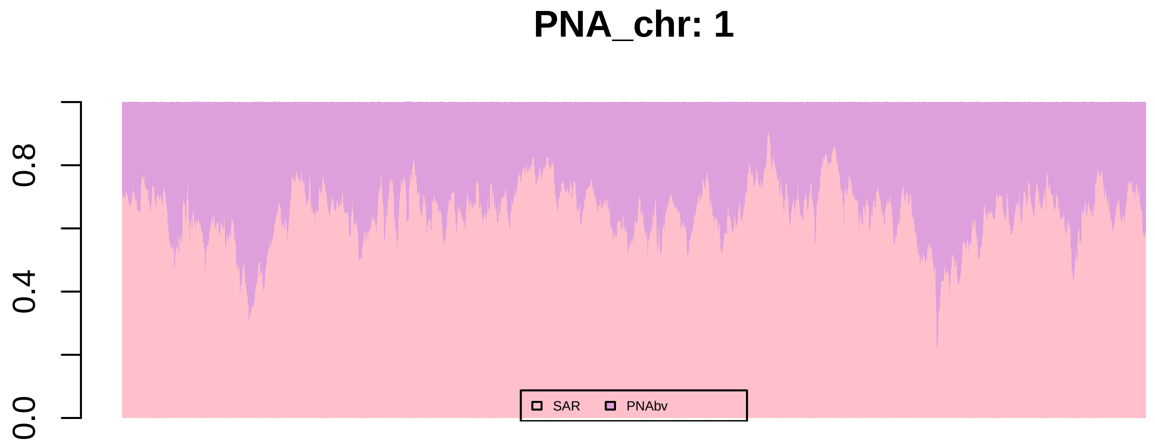
PNA_chr: 1
0.0
0.4
0.8
SAR
PNAbv

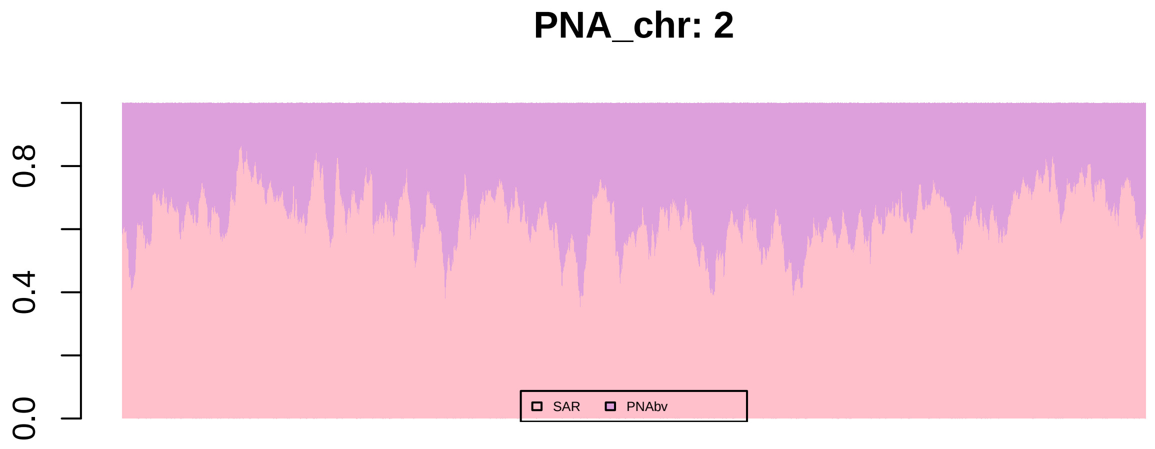
PNA_chr: 2
0.0
0.4
0.8
SAR
PNAbv

PNA_chr: 3

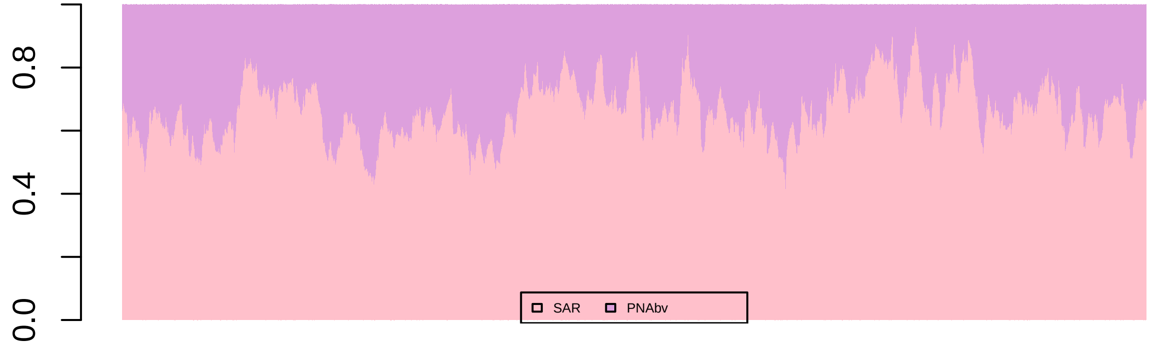

PNA_chr: 4

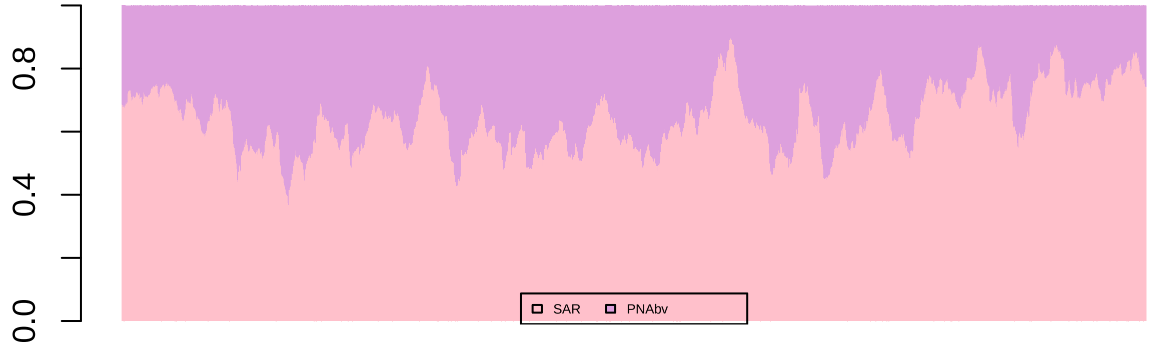

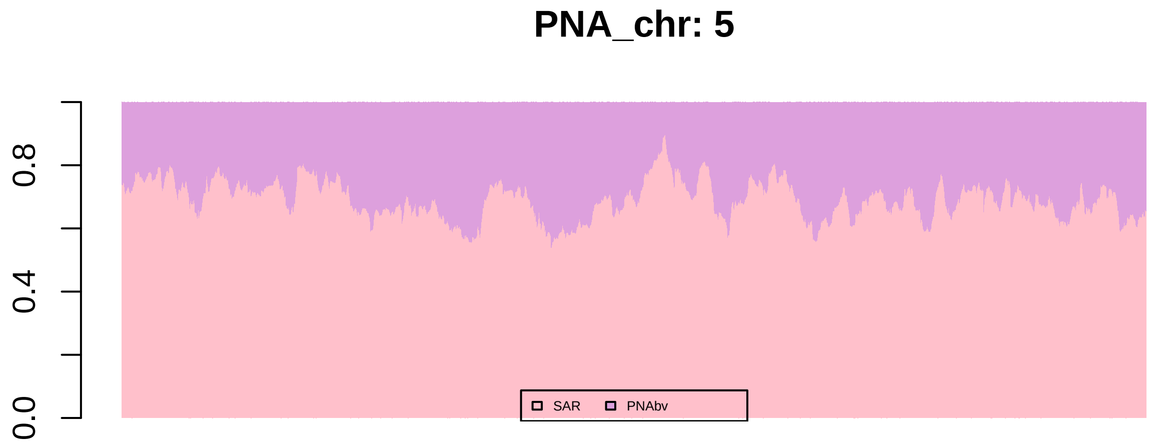
PNA_chr: 5
0.0
0.4
0.8
SAR
PNAbv

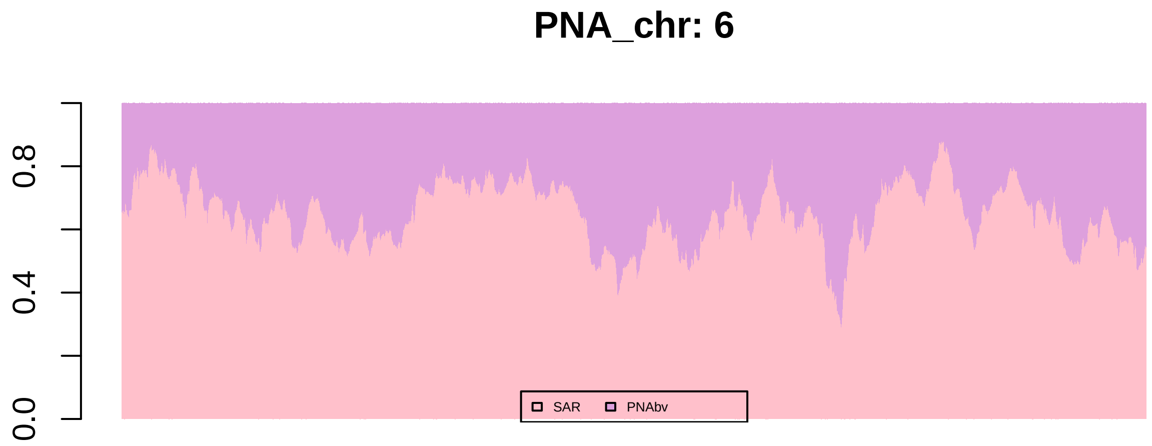
PNA_chr: 6
0.0
0.4
0.8
SAR
PNAbv

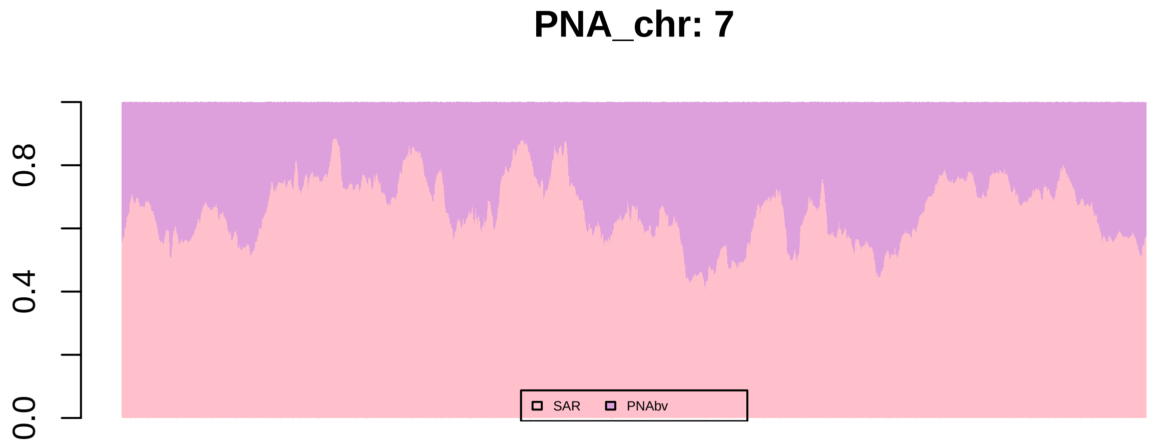
PNA_chr: 7
0.0
0.4
0.8
SAR
PNAbv

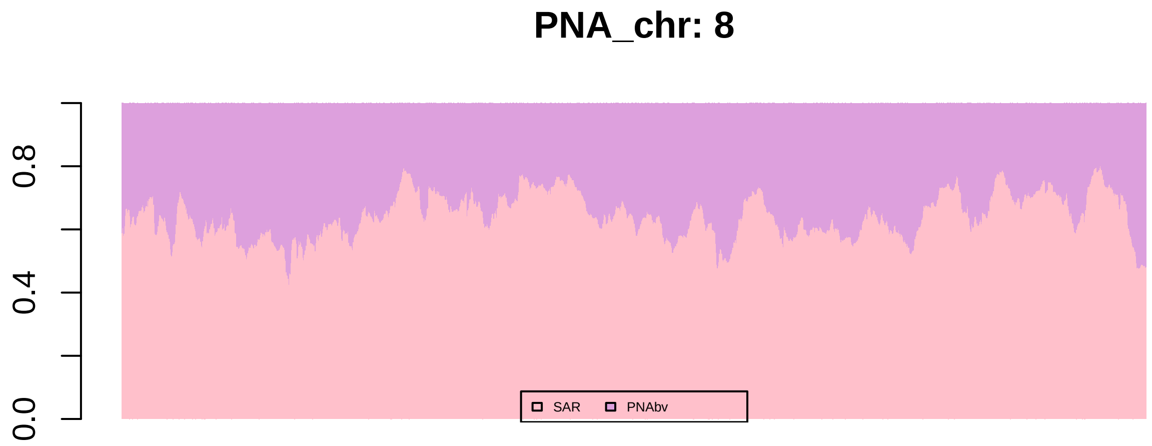
PNA_chr: 8
0.0
0.4
0.8
SAR
PNAbv

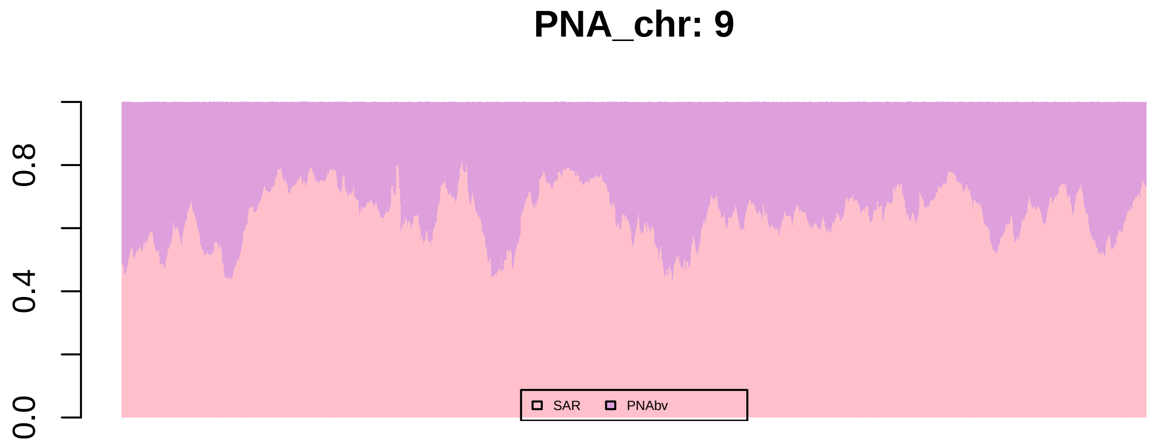
PNA_chr: 9
0.0
0.4
0.8
SAR
PNAbv

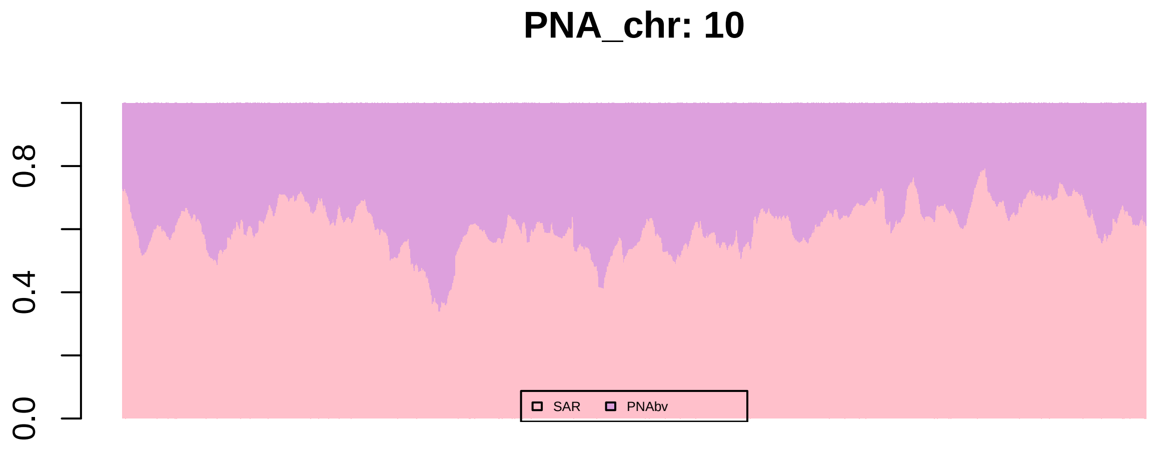
PNA_chr: 10
0.0
0.4
0.8
SAR
PNAbv

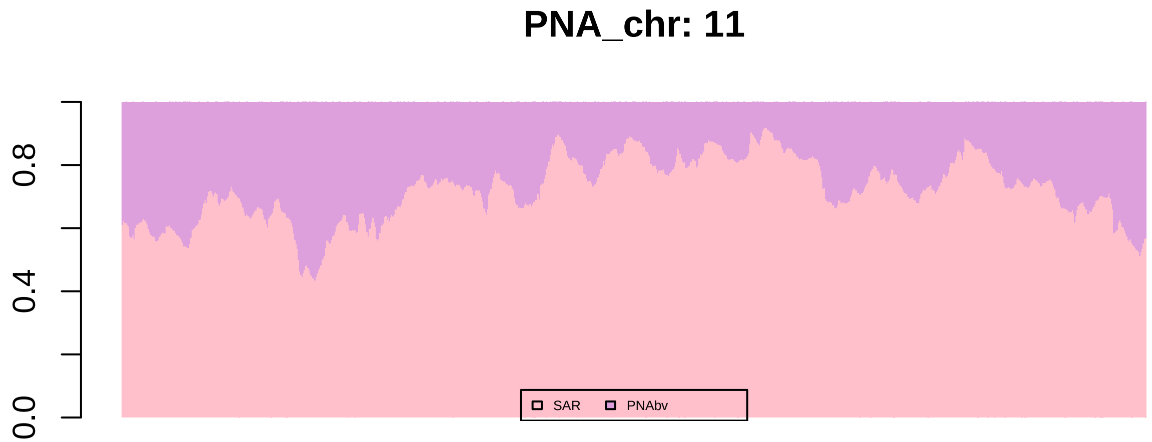
PNA_chr: 11
0.0
0.4
0.8
SAR
PNAbv

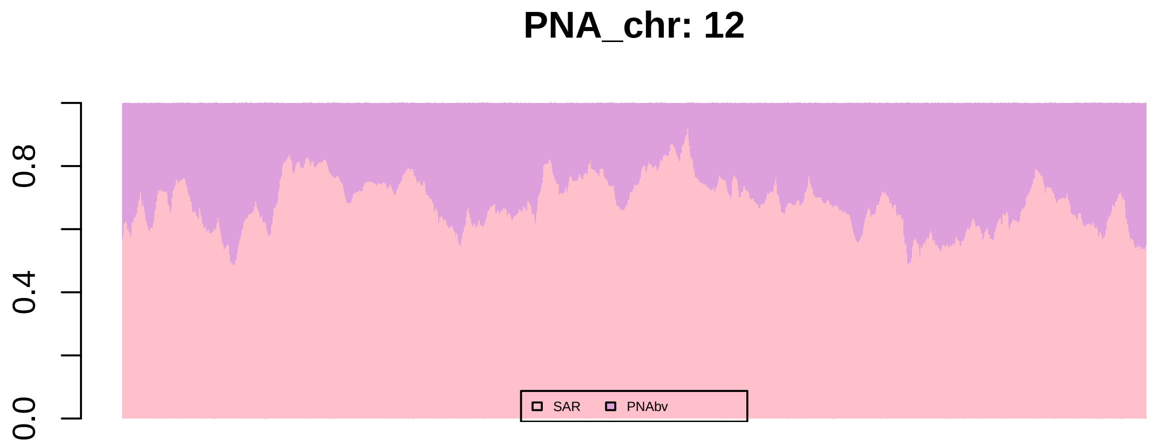
PNA_chr: 12
0.0
0.4
0.8
SAR
PNAbv

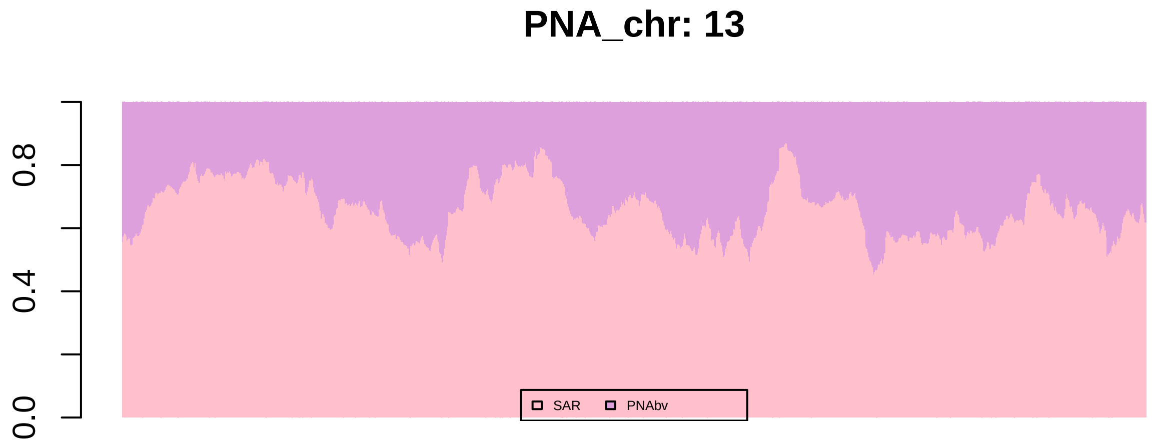
PNA_chr: 13
0.0
0.4
0.8
SAR
PNAbv

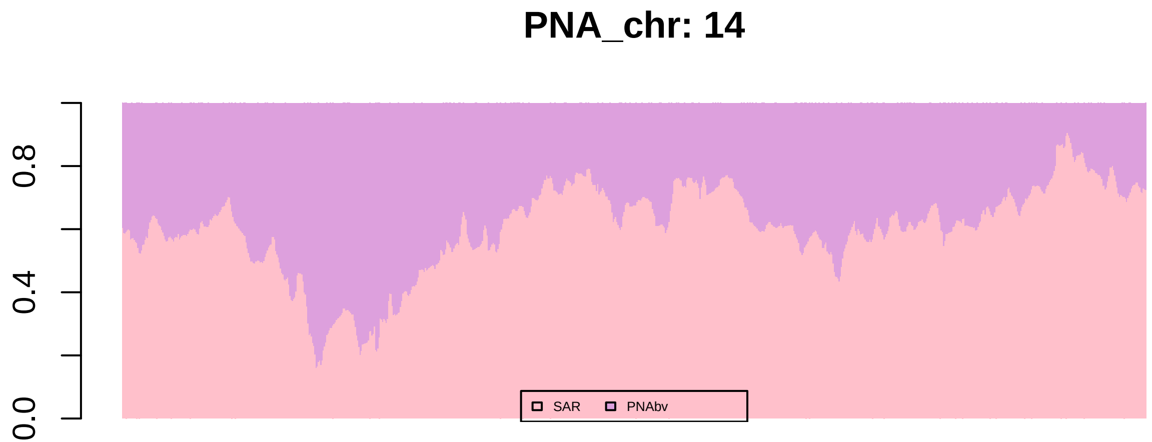
PNA_chr: 14
0.0
0.4
0.8
SAR
PNAbv

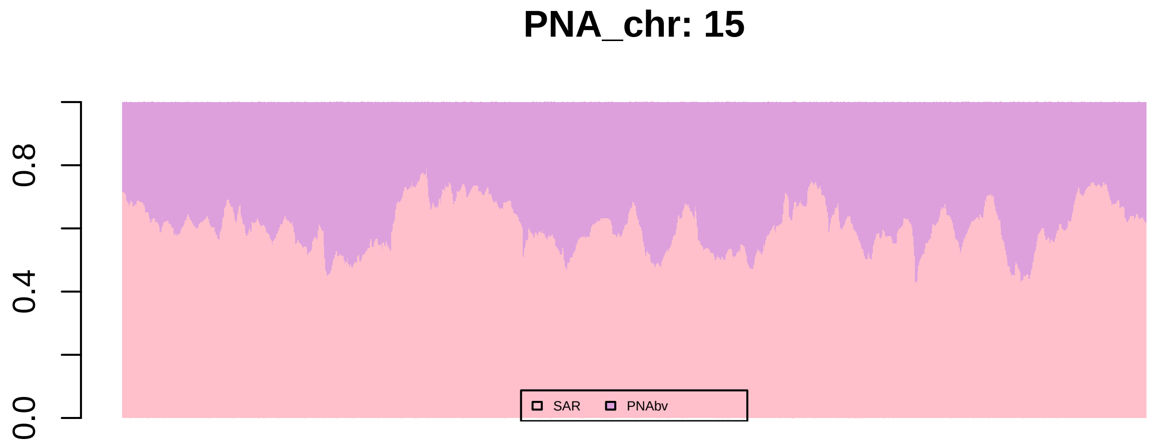
PNA_chr: 15
0.0
0.4
0.8
SAR
PNAbv

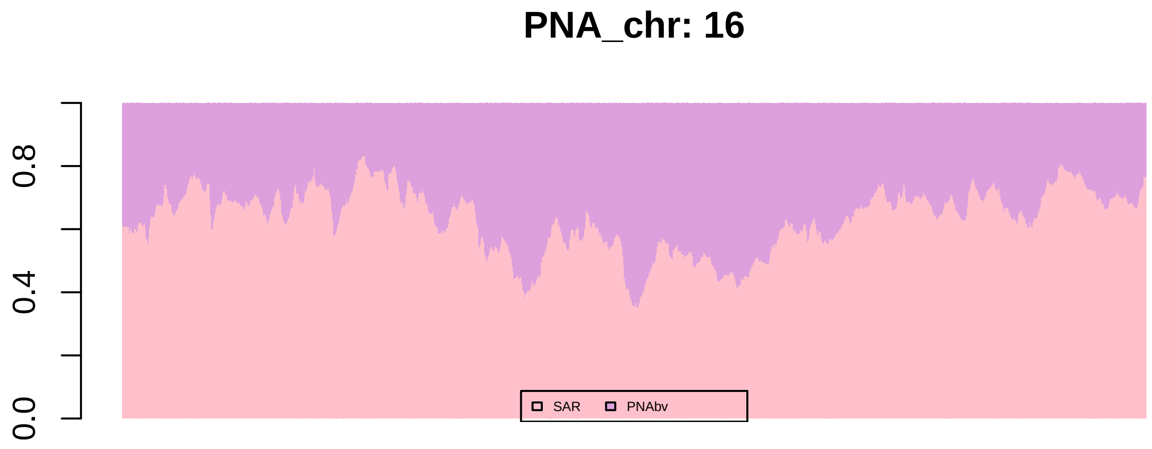
PNA_chr: 16
0.0
0.4
0.8
SAR
PNAbv

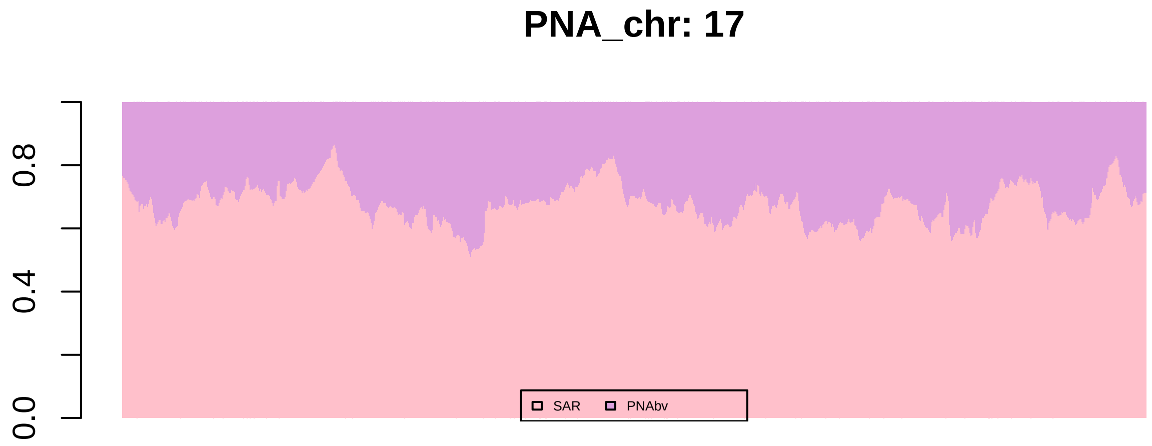
PNA_chr: 17
0.0
0.4
0.8
SAR
PNAbv

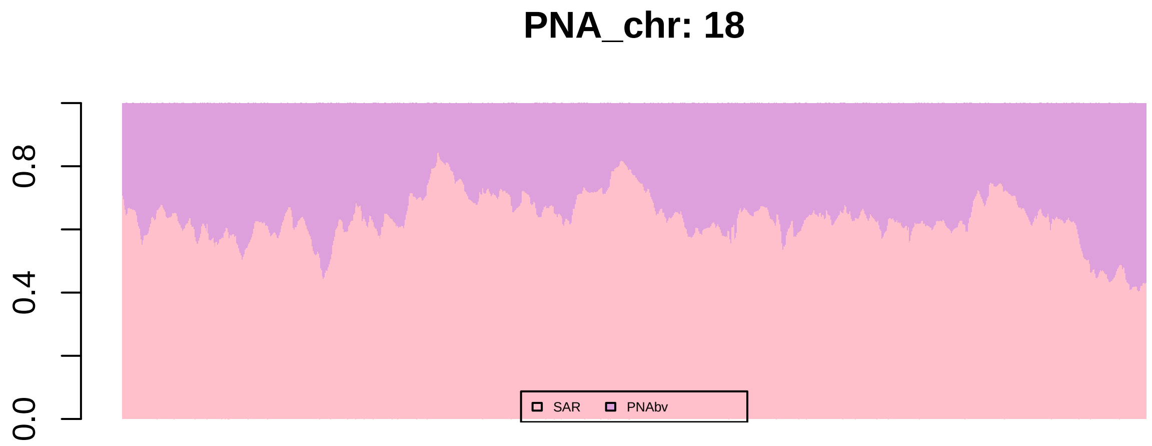
PNA_chr: 18
0.0
0.4
0.8
SAR
PNAbv

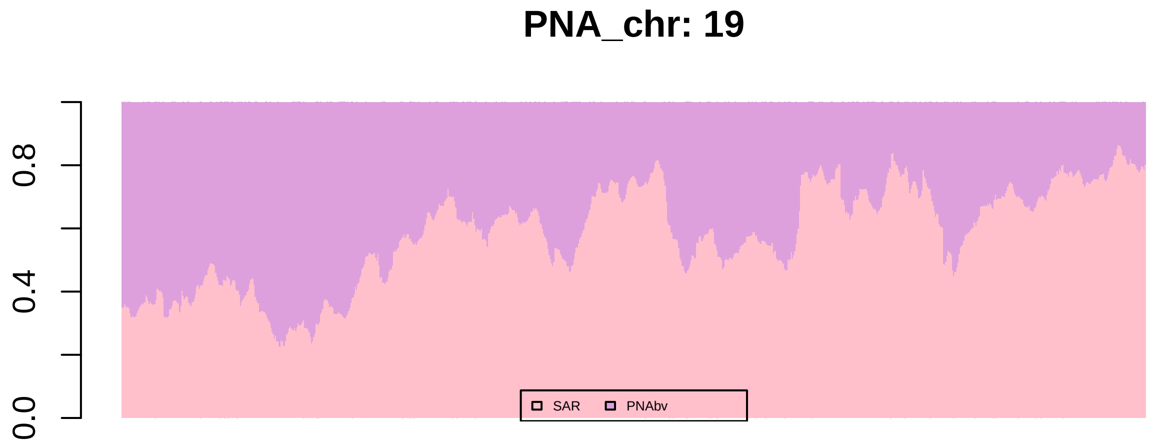
PNA_chr: 19
0.0
0.4
0.8
SAR
PNAbv

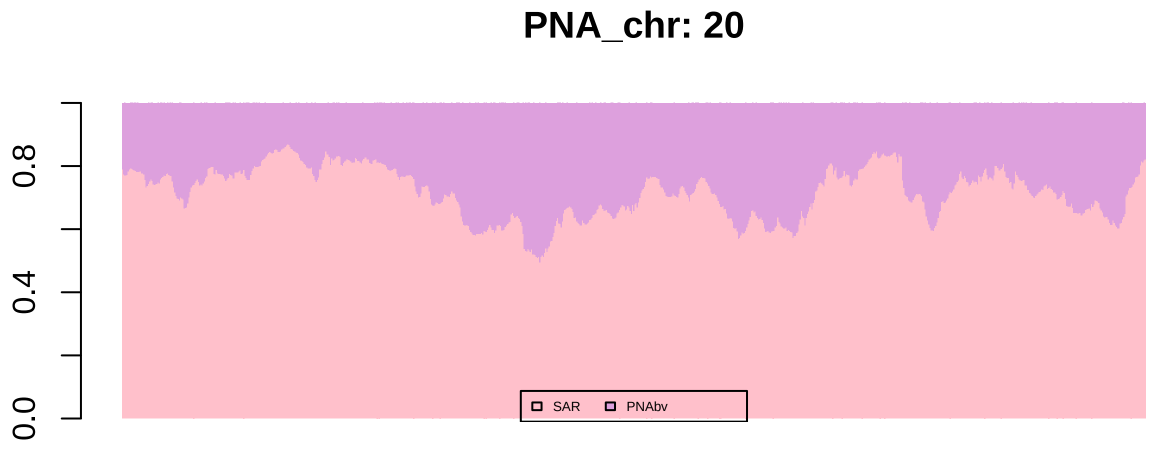
PNA_chr: 20
0.0
0.4
0.8
SAR
PNAbv

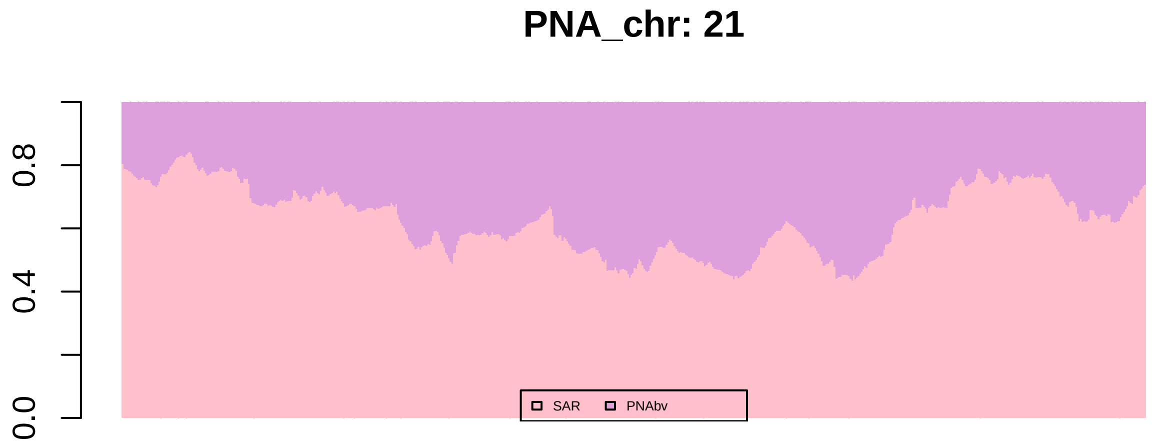
PNA_chr: 21
0.0
0.4
0.8
SAR
PNAbv

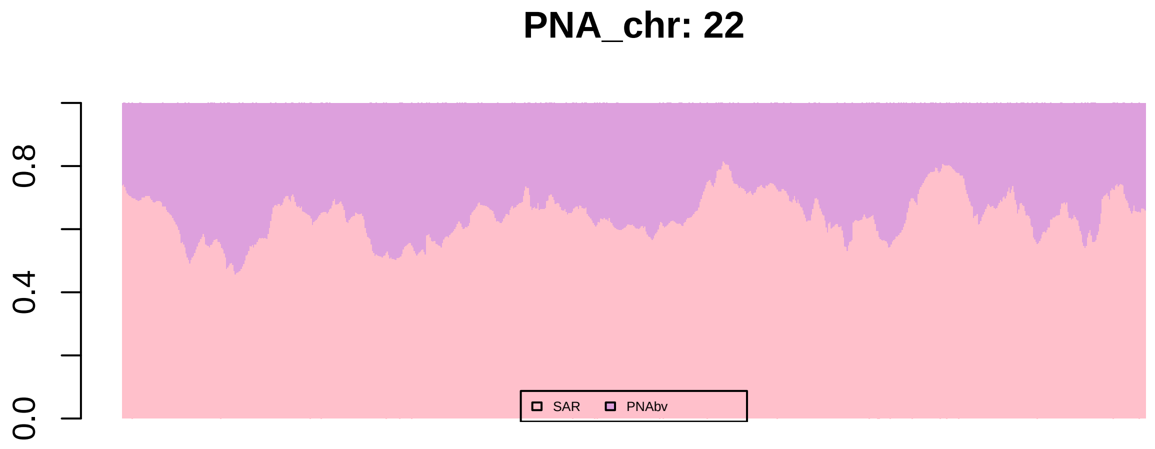
PNA_chr: 22
0.0
0.4
0.8
SAR
PNAbv

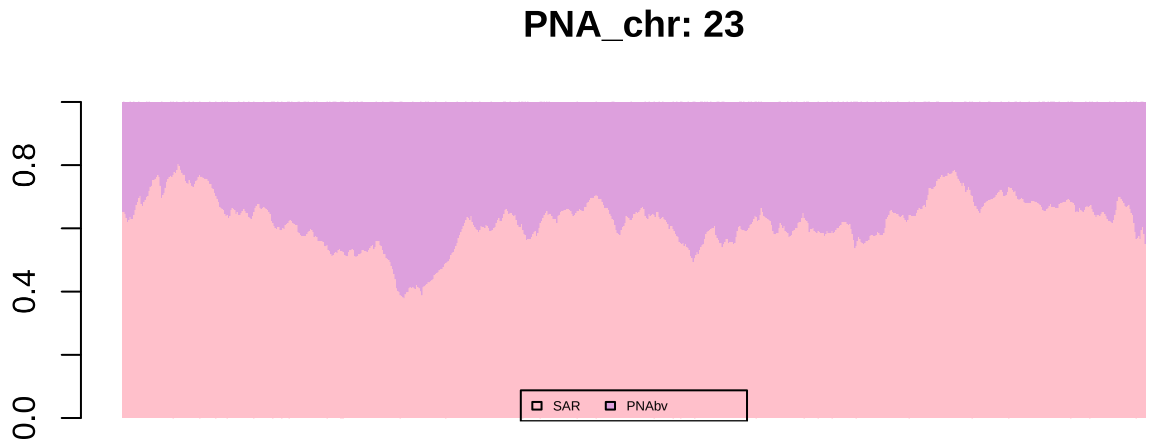
PNA_chr: 23
0.0
0.4
0.8
SAR
PNAbv

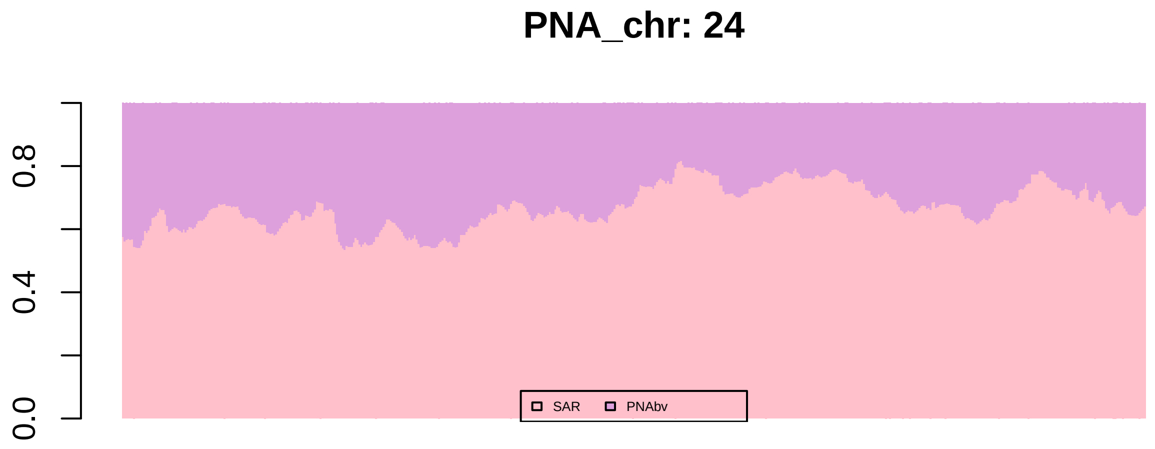
PNA_chr: 24
0.0
0.4
0.8
SAR
PNAbv

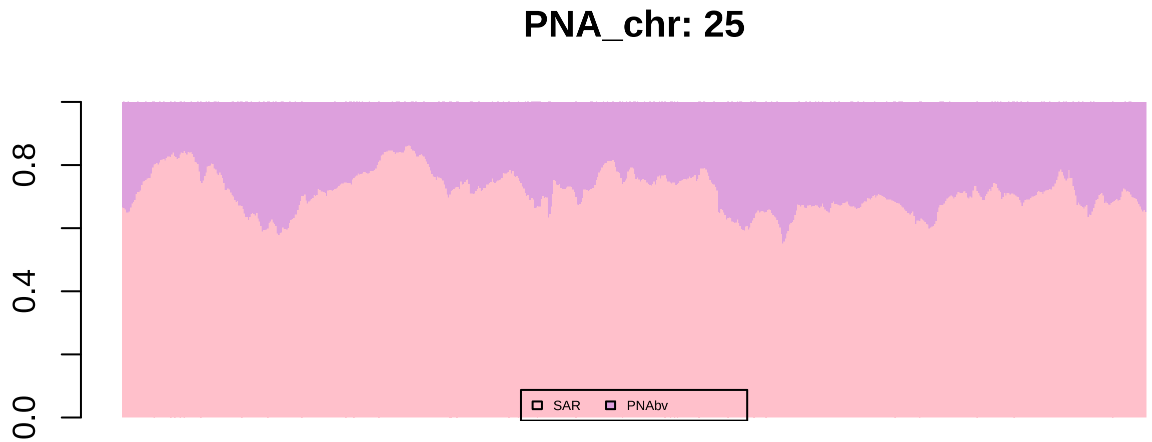
PNA_chr: 25
0.0
0.4
0.8
SAR
PNAbv

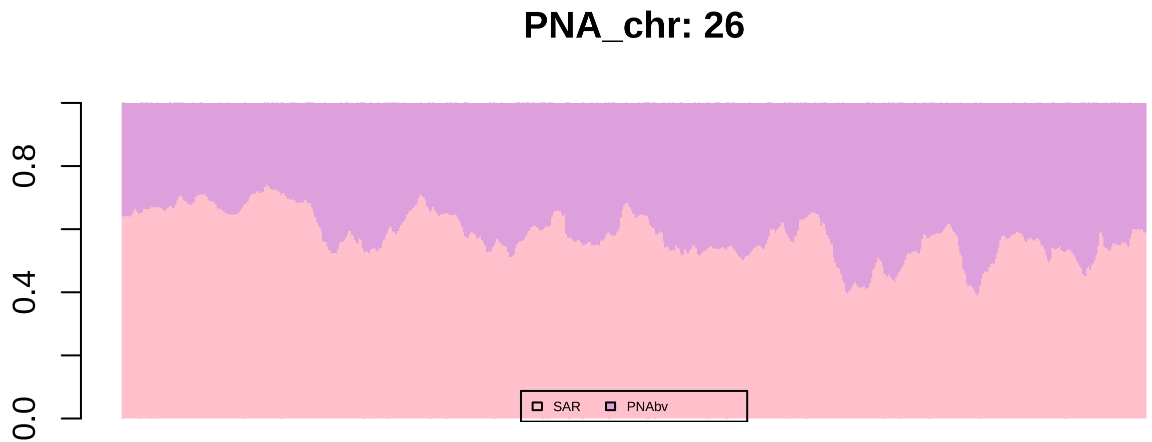
PNA_chr: 26
0.0
0.4
0.8
SAR
PNAbv
